# Supplementary material for: Algorithm-aided engineering of aliphatic halogenase WelO5* for the asymmetric late-stage functionalization of soraphens
Source: Nat Commun. 2022 Jan 18;13:371. doi: 10.1038/s41467-022-27999-1 (PMC8766452; doi:10.1038/s41467-022-27999-1)
Supplement: Supplementary file 1 — Supplementary Information [file 41467_2022_27999_MOESM1_ESM.pdf]

Supplementary Information

Algorithm-aided engineering of aliphatic halogenase WelO5* for the asymmetric late-stage functionalization of soraphens

Johannes Büchler,[a, b] Sumire Honda Malca[a], David Patsch,[a, c] Moritz Voss,[a] Nicholas J. Turner[b], Uwe T. Bornscheuer[c], Oliver Allemann[d, e], Camille Le Chapelain[d], Alexandre Lumbroso[d], Olivier Loiseleur*[d] and Rebecca Buller*[a]

[a] Competence Center for Biocatalysis, Institute of Chemistry and Biotechnology, Zurich University of Applied Sciences, Einsiedlerstrasse 31, 8820 Wädenswil, Switzerland, rebecca.buller@zhaw.ch
[b] School of Chemistry, The University of Manchester, Manchester Institute of Biotechnology, Manchester M1 7DN, United Kingdom
[c] Institute of Biochemistry, Dept. of Biotechnology & Enzyme Catalysis, Greifswald University, Felix-Hausdorff-Strasse 4, 17487 Greifswald, Germany
[d] Syngenta Crop Protection AG, Schaffhauserstrasse 101, 4332 Stein, Switzerland, olivier.loiseleur@syngenta.com
[e] Idorsia Pharmaceuticals Ltd, Hegenheimermattweg 91, 4123 Allschwil, Switzerland

Contents

I. Supplementary Tables 2
II. Supplementary Figures 8
III. Supplementary Methods 23
Chemical synthesis methods 23
Synthesis and characterization of soraphen C and soraphen analogues 24
Purification of bio-extracts 29
Analytical data of the halogenated and hydroxylated products 30
NMR spectra 33
Methods for biological testing and BP80 determination 58
Combinatorial library WelO5* 65
IV. Supplementary References 66

I. Supplementary Tables

Supplementary Table 1. Fl-Hal panel.

Natural enzymes	
Name	Source organism
Bmp5[1]	*P. luteoviolacea* 2ta16
PyrH[2]	*Streptomyces ru- gosporus* LL-42D005
KtzR[3]	*Kutzneria* sp. 744
Th-Hal[4]	*Streptomyces violaceusniger* SPC6
SttH[5]	*Streptomyces toxytricini* NRRL 15443
PrnA[6]	*Pseudomonas fluorescens*
KtzQ[3]	*Kutzneria* sp. 744
RebH[7]	*Lechevalieria aerocolonigenes* (strain 39243)
ThaL[8]	*Streptomyces albogriseolus*
PrnC[6]	*Pseudomonas fluorescens*
RadH[9]	*Chaetomium chiversii*
MalA'[10]	*Malbranchea graminicola* (086937A)
Rdc2[11]	*Pochonia chlamydosporia*
ChlA[12]	*Dictyostelium discoideum*
**Engineered enzymes**	
Name	Name
PrnA_F103A[13]	RebH_3SS[14]
PrnA_E450K_F454K[13]	RebH_4V[14]
SttH_Triple	RebH_5LS[15]
RebH_0S[15]	RebH_6TL[15]
RebH_1PVM[14]	RebH_8F[15]
RebH_2T[14]	RebH_10S[15]
RebH_3S[14]	RebH_Thermo[16]

Supplementary Table 2. αKGHs panel.

Natural enzymes	
Name	Source organism
WelO5[17]	*Hapalosiphon welwitschii* UTEX B1830
WelO5*[18]	*Hapalosiphon welwitschii* IC-52-3
AmbO5[19]	*Fischerella ambigua* UTEX1903
**Engineered enzymes**	
Name	Name
WelO5*_N74L	WelO5*_V81L
WelO5*_V81T	WelO5*_I84F
WelO5*_A88G	WelO5*_A88S
WelO5*_A88T	WelO5*_V90P
WelO5*_P153F	WelO5*_P153K
WelO5*_161A	WelO5*_I161D
WelO5*_I161G	WelO5*_I161R
WelO5*_I161S	WelO5*_I161T
WelO5*_I161E	WelO5*_I225M
WelO5*_E76V_V81L	WelO5*_ V81G_I161G
WelO5*_ V81G_I161P	WelO5*_ V81L_A88T
WelO5*_ V81L_I161D	WelO5*_ V81L_I161M
WelO5*_ V81L_I161V	WelO5*_ V81R_I161D
WelO5*_ V81R_I161G	WelO5*_ V81R_I161S
SadA_D157G[20]	

Supplementary Table 3. Ranking of the predicted variants using machine learning. Variants were predicted towards increase in activity and towards increase in selectivity. The produced "activity" variants were chosen on the highest activity predictions. The "selectivity for 1b" variants were chosen on high predicted selectivity towards product **1b** with the additional threshold that the activity predictions had to be higher than 0.6. The activity label (*A*) was calculated using the formula *A = tot. Cl conversion WelO5* variant / tot. Cl conversion WelO5* GAP* (*tot. Cl conversion* = (SIM_{1a} + SIM_{1b}) / (SIM_{1a} + SIM_{1b} + SIM_{1c} + SIM_{1})). The selectivity label (*S*) was calculated using the formula *S* = (SIM_{1a} – SIM_{1b}) / (SIM_{1a} + SIM_{1b}).

Activity				Selectivity 1b		
Mutant	Ranking activity	Activity p/m#	Selectivity p/m#	Mutant	Selectivity p/m#	Activity p/m#
SIP	1	10.2/10.4	0.32/0.08	AHS	-0.77/-0.75	0.7/2.7
VIA	2	9.3/11.4	0.32/0.23	MHS	-0.68/-0.77	1.2/0.9
AIP	3	9.2/11.2	0.07/0.08	AMS	-0.67/*	0.8/*
ALP	4	8.2/11.3	0.56/0.70	VHS	-0.60/-0.75	1.5/1.9
CIA	5	8.0/*	0.25/*	LHT	-0.55/-1.0	0.7/0.5
CIP	6	7.84/*	0.30/*	LHA	-0.53/-0.67	1.8/1.9
SVP	7	7.6/8.5	0.63/0.52	AHG	-0.52/-1.0	1.2/4.2
SIA	8	7.4/5.9	0.24/0.28	AHA	-0.52/*	1.2/*
CLP	9	7.2/*	0.74/*	LMS	-0.51/0.52	0.6/0.6
VLA	10	7.2/11.0	0.77/0.72	LHG	-0.51/-1.0	1.7/3.8

* these variants were not measured, #predicted/measured

Supplementary Table 4. Activity of WelO5* variants for the functionalization of soraphen derivatives. The halogenase variants were capable to produce multiple products (as reported). Each of the reported products were derivatized once only as observed by selected ion monitoring.

Compound	Observed products	WelO5* variant
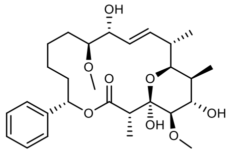 Soraphen C, **2**	- 2 chlorinated products<br>- 2 hydroxylated products	- VAA⁺<br>- ILV°
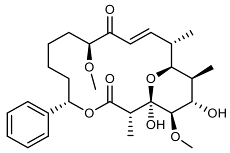 **3**	- 4 chlorinated products<br>- 3 hydroxylated products	- SLP⁺<br>- SLP°
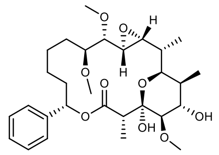 **4**	- 5 chlorinated products<br>- 2 hydroxylated products	- SLP⁺<br>- SHP°

⁺ Variant showing the highest amount of total chlorination; ° Variant showing the highest amount of total hydroxylation

Supplementary Table 5. Selected examples of the application of machine learning used for the engineering of enzymes.

Year	Enzyme	Target	Number of variants	Perc. Covered /%	Reference
2007	halohydrin dehalogenase	activity	30-150 at each round for 18 rounds	~$(3*N/2^N) * 100$ N= mutation sites	Fox et al.[21]
2012	epoxide hydrolase	enantioselectivity	95	23.8	Feng et al.[22]
2018	green flourescent protein	color change	218	0.14	Saito et al.[23]
2018	epoxide hydrolase	enantioselectivity	37	7.4	Cadet et al.[24]
2019	nitric oxide dioxygenase	stereodivergence	445 over 2 rounds	0.6 - 8.9	Wu et al.[25]
2021	artificial metalloenzymes	activity	400	80.0	Vornholt et al.[26]

Supplementary Table 6. Enzyme and substrate concentrations used for the kinetic experiments.

Variants	Enzyme conc. / µM	Substrate conc. / µM
GAP	20	40, 70, 100, 150, 200, 250,500, 750
VLA	2	40, 70, 100, 150, 200, 250,500, 750
SLP	2	40, 70, 100, 150, 200, 250,500, 750

Supplementary Equation 1. Substrate inhibition model.

$$v_0 = \frac{v_{max} * [S]_0}{K_m + [S]_0 + \frac{[S]_0^2}{K_i}}$$

Supplementary Table 7. Comparison of ^{13}C-NMR chemical shifts between soraphen C (reported[2] and synthesized) and synthesized epi-soraphen C.

^{13}C-NMR shifts of fermented soraphen C (δ in ppm)[2]	^{13}C-NMR shifts of synthesized soraphen C (δ in ppm)	Δ1 (ppm)	^{13}C-NMR shifts of synthesized epi-soraphen C (δ in ppm)	Δ2 (ppm)
170.6	170.78	-0.2	172.82	-2.2
141.0	141.12	-0.1	139.69	1.3
137.3	137.45	-0.1	137.96	-0.7
128.6	128.72	-0.1	128.76	-0.2
128.2	128.29	-0.1	128.70	-0.5
126.2	126.35	-0.1	128.46	-2.3
125.0	125.18	-0.2	127.09	-2.1
99.4	99.60	-0.2	99.91	-0.5
83.7	83.89	-0.2	82.86	0.8
76.1	76.28	-0.2	77.57	-1.5
74.9	75.04	-0.1	76.33	-1.4
74.6	74.81	-0.2	73.58	1.0
72.5	72.66	-0.2	71.74	0.8
68.8	68.98	-0.2	68.93	-0.1
57.6	57.78	-0.2	57.49	0.1
57.3	57.47	-0.2	57.45	-0.2
46.2	46.35	-0.1	45.42	0.8
35.8	36.01	-0.2	36.84	-1.0
35.6	35.79	-0.2	35.46	0.1
35.2	35.32	-0.1	35.02	0.2
29.4	29.57	-0.2	27.58	1.8
26.0	26.14	-0.1	25.12	0.9
23.0	23.17	-0.2	22.57	0.4
12.5	12.64	-0.1	16.57	-4.1
11.7	11.83	-0.1	12.55	-0.9
10.3	10.49	-0.2	10.55	-0.3

II. Supplementary Figures

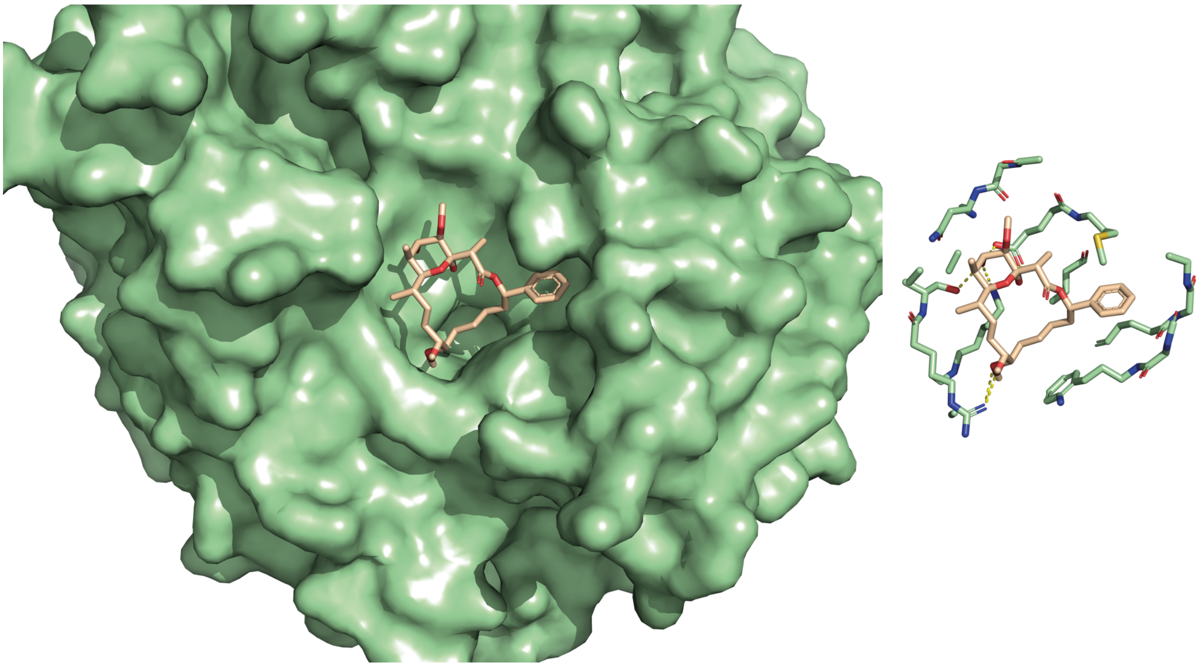

Supplementary Figure 1. Crystal structure of soraphen A bound to the BC domain of yeast acetyl-coenzyme A carboxylases. The crystal structure (green) reveals the active conformation of the macrocycle (PDB ID: 1W96). Right: Visualization of the interactions between the soraphen A (wheat sticks) and the residues of the BC domain (green sticks).

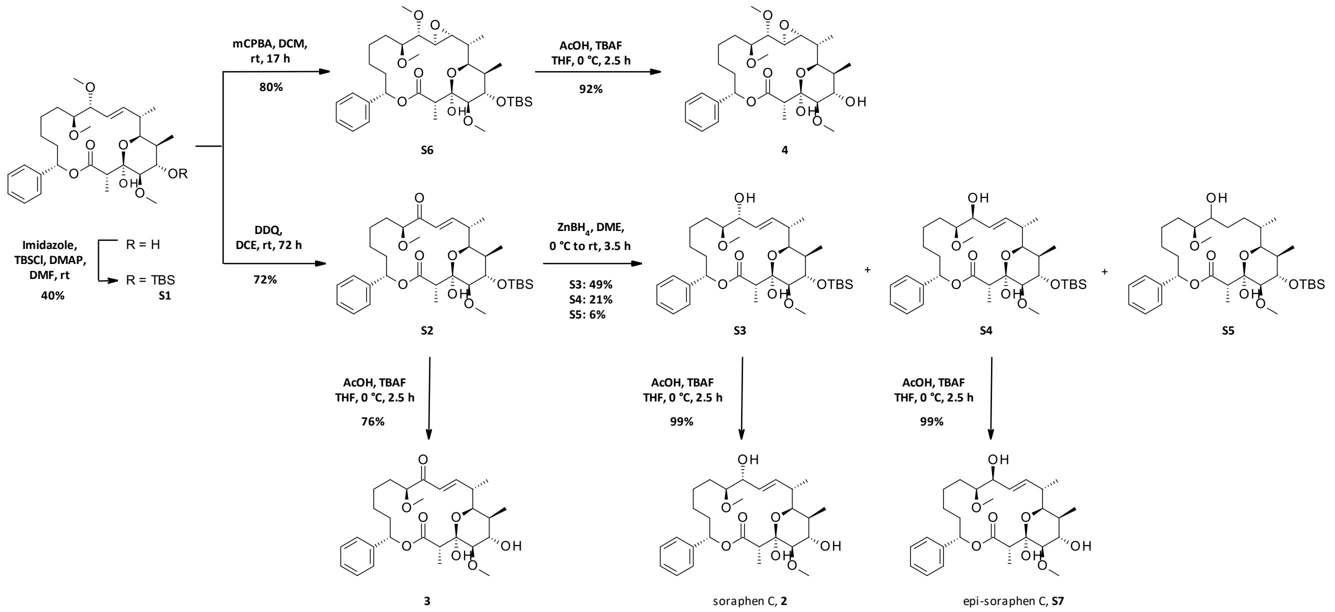

Supplementary Figure 2. Synthesis scheme to obtain soraphen C and soraphen analogues. Individual reaction steps are described in the Supplementary Methods.

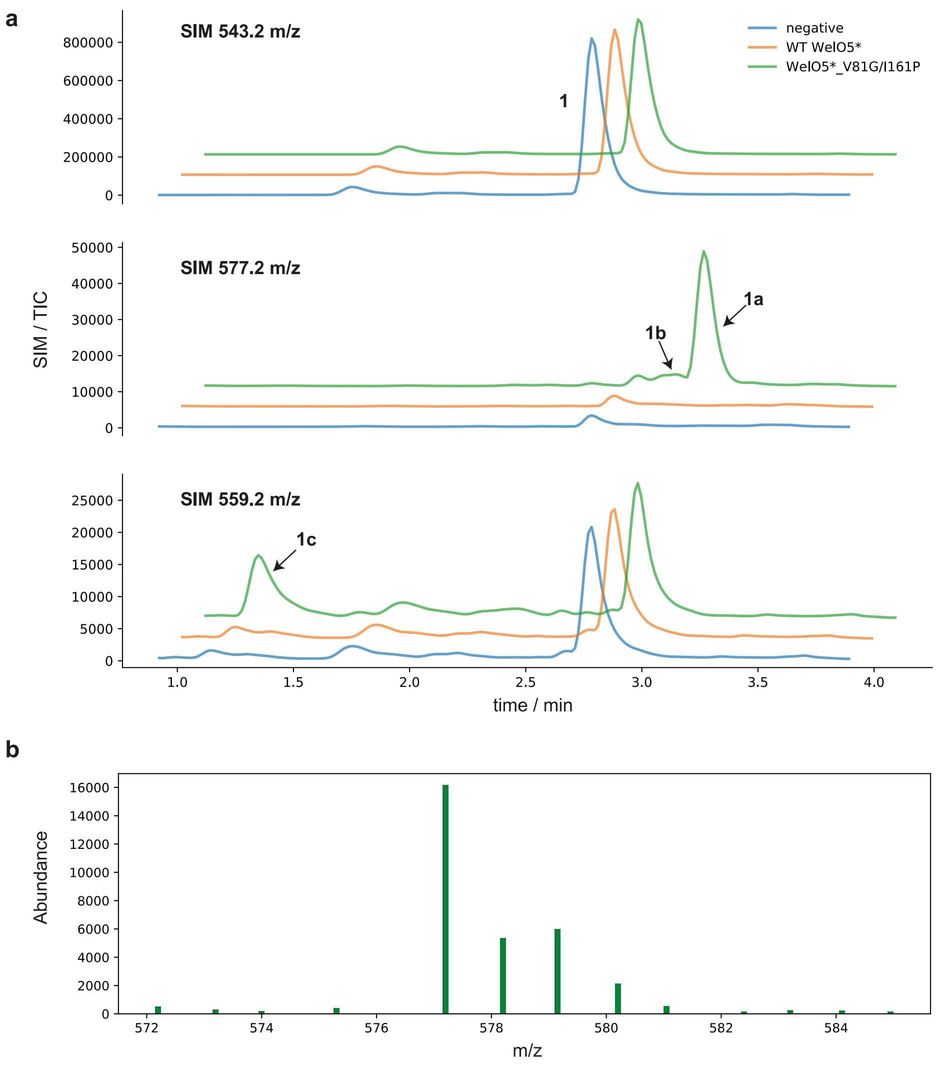

Supplementary Figure 3. LC-MS analysis of the biotransformation of soraphen A. a Selected ion chromatograms (SIM) of the m/z values of interest. Biotransformation using negative control (blue), WT WelO5* (orange) and WelO5*_V81G_I161P (green) are compared. The top chromatogram shows the trace of soraphen A (543.2 m/z = **1**+Na+H^+), the middle chromatogram shows two species corresponding to chlorinated soraphen A (577.2 m/z = **1**+Na+H^++Cl^{35}) and the bottom chromatogram shows one hydroxylated soraphen A species (577.2 m/z = **1**+Na+H^++OH). **b** MS chart of the chlorinated species showing the characteristic M: M + 2 = 3 : 1 isotopic pattern of a chlorinated compound.

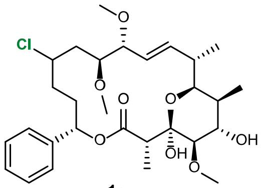

1a

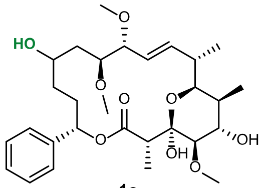

1c

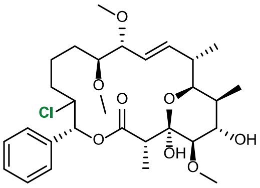

1b

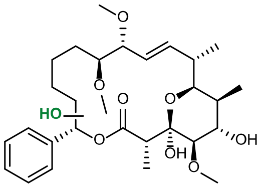

1d

Supplementary Figure 4. Biotransformation products of soraphen A. Observed products of the biotransformation reactions of soraphen A with WelO5* variants.

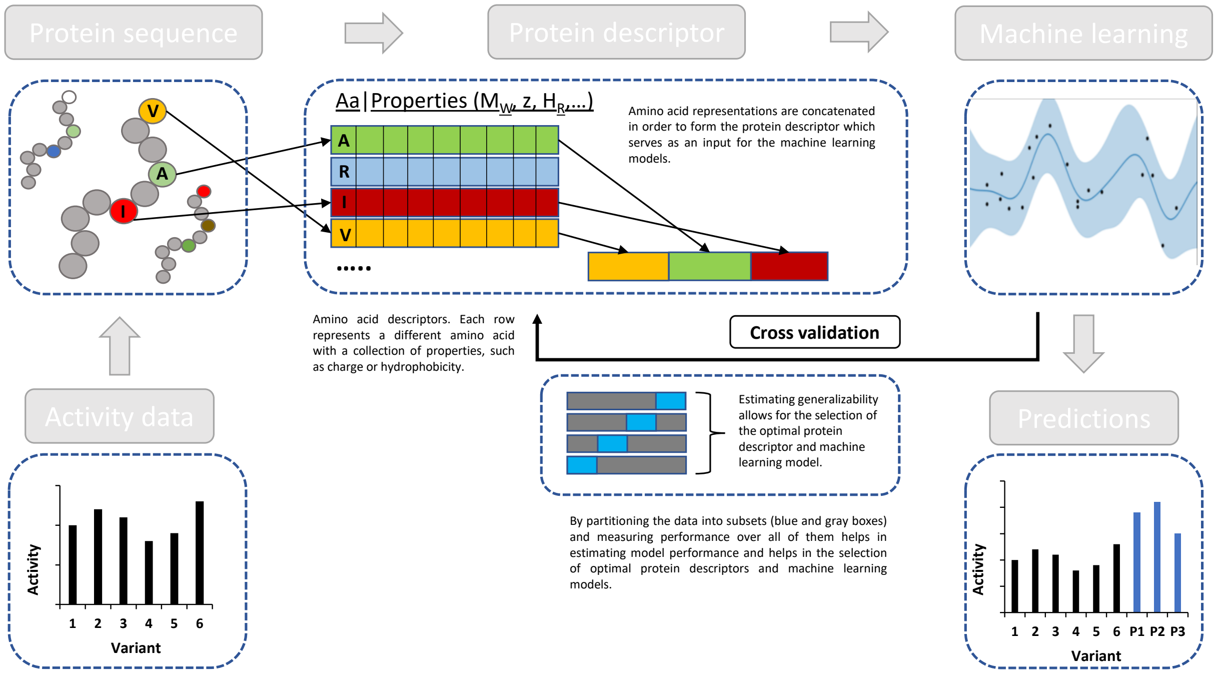

Supplementary Figure 5. Illustration of the algorithm aided approach to predict improved variants. Activity (or selectivity) data obtained by LC-MS analysis was used as a label for the machine learning algorithm. Amino acids properties were represented as a 17-dimensional vector. The feature vector of a sequence was defined by joining the vector representation of its individual amino acids at sites V81X, A88X, I161X and aggregated into the *504 x 51*-dimensional training matrix. This was used to train a machine learning model. To avoid overfitting and to better gauge the generalizability of our model, we cross-validated over ten splits, and model performance was evaluated on the coefficient of determination (R^2).

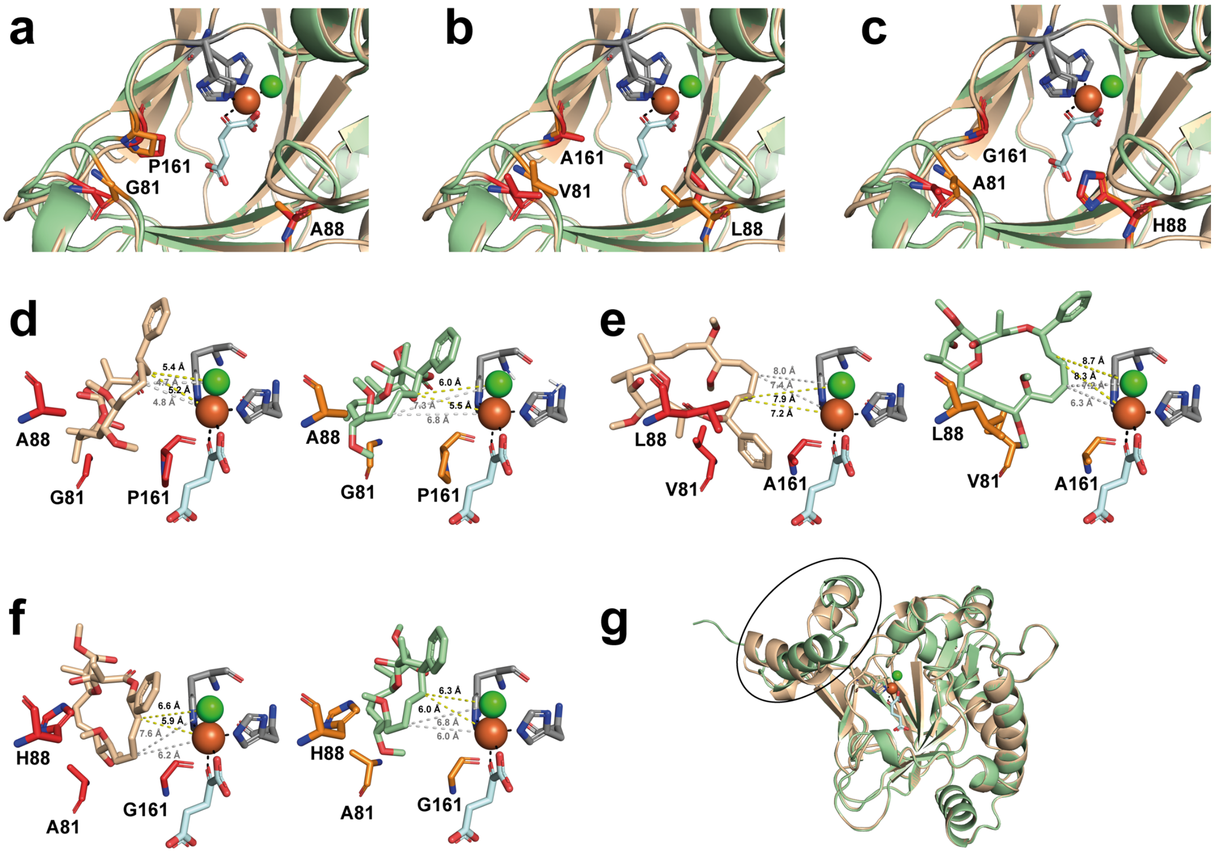

Supplementary Figure 6. Docking studies of soraphen A into WelO5* homology models. Enzyme models were prepared with SWISS-MODEL[27] (enzyme model and soraphen A in wheat, engineered residues in red) or AlphaFold[28] (enzyme model and soraphen A in palegreen, engineered residues in orange) and soraphen A was docked using AutoDock Vina[29]. In the active site the histidines coordinating to the iron (orange) are shown in grey, the chlorine in green and the α-ketoglutarate in pale cyan. **a-c** View into the active site of the WelO5* variants GAP, VLA and AHG, respectively. The active site of WelO5* including the engineered residues at position 81, 88 and 161 are nearly identical in both homology models (SWISS-MODEL (wheat) and AlphaFold (palegreen)). **d-f** Soraphen A docked into the WelO5* variants GAP, VLA and AHG. Distances were measured from the iron and chloride to C14 of soraphen A (grey dotted lines) and to C16 of soraphen A (yellow dotted lines). The two models of **d** WelO5* GAP and **f** WelO5* AHG show a similar positioning of the engineered amino acid residues as well as of the docked soraphen A. In the AlphaFold model of **e** WelO5* VLA shorter distances between the iron and chloride to C14 of soraphen A (grey dotted lines) than to C16 of the macrolide (yellow dotted lines) suggest the structural reason for the predominant formation of regioisomer **1a**. **g** Overall structural homology of the enzyme models prepared with SWISS-MODEL (wheat) or AlphaFold (palegreen): Main structural differences lay in the two α-helices marked in the black circle.

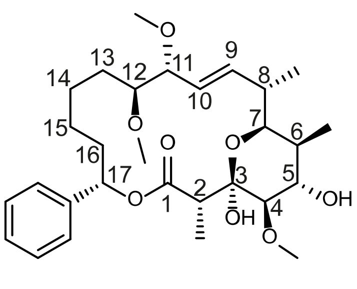

Supplementary Figure 7. Structure of soraphen A. Numbering of the carbon atoms of the cyclic polyketide backbone of soraphen A.

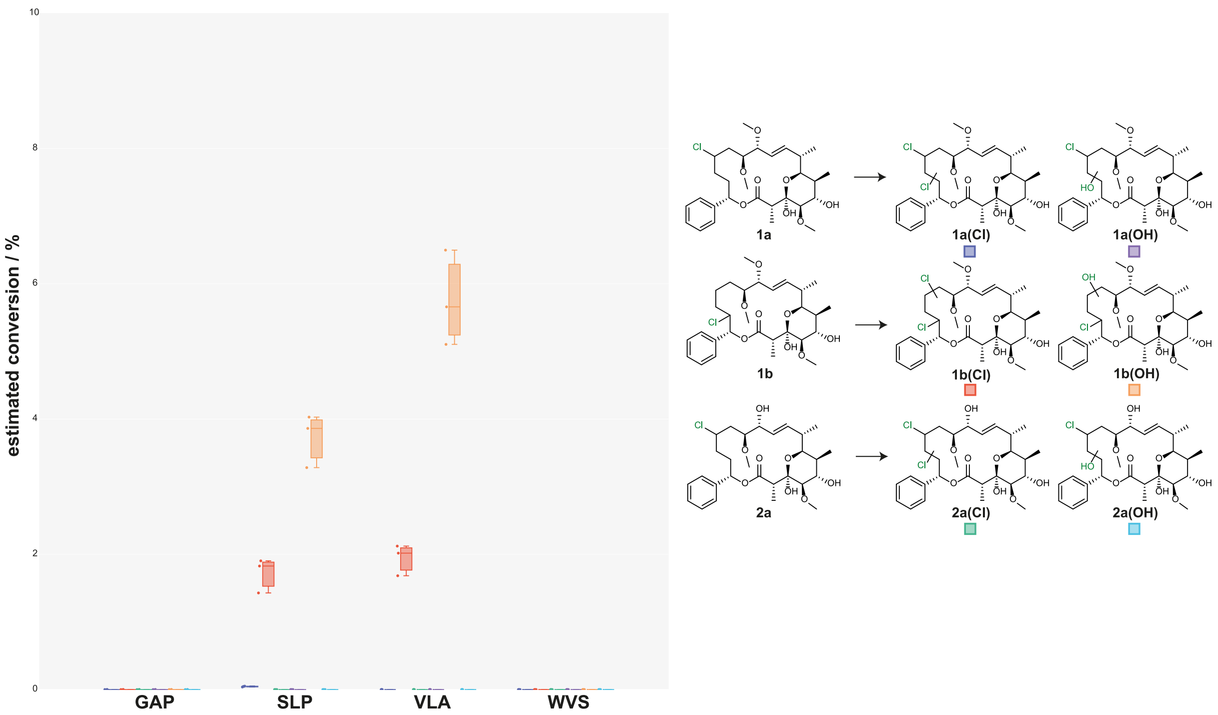

Supplementary Figure 8. *In vitro* activity assays using mono-chlorinated products (1a, 1b and 2a) as substrates for the engineered WelO5* variants GAP, SLP, VLA and WVS. Depicted is the estimated conversion to chlorinated or hydroxylated product (*SIM area of product / SIM area of all products and starting material* * 100). Reactions were performed according to the method described in the main paper using an enzyme concentration of 5 µM for variants GAP, SLP, VLA and WVS and a substrate concentration of 60 µM (1a, 1b and 2a). The conversion values to the chlorinated or hydroxylated products by the engineered halogenase variants were determined in triplicates (N = 3 independent experiments). The depicted boxes correspond to the interquartile range and end at the quartiles Q_1 and Q_3, respectively. The statistical median is depicted as a horizontal line in the box. The whiskers comprise the farthest points that are not outliers (i.e., that are within 1.5x of the interquartile range of Q_1 and Q_3, respectively).

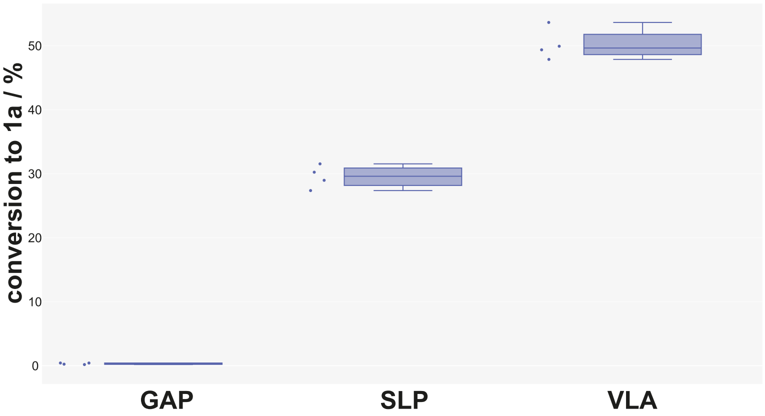

Supplementary Figure 9. *In vitro* activity assays showing the conversion of soraphen A to the chlorinated product 1a by the engineered WelO5* variants GAP, SLP and VLA. The conversion was determined at a substrate concentration of 60 µM and the reaction was quenched at stable product concentration using the procedure described in the method section of the main paper. To account for the different enzyme concentrations used (GAP = 5 µM, SLP = 0.5 µM and VLA = 0.5 µM), the observed product concentration in the GAP reactions was divided by ten. The conversion values of soraphen A to the chlorinated product **1a** by the engineered halogenase variants were determined in quadruplicates in each case (N = 4 independent experiments). The depicted boxes correspond to the interquartile range and end at the quartiles Q_1 and Q_3. The statistical median is depicted as a horizontal line in the box. The whiskers comprise the farthest points that are not outliers (i.e., that are within 1.5x the interquartile range of Q_1 and Q_3, respectively).

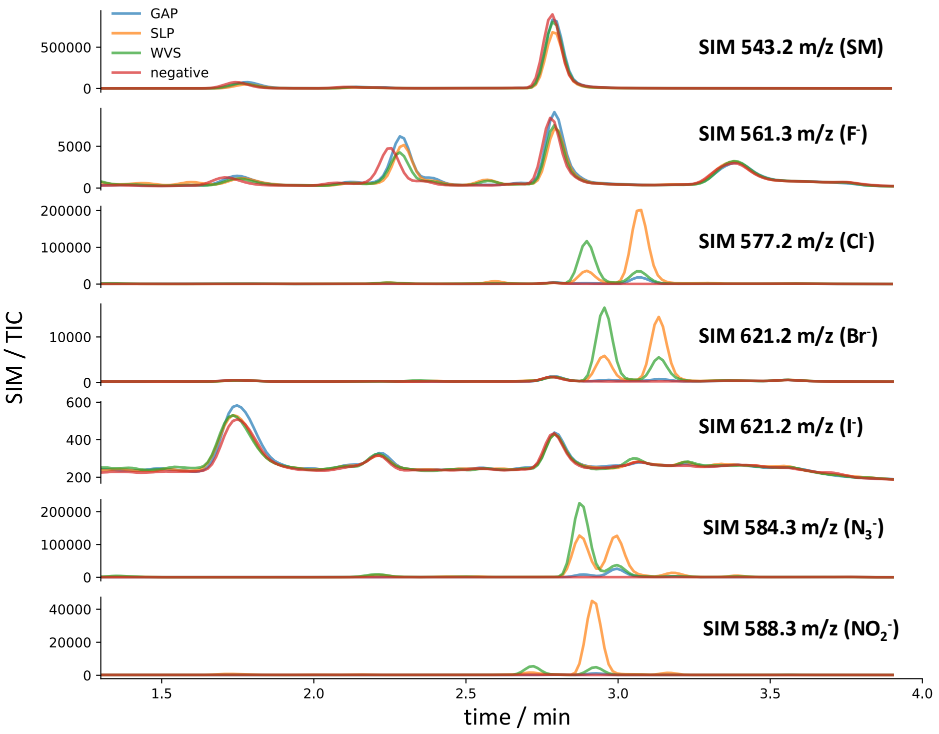

Supplementary Figure 10. LC-MS analysis of the anion promiscuity of selected WelO5* variants. The variants GAP (blue), SLP (orange), WVS (green) and negative control (no enzyme, red) were analysed in the presence of 500 mM of NaF, NaCl, NaBr, NaI, NaN_3 and $NaNO_2$, respectively, by selected ion monitoring. Masses corresponding to the introduction of the anions into soraphen A could be observed for chloride, bromide, azide and nitrite salts.

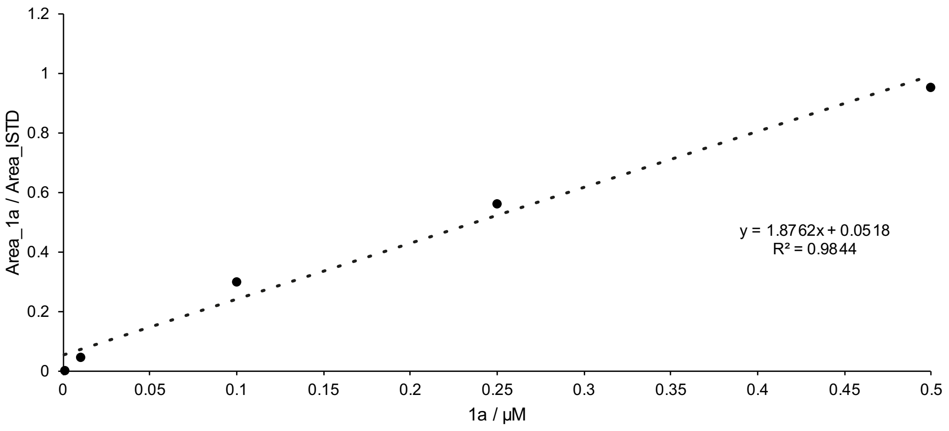

Supplementary Figure 11. Calibration curve of 1a used for product quantification.

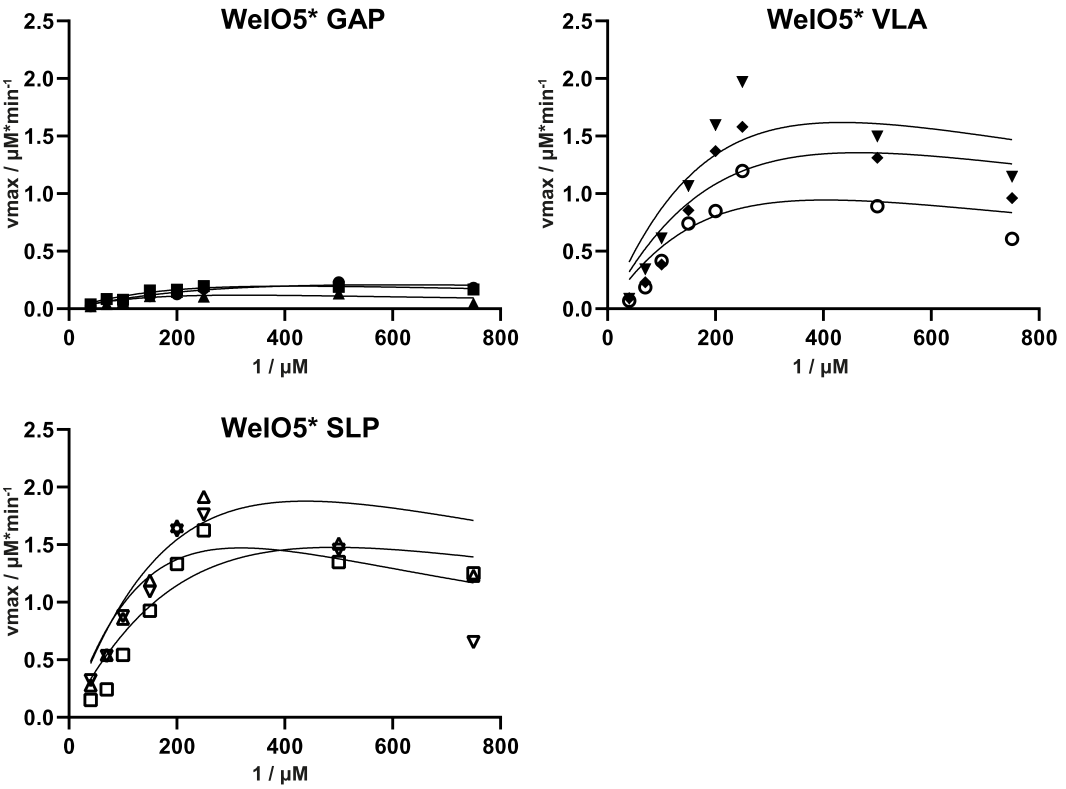

Supplementary Figure 12. Michaelis Menten kinetics for WelO5* variants GAP, VLA and SLP. The formation of product **1a** for the WelO5* variants GAP, VLA and SLP was measured in triplicate at each substrate concentration (N=3 independent experiments). All data points belonging to individual Michaelis Menten measurement series are marked by triangles, squares or circles. Substrate inhibition can be observed in all cases.

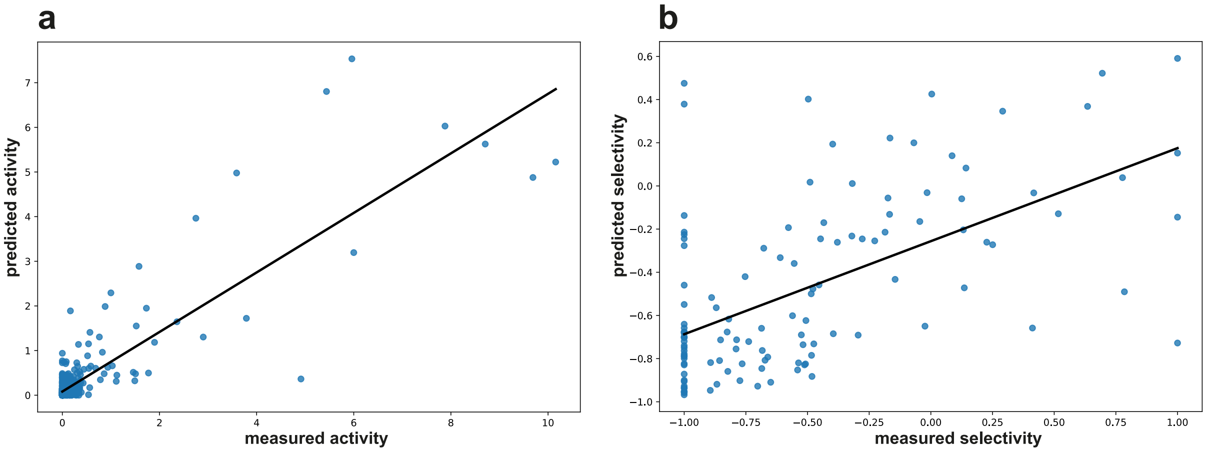

Supplementary Figure 13. Out of fold predicted vs measured values. a The measured activity values of the training set were predicted using Gaussian processes (y-axis) and compared to the measured activity (x-axis) **b** The measured selectivity of the training set were predicted using a random forest algorithm (y-axis) and compared to the measured selectivity (x-axis). A linear regression is shown for these values.

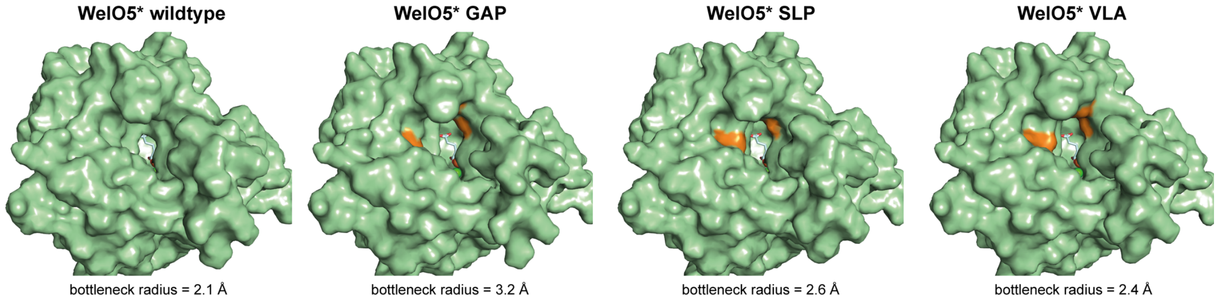

Supplementary Figure 14. Overview of the AlphaFold models (green) of WelO5* and the variants GAP, SLP and VLA showing a view of the entrance to the active site. The engineered amino acid residues are shown in orange, while the co-factor α-ketoglutarate is shown in light cyan. To comparatively evaluate substrate access to the active sites of all enzyme variants, the bottleneck radii were calculated using CAVER Web 1.0 with default parameters[30]. This investigation highlighted that the employed enzyme engineering approach has led to a widening of the access tunnel from 2.1 Å (wildtype enzyme) to 3.2 Å (variant GAP), 2.6 Å (variant SLP) and 2.4 Å (variant VLA). The resulting improved access to the active site might explain why the wildtype enzyme cannot convert the macrolide soraphen A, while variants GAP, SLP and VLA accept the bulky substrate.

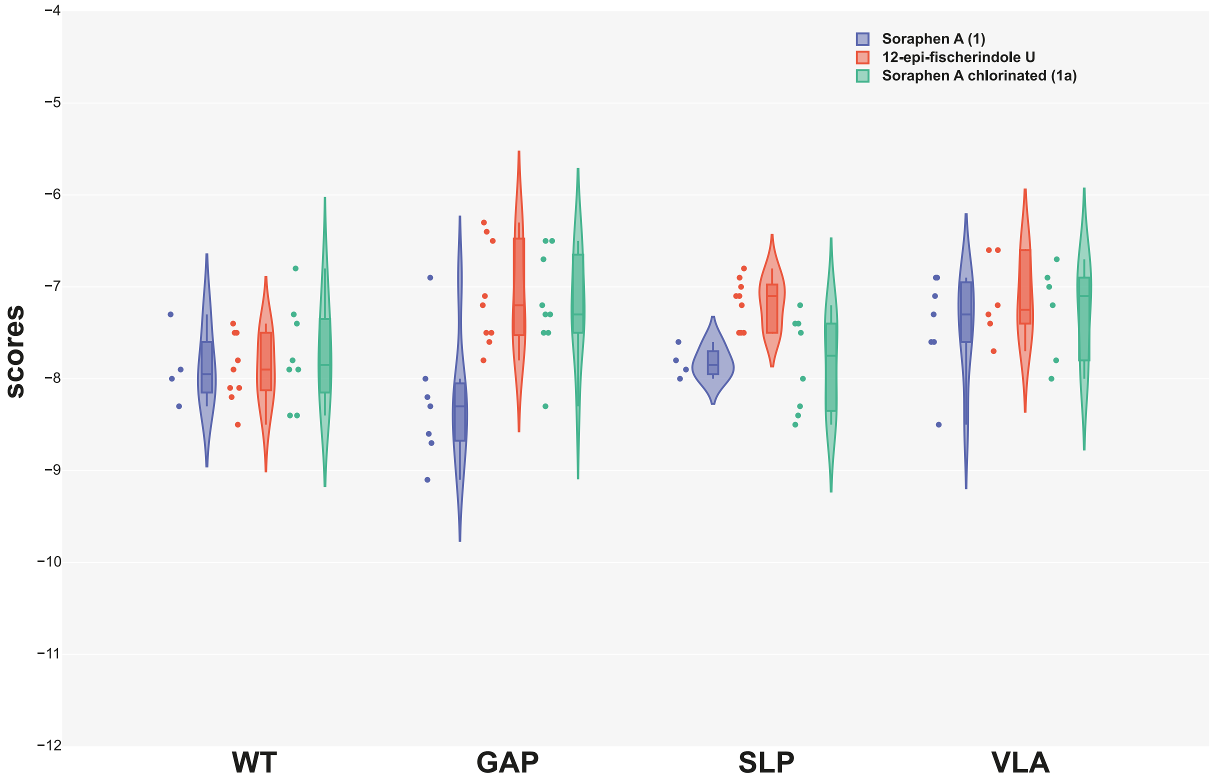

Supplementary Figure 15. Docking experiments of soraphen A, 12-epi-fischerindole U and chlorinated soraphen A into wildtype WelO5* and its engineered variants. Overview of the docking scores of soraphen A (blue, **1**), the natural substrate 12-epi-fischerindole U (red) and chlorinated soraphen A (green, **1a**) into AlphaFold models of WelO5* wild type (WT) and the variants GAP, SLP and VLA*,* respectively. The docking was performed as described in the method section of the main paper. All ligands, irrespective of them being native or non-native substrates, showed similar docking scores for the enzyme variants. Scores were obtained through the AutoDock Vina scoring function[32] which consists of the weighted sum of steric interactions ($gauss_1$, $gauss_2$ and repulsion, identical for all atom pairs), hydrophobic interaction between hydrophobic atoms and hydrogen bonding (where applicable). A lower score indicates higher affinity of the ligand towards the receptor. In each case, nine docking solutions were obtained from one docking experiment (n = 1 individual experiment). Results were visually inspected using PyMOL software and only solutions in which the ligand docked close to the active site were considered in the depicted analysis. The depicted boxes correspond to the interquartile range and end at the quartiles Q_1 and Q_3. The median is depicted as a horizontal line in the box. The whiskers comprise the farthest points that are not outliers (i.e., that are within 1.5x the interquartile range of Q_1 and Q_3, respectively).

III. Supplementary Methods

Chemical synthesis methods

Synthesis: Soraphen A was obtained by fermentation at Syngenta (former Novartis) using the published procedure[31]. The reagents for synthesis were obtained from commercial sources and used without further purification unless otherwise stated. The solvents for synthesis were obtained from commercial sources and stored over molecular sieves.

Purification: purification over silica gel were performed on a Combi*Flash* Rf 200i instrument using standard commercial pre-packed silica gel cartridges.

NMR: NMRs were recorded either on a Bruker 400 MHz spectrometer or a Bruker 600 MHz spectrometer. ^{1}H-NMR chemical shifts are reported relative to TMS and are referenced based on the residual proton resonances of the corresponding deuterated solvent ($CDCl_3$: 7.26 ppm) whereas ^{13}C NMR spectra are reported relative to TMS using the carbon signals of the deuterated solvent ($CDCl_3$: 77.16 ppm). Assignments were made on the basis of chemical shifts, coupling constants, COSY, HSQC, HMBC, ROESY data. Resonances are described using the following abbreviations; s (singlet), d (doublet), t (triplet), q (quartet), quin. (quintet), sext. (sextet), sept. (septet), m (multiplet), br. (broad), app. (apparent), dd (double doublet) and so on. Coupling constants (J) are given in Hz and are rounded to the nearest 0.1 Hz.

HPLC-MS: HPLC traces were obtained on an Acquity UPLC from Waters: Binary pump, heated column compartment, diode-array detector and ELSD detector. Column: Waters UPLC HSS T3, 1.8 µm, 30 x 2.1 mm, Temp: 60 °C, DAD Wavelength range: 210 to 500 nm, Solvent Gradient: A = water + 5% MeOH + 0.05 % HCOOH, B= Acetonitrile + 0.05 % HCOOH, gradient: 10-100% B in 2.7 min; Flow: 0.85 mL/min. Low resolution mass spectra were recorded on a mass spectrometer from Waters (SQD, SQDII Single quadrupole mass spectrometer) equipped with an electrospray source (Polarity: positive and negative ions); Capillary: 3.00 kV, Cone range: 30V, Extractor: 2.00 V, Source Temperature: 150 °C, Desolvation Temperature: 350 °C, Cone Gas Flow: 50 L/h, Desolvation Gas Flow: 650 L/h, Mass range: 100 to 900 Da.

Synthesis and characterization of soraphen C and soraphen analogues

For a scheme of the synthesis route refer to Supplementary Figure 2.

(1*R*,2*S*,5*S*,10*S*,11*R*,12*E*,14*S*,15*S*,16*R*,17*S*,18*R*)-17-[tert-butyl(dimethyl)silyl]oxy-1-hydroxy-10,11,18-trimethoxy-2,14,16-trimethyl-5-phenyl-4,19-dioxabicyclo[13.3.1]nonadec-12-en-3-one (**S1**)

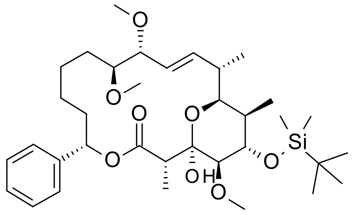

Soraphen A (100 mg, 0.19 mmol), DMAP (25 mg, 0.20 mmol), imidazole (52 mg, 0.77 mmol) and TBSCl (58 mg, 0.38 mmol) were dissolved in DMF (2.0 mL). The slightly yellow solution was stirred at room temperature for 48 h. Then the reaction mixture was diluted with EtOAc and washed with aq HCl (1M), water and brine, dried over Na_2SO_4, filtered and concentrated under reduced pressure.
The crude was purified by flash chromatography on silica gel (gradient: cHex/EtOAc 95:5 to 55:45, 30 mL/min, 15 min) to afford the title compound (60 mg, 49%) as well as some recovered starting material (50 mg).

^{1}H-NMR (400 MHz, $CDCl_3$): δ (ppm) = 7.42–7.23 (m, 5H), 6.40 (dd, *J* = 16.2, 3.8 Hz, 1H), 6.12 (dd, *J* = 12.0, 2.5 Hz, 1H), 5.41 (ddd, *J*=16.0, 9.5, 1.8 Hz, 1H), 5.17 (d, *J*=1.8 Hz, 1H), 4.17 (t, *J*=2.5 Hz, 1H), 3.78 (dd, *J*=9.5, 2.2 Hz, 1H), 3.69 (dd, *J*=10.5, 2.5 Hz, 1H), 3.47 (s, 3H), 3.42 (dt, *J*=11.1, 2.5 Hz, 1H), 3.38 (s, 3H), 3.31 (s, 3H), 3.05 (qd, *J*=7.0, 1.1 Hz, 1H), 3.00 (dd, *J*=2.7, 0.9 Hz, 1H), 2.56–2.49 (m, 1H), 2.13–2.04 (m, 1H), 1.81–1.72 (m, 2H), 1.65–1.54 (m, 3H), 1.34–1.25 (m, 2H), 1.16 (d, *J*=7.3 Hz, 3H), 1.09 (d, *J*=7.3 Hz, 3H), 1.05–1.01 (m, 1H), 1.00 (d, *J*=6.5 Hz, 3H), 0.94 (s, 9H), 0.17 (d, *J*=4.0 Hz, 6H);
^{13}C-NMR (101 MHz, $CDCl_3$): δ (ppm) = 171.25, 142.77, 140.74, 128.43 (2C), 127.52, 126.29 (2C), 121.80, 99.45, 84.99, 83.65, 77.39, 72.08, 71.90, 70.86, 58.32, 57.41, 56.31, 46.32, 37.30, 35.62, 35.22, 31.02, 25.95 (3C), 25.23, 24.18, 18.26, 12.42, 11.49, 10.32, –4.82, –4.85.

(1*R*,2*S*,5*S*,10*S*,12*E*,14*S*,15*S*,16*R*,17*S*,18*R*)-17-[tert-butyl(dimethyl)silyl]oxy-1-hydroxy-10,18-dimethoxy-2,14,16-trimethyl-5-phenyl-4,19-dioxabicyclo[13.3.1]nonadec-12-ene-3,11-dione (**S2**)

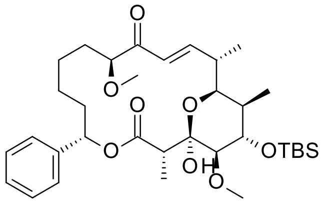

To a solution of **S1** (750 mg, 1.18 mmol) in 1,2-dichloroethane (14.8 mL) at room temperature was added DDQ (621 mg, 2.60 mmol). The yellow suspension was stirred for 4 days. Then the reaction mixture was diluted with EtOAc, washed twice with aq. $Na_2S_2O_3$ (10%), then brine, dried over Na_2SO_4, filtered and concentrated under reduced pressure.
The crude was purified by flash chromatography on silica gel (gradient: cHex/EtOAc 90:10 to 80:20, 40 mL/min, 16 min) to afford the title compound (522.8 mg, 72%).

^{1}H-NMR (400 MHz, $CDCl_3$): δ (ppm) = 7.45 (dd, *J*=16.9, 4.8 Hz, 1H), 7.39–7.28 (m, 4H), 6.24 (dd, *J*=16.9, 1.5 Hz, 1H), 5.91 (dd, *J*=8.6, 5.3 Hz, 1H), 5.05 (d, *J*=1.5 Hz, 1H), 4.29 (dd, *J*=7.7, 5.5 Hz, 1H), 4.16 (t, *J*=2.6 Hz, 1H), 3.86 (dd, *J*=10.3, 2.6 Hz, 1H), 3.37 (s, 6H), 3.05 (qd, *J*=7.0, 1.47 Hz, 1H), 3.00 (dd, *J*=2.8, 0.9 Hz, 1H), 2.70–2.64 (m, 1H), 2.03–1.94 (m, 1H), 1.82–1.71 (m, 2H), 1.70–1.58 (m, 4H), 1.49–1.39 (m, 3H), 1.13 (d, *J*=7.0 Hz, 3H), 1.10 (d, *J*=7.3 Hz, 3H), 1.00 (d, *J*=6.6 Hz, 3H), 0.94 (s, 9H), 0.16 (d, *J*=5.1 Hz, 6H);
^{13}C-NMR (101 MHz, $CDCl_3$): δ (ppm) = 202.74, 171.44, 152.37, 141.78, 128.45 (2C), 127.67, 127.22, 126.64 (2C), 99.90, 83.63, 74.30, 70.76, 70.61, 57.92, 57.50, 46.31, 36.52, 36.01, 35.73, 32.33, 25.92 (3C), 23.98, 23.49, 18.23, 13.20, 11.55, 10.24, –4.82, –4.86;
HPLC-MS: rt = 2.69 min, m/z = 504 $[M-C_6H_{14}Si]^-$; 618 $[M-H]^-$

2*S*,5*S*,10*S*,12*E*,14*S*,15*S*,16*S*,17*S*,18*R*)-1,17-dihydroxy-10,18-dimethoxy-2,14,16-trimethyl-5-phenyl-4,19-dioxabicyclo[13.3.1]nonadec-12-ene-3,11-dione (**3**)

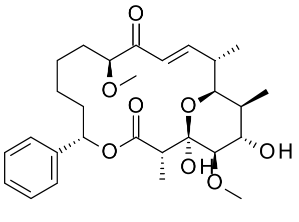

To a solution of **S2** (30.0 mg, 0.485 mmol) in THF (0.2 mL) were added at 0°C AcOH (8.8 µL, 0.15 mmol) and TBAF (0.10 mL, 1 M in THF, 0.10 mmol). The reaction mixture was stirred at 0 °C for 1.5 h. The reaction mixture was poured into sat. aq. NH_4Cl, then extracted with DCM/MeOH (9:1) The combined organic layers were dried over Na_2SO_4, filtered and concentrated under reduced pressure.
The crude product was purified by flash chromatography on silica gel (gradient: cHex/EtOAc 85:15 to 50:50, 18 mL/min, 12 min) to afford the title compound (21 mg, 86%).

^{1}H-NMR (400 MHz, $CDCl_3$): δ (ppm) = 7.35–7.30 (m, 6H), 6.51 (dd, *J*=16.1, 1.5 Hz, 1H), 5.61 (t, *J*=7.3 Hz, 1H), 4.51 (s, 1H), 4.04–4.01 (m, 2H), 3.80 (dd, *J*=9.2, 4.4 Hz, 1H), 3.38 (s, 3H), 3.35 (s, 3H), 3.17–3.12 (m, 2H), 2.69–2.60 (m, 1H), 1.99–1.93 (m, 1H), 1.90–1.84 (m, 2H), 1.80–1.60 (m, 3H), 1.51–1.41 (m, 2H), 1.36–1.29 (m, 1H), 1.25–1.17 (m, 1H), 1.07 (d, *J*=7.3 Hz, 3H), 1.06 (d, *J*=7.3 Hz, 3H), 1.04 (d, *J*=7.0 Hz, 3H);
^{13}C-NMR (101 MHz, $CDCl_3$): δ (ppm) = 202.60, 170.99, 152.95, 140.15, 128.67 (2C), 128.31, 126.68 (2C), 124.57, 99.85, 86.02, 76.62, 76.16, 72.63, 68.99, 57.76, 57.62, 45.76, 36.53, 35.65, 34.44, 30.18, 23.74, 22.77, 13.94, 11.79, 10.49;
HPLC-MS: rt = 1.83 min, m/z = 504 $[M-H]^-$; 528 $[M+H+Na]^{2+}$

(1*R*,2*S*,5*S*,10*S*,11*R*,12*E*,14*S*,15*S*,16*R*,17*S*,18*R*)-17-[tert-butyl(dimethyl)silyl]oxy-1,11-dihydroxy-10,18-dimethoxy-2,14,16-trimethyl-5-phenyl-4,19-dioxabicyclo[13.3.1]nonadec-12-en-3-one (S3)
(1*R*,2*S*,5*S*,10*S*,11*S*,12*E*,14*S*,15*S*,16*R*,17*S*,18*R*)-17-[tert-butyl(dimethyl)silyl]oxy-1,11-dihydroxy-10,18-dimethoxy-2,14,16-trimethyl-5-phenyl-4,19-dioxabicyclo[13.3.1]nonadec-12-en-3-one (S4)
(1*R*,2*S*,5*S*,10*S*,14*S*,15*S*,16*R*,17*S*,18*R*)-17-[tert-butyl(dimethyl)silyl]oxy-1,11-dihydroxy-10,18-dimethoxy-2,14,16-trimethyl-5-phenyl-4,19-dioxabicyclo[13.3.1]nonadecan-3-one (**S5**)

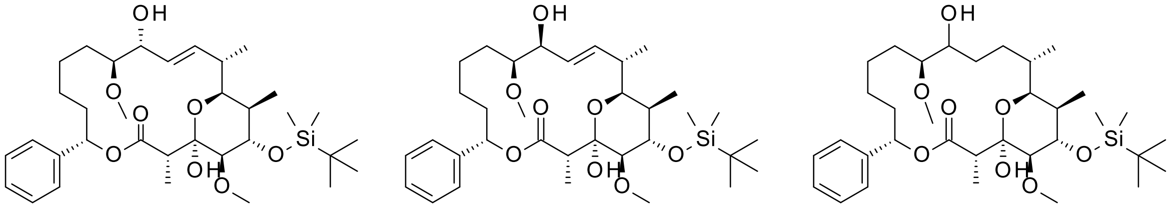

S3 **S4** **S5**

To a solution of **S2** (380 mg, 0.610 mmol) in 1,2-dimethoxyethane (3 mL) was added at 0°C a solution of $ZnCl_2$ (1 M in Et_2O, 9.0 mL, 0.92 mmol) followed by addition of $NaBH_4$ (64.5 mg, 1.53 mmol). The reaction mixture was stirred at 0 °C for 1h40. It was then quenched with sat. aq. NH_4Cl and extracted with EtOAc. The combined organic phases were dried over Na_2SO_4, filtered and concentrated under reduced pressure.
The crude product was purified by flash chromatography on silica gel (gradient: cHex/EtOAc 95:05 to 70:30, 40 mL/min, 22 min) to afford three products: **S3** (186.1 mg, 49%), **S4** (78.4 mg, 21%), and **S5** (23.9 mg, 6%) as a mixture of epimers.

Data for S3:
^{1}H-NMR (400 MHz, $CDCl_3$): δ (ppm) = 7.37–7.22 (m, 5H), 6.33 (dd, *J*=16.1, 4.0 Hz, 1H), 6.07 (dd, *J*=12.1, 2.6 Hz, 1H), 5.39 (ddd, *J*=16.1, 9.4, 1.7 Hz, 1H), 5.17 (d, *J*=1.8 Hz, 1H), 4.24 (dd, *J*=9.2, 2.6 Hz, 1H), 4.14 (t, *J*=2.8 Hz, 1H), 3.64 (dd, *J*=10.6, 2.6 Hz, 1H), 3.44 (s, 3H), 3.36 (s, 3H), 3.34–3.33 (m, 1H), 3.03 (qd, *J*=7.0, 1.5 Hz, 1H), 2.98 (dd, *J*=2.9, 1.1 Hz, 1H), 2.52–2.43 (m, 1H), 2.29 (br m, 1H), 2.13–2.07 (m, 1H), 1.79–1.56 (m, 5H), 1.32–1.24 (m, 2H), 1.21–1.17 (m, 1H), 1.13 (d, *J*=7.3 Hz, 3H), 1.06 (d, *J*=7.7 Hz, 3H), 0.95 (d, *J*=6.6 Hz, 3H), 0.93 (s, 9H), 0.15 (s, 3H), 0.14 (s, 3H);
HPLC-MS: rt = 2.58 min, m/z = 644 $[M+H+Na]^{2+}$; 506 $[M-C_6H_{14}Si]^-$

The relative stereochemistry of **S3** was determined retrospectively from soraphen C after deprotection. The relative stereochemistry of **S4** was determined by comparison with **S3**.

Data for S4:
^{1}H-NMR (400 MHz, $CDCl_3$): δ (ppm) = 7.38–7.24 (m, 5H), 6.12 (ddd, *J*=16.3, 6.1, 1.1 Hz, 1H), 5.90 (dd, *J*=8.6, 5.7 Hz, 1H), 5.55 (ddd, *J*=16.2, 5.8, 1.1 Hz, 1H), 5.08 (d, *J*=1.1 Hz, 1H), 4.29 (t, *J*=5.7 Hz, 1H), 4.11 (t, *J*=2.6 Hz, 1H), 3.84 (dd, *J*=9.9, 2.6 Hz, 1H), 3.43 (s, 3H), 3.35 (s, 3H), 3.35–3.30 (m, 1H), 3.03 (qd, *J*=7.1, 1.1 Hz, 1H), 2.97 (dd, *J*=2.9, 0.7 Hz, 1H), 2.70–2.58 (br s, 1H), 2.53–2.46 (m, 1H), 2.09–2.00 (m, 1H), 1.75–1.62 (m, 5H), 1.56–1.47 (m, 2H), 1.38–1.29 (m, 2H), 1.11 (d, *J*=7.0 Hz, 3H), 1.07 (d, *J*=7.3 Hz, 3H), 0.97 (d, *J*=7.0 Hz, 3H), 0.93 (m, 9H), 0.15 (s, 3H), 0.14 (s, 3H);
^{13}C-NMR (101 MHz, $CDCl_3$): δ (ppm) = 171.91, 141.78, 136.68, 128.43 (2C), 127.65, 126.96, 126.71 (2C), 99.54, 83.58, 77.68, 74.18, 73.18, 71.62, 70.95, 57.69, 57.52, 46.66, 36.69, 36.22, 35.79, 28.91, 25.92 (3C), 24.47, 23.97, 18.23, 15.63, 11.72, 10.74, –4.83, –4.85;
HPLC-MS: rt = 2.66 min, m/z = 644 $[M+H+Na]^{2+}$

Data for S5:
^{1}H-NMR (400 MHz, $CDCl_3$): δ (ppm) = 7.39–7.28 (m, 4H), 7.25–7.21 (m, 1H), 5.98 (dd, *J*=10.8, 3.1 Hz, 1H), 5.15 (d, *J*=1.8 Hz, 1H), 4.15 (t, *J*=2.6 Hz, 1H), 3.88 (dd, *J*=10.6, 2.6 Hz, 1H), 3.74 (dt, *J*=9.1, 3.0 Hz, 1H), 3.42 (s, 3H), 3.39–3.36 (m, 1H), 3.34 (s, 3H), 3.01–2.94 (m, 2H), 2.12–1.98 (m, 3H), 1.93–1.75 (m, 2H), 1.68– 1.56 (m, 6H), 1.53–1.38 (m, 4H), 1.12 (d, *J*=7.0 Hz, 3H), 1.03 (d, *J*=7.3 Hz, 3H), 0.94 (s, 9H), 0.81 (d, *J*=7.0 Hz, 3H), 0.16 (s, 3H), 0.15 (s, 3H);
^{13}C-NMR (101 MHz, $CDCl_3$): δ (ppm) = 171.14, 142.53, 128.43 (2C), 127.58, 126.49 (2C), 99.61, 83.92, 72.81, 72.24, 70.93, 67.36, 57.76, 57.28, 46.52, 36.52, 35.11, 32.06, 28.98, 27.79, 25.94 (3C), 25.15, 23.09, 22.92, 18.25, 14.67, 11.56, 10.45, –4.82 (2C);
HPLC-MS: rt = 2.63 min, m/z = 646 $[M+H+Na]^{2+}$; 508 $[M-C_6H_{14}Si]^-$

(1*R*,2*S*,5*S*,10*S*,11*R*,12*E*,14*S*,15*S*,16*S*,17*S*,18*R*)-1,11,17-trihydroxy-10,18-dimethoxy-2,14,16-trimethyl-5-phenyl-4,19-dioxabicyclo[13.3.1]nonadec-12-en-3-one (soraphen C, **2**)

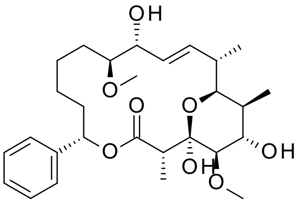

To a solution of **S3** (271 mg, 0.535 mmol) in THF (4 mL) were added at 0°C AcOH (75.8 µL, 1.31 mmol) and TBAF (0.87 mL, 1 M in THF, 0.87 mmol). The reaction mixture was stirred at 0°C for 2 h then at room temperature for 18 h. The reaction mixture was poured into sat. aq. NH_4Cl, then extracted with EtOAc. The combined organic layers were dried over Na_2SO_4, filtered and concentrated under reduced pressure.
The crude product was purified by flash chromatography on silica gel (gradient: cHex/EtOAc 90:10 to 40:60, 35 mL/min, 18 min) to afford the title compound (219 mg, 99%).
Comparison of the spectral data with the ones of the isolated natural product[32,33] confirmed the identity of the product. A table of compared ^{13}C-NMR shifts is presented in Supplementary Table 7.

^{1}H-NMR (400 MHz, $CDCl_3$): δ (ppm) = 7.37–7.28 (m, 5H), 6.15 (dd, *J*=16.0, 3.9 Hz, 1H), 5.82 (dd, *J*=11.2, 3.5 Hz, 1H), 5.48 (ddd, *J*=16.0, 9.4, 1.8 Hz, 1H), 4.36 (s, 1H), 4.17 (td, *J*=9.0, 2.6 Hz, 1H), 4.01 (br d, *J*=8.0 Hz, 1H), 3.81 (dd, *J*=10.4, 2.8 Hz, 1H), 3.62 (d, *J*=9.9 Hz, 1H), 3.43 (s, 3H), 3.38 (s, 3H), 3.37–3.34 (m, 1H), 3.18 (dd, *J*=2.6, 1.1 Hz, 1H), 3.14 (q, *J*=7.3 Hz, 1H), 2.50–2.42 (m, 2H), 2.15–2.07 (m, 1H), 1.93 (q, *J*=7.0 Hz, 1H), 1.82–1.75 (m, 1H), 1.72–1.64 (m, 1H), 1.51–1.45 (m, 2H), 1.38–1.34 (m, 1H), 1.22–1.13 (m, 2H), 1.09 (d, *J*=7.0 Hz, 3H), 1.05 (d, *J*=7.7 Hz, 3H), 1.00 (d, *J*=6.6 Hz, 3H);
^{13}C-NMR (101 MHz, $CDCl_3$): δ (ppm) = 170.78, 141.12, 137.45, 128.72 (2C), 128.29, 126.35 (2C), 125.18, 99.60, 83.89, 76.28, 75.04, 74.81, 72.66, 68.98, 57.78, 57.47, 46.35, 36.01, 35.79, 35.32, 29.57, 26.14, 23.17, 12.64, 11.83, 10.49;
HPLC-MS: rt = 1.65 min, m/z = 505 $[M-H]^-$
HR-MS: m/z calculated for $C_{28}H_{43}O_8$ $[(M+H)^+]$: 507.2952, found 507.2962.

(1*R*,2*S*,5*S*,10*S*,11*S*,12*E*,14*S*,15*S*,16*S*,17*S*,18*R*)-1,11,17-trihydroxy-10,18-dimethoxy-2,14,16-trimethyl-5-phenyl-4,19-dioxabicyclo[13.3.1]nonadec-12-en-3-one (*epi*-soraphen C, **S7**)

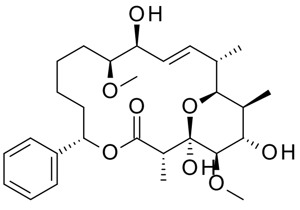

To a solution of **S4** (10 mg, 0.016 mmol) in THF (0.4 mL) were added at 0°C AcOH (2.8 µL, 0.048 mmol) and TBAF (0.03 mL, 1 M in THF, 0.32 mmol). The reaction mixture was stirred at 0°C for 2 h then at room temperature for 18 h. The reaction mixture was poured into sat. aq. NH_4Cl, then extracted with a mixture of DCM/MeOH (9:1). The combined organic layers were dried over Na_2SO_4, filtered and concentrated under reduced pressure.
The crude product was purified by flash chromatography on silica gel (gradient: cHex/EtOAc 70:30 to 50:50, 18 mL/min, 9 min) to afford the title compound (5 mg, 61%) containing some impurities.

^{1}H-NMR (600 MHz, $CDCl_3$): δ (ppm) = 7.37–7.29 (m, 5H), 5.89 (dd, *J*=15.9, 6.3 Hz, 1H), 5.67 (t, *J*=7.3 Hz, 1H), 5.53 (ddd, *J*=15.8, 7.1, 1.1 Hz, 1H), 4.69 (s, 1H), 4.20 (t, *J*=6.6 Hz, 1H), 3.99 (dd, *J*=10.4, 2.5 Hz, 1H), 3.98 (br. s, 1H), 3.69 (br. s, 1H), 3.42 (s, 3H), 3.37 (s, 3H), 3.30–3.26 (m, 1H), 3.14 (dd, *J*=2.5, 0.9 Hz, 1H), 3.11 (q, *J*=7.2 Hz, 1H), 2.53 (br s, 1H), 2.47–2.43 (m, 1H), 2.24–2.18 (m, 1H), 1.94–1.90 (m, 1H), 1.85–1.78 (m, 2H), 1.66–1.61 (m, 1H), 1.56–1.51 (m, 1H), 1.46–1.38 (m, 3H), 1.04 (d, *J*=7.5 Hz, 3H), 1.03 (d, *J*=7.1 Hz, 3H), 0.99 (d, *J*=6.6 Hz, 3H);
^{13}C-NMR (151 MHz, $CDCl_3$): δ (ppm) = 172.82, 139.69, 137.96, 128.76 (2C), 128.70, 128.46 (2C), 127.09, 99.91, 82.86, 77.57, 76.33, 73.58, 71.74, 68.93, 57.49, 57.45, 45.42, 36.84, 35.46, 35.02, 27.58, 25.12, 22.57, 16.57, 12.55, 10.55;
HPLC-MS: rt = 1.77 min, m/z = 505 $[M-H]^-$

(1*S*,2*R*,3*R*,5*S*,6*S*,7*S*,12*S*,15*S*,16*R*,17*R*,18*S*,19*R*)-18-[tert-butyl(dimethyl)silyl]oxy-16-hydroxy-6,7,17-trimethoxy-2,15,19-trimethyl-12-phenyl-4,13,20-trioxatricyclo[14.3.1.03,5]icosan-14-one (**S6**)

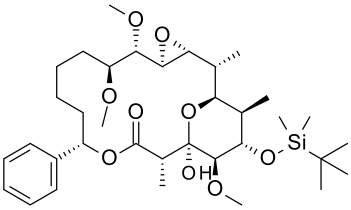

To a solution of **S3** (400 mg, 0.630 mmol) in DCM (6.3 mL) at rt was added mCPBA (706 mg, 3.150 mmol). The reaction mixture was stirred for 17 h. The reaction mixture was poured into sat. aq. $NaHCO_3$. The phases were separated, the aqueous layer was extracted with EtOAc. The combined organic layers were washed with brine, dried over Na_2SO_4, filtered and concentrated under reduced pressure.
The crude product was purified by flash chromatography on silica gel (gradient: cHex/EtOAc 100:0 to 80:20, 40 mL/min, 18min) to afford the title compound (330 mg, 80%).

^{1}H-NMR (400 MHz, $CDCl_3$): δ (ppm) = 7.37–7.32 (m, 4H), 7.26–7.23 (m, 1H), 6.06 (dd, *J*=12.3, 2.8 Hz, 1H), 5.43 (d, *J*=1.8 Hz, 1H), 4.18 (t, *J*=1.8 Hz, 1H), 4.00 (dd, *J*=11.0, 2.6 Hz, 1H), 3.55 (s, 3H), 3.49 (t, *J*=2.0 Hz, 1H), 3.41 (s, 3H), 3.38 (s, 3H), 3.37–3.35 (m, 1H), 3.16 (dd, *J*=8.1, 2.2 Hz, 1H), 3.06–3.01 (m, 2H), 2.95 (dd, *J*=8.3, 1.7 Hz, 1H), 2.37–2.29 (m, 1H), 2.06–1.97 (m, 2H), 1.72–1.66 (m, 1H), 1.64–1.55 (m, 2H), 1.47–1.44 (m, 1H), 1.40–1.28 (m, 2H), 1.18 (d, *J*=7.3 Hz, 3H), 1.08 (d, *J*=7.3 Hz, 3H), 1.04–0.97 (m, 1H), 0.94 (s, 9H), 0.60 (d, *J*=7.0 Hz, 3H), 0.17 (s, 3H), 0.15 (s, 3H);
^{13}C-NMR (101 MHz, $CDCl_3$): δ (ppm) = 170.83, 142.61, 128.48 (2C), 127.55, 126.20 (2C), 99.68, 83.66, 83.23, 71.87, 70.52, 68.14, 58.58, 58.07, 57.39, 55.13, 53.63, 46.39, 36.89, 35.49, 34.05, 30.39, 27.06, 25.80 (3C), 24.83, 24.18, 18.05, 11.26, 10.09, 8.92, –4.86, –4.99;
HPLC-MS: rt = 2.70 min, m/z = 674 $[M+Na]^+$
HR-MS: m/z calculated for $C_{35}H_{58}NaO_9Si$ [$(M+Na)^+$]: 673.3742, found 673.3727.

(1*R*,2*R*,3*R*,5*S*,6*S*,7*S*,12*S*,15*S*,16*R*,17*R*,18*S*,19*S*)-16,18-dihydroxy-6,7,17-trimethoxy-2,15,19-trimethyl-12-phenyl-4,13,20-trioxatricyclo[14.3.1.03,5]icosan-14-one (**4**)

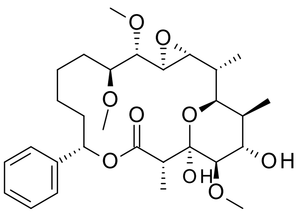

To a solution of **S6** (660 mg, 1.01 mmol) in THF (10 mL) were added at 0°C AcOH (176 µL, 3.04 mmol) and TBAF (2.00 mL, 1 M in THF, 2.03 mmol). The reaction mixture was stirred at 0°C for 2.5 h. The reaction mixture was poured into sat. aq. NH_4Cl, then extracted with EtOAc. The combined organic layers were dried over Na_2SO_4, filtered and concentrated under reduced pressure.

The crude product was purified by flash chromatography on silica gel (gradient: cHex/EtOAc 85:15 to 60:40, 18 mL/min, 11 min) to afford the title compound (500 mg, 92%).

^{1}H-NMR (400 MHz, $CDCl_3$): δ (ppm) = 7.35–7.24 (m, 5H), 5.86 (dd, *J*=12.3, 1.8 Hz, 1H), 4.47 (s, 1H), 4.11–4.08 (m, 2H), 3.59 (d, *J*=8.0 Hz, 1H), 3.54 (s, 3H), 3.43 (s, 3H), 3.40–3.38 (m, 1H), 3.37 (s, 3H), 3.30 (t, *J*=2.0 Hz, 1H), 3.17 (d, *J*=1.8 Hz, 1H), 3.13–3.07 (m, 2H), 2.97 (dd, *J*=8.4, 3.6 Hz, 1H), 2.32–2.25 (m, 1H), 2.10–2.04 (m, 1H), 1.87–1.81 (m, 1H), 1.73–1.62 (m, 3H), 1.55–1.44 (m, 3H), 1.25–1.20 (m, 1H), 1.14 (d, *J*=6.9 Hz, 3H), 1.05 (d, *J*=7.6 Hz, 3H), 0.64 (d, *J*=6.9 Hz, 3H);

^{13}C-NMR (101 MHz, $CDCl_3$): δ (ppm) = 170.72, 141.64, 128.59 (2C), 127.99, 126.14 (2C), 99.78, 82.98, 76.34, 74.08, 68.96, 68.87, 58.75, 58.40, 57.35, 55.78, 54.43, 53.53, 46.67, 36.47, 35.40, 34.02, 29.47, 25.78, 23.73, 11.58, 10.25, 9.01;

HPLC-MS: rt = 1.86 min, m/z = 535 [M-H]$^{-}$

HR-MS: m/z calculated for $C_{29}H_{44}NaO_9$ [$(M+Na)^+$]: 559.2878, found 559.2882.

Purification of bio-extracts

Supplementary Table 8. Structures of isolated modified soraphen compounds.

Starting material	Structure of isolated compounds		
Soraphen A	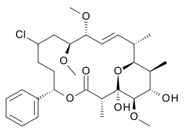<br>**1a**	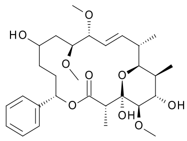<br>**1c**	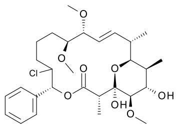<br>**1b**
Soraphen C	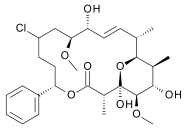<br>**2a**		

All bioextracts were purified by preparative reverse-phase HPLC. In each case, the bioextract was dissolved in 1 mL of DMSO and injected on a Fraction Lynx Prep HPLC equipped with a Column Hichrom C18 ODS-2 5 250 mm x 2.1mm i.d. The mobile phase consisted of a mixture of H_2O+0.1% HCOOH and ACN+0.1% HCOOH. The flow was set at 20 mL min^{-1}. The gradients used for the purification of the different extracts are presented below.

Supplementary Table 9. Prep. HPLC gradient for **1a** and **1c**.

Time (min)	% H_2O + 0.1% HCOOH	% ACN + 0.1% HCOOH
0	60	40
2	60	40
37	0	100
40	1	100
41	50	50
45	50	50

(1a) rt = 23.6 min; **(1c)** rt = 11.7 min;

Supplementary Table 10. Prep. HPLC gradient for **1b**.

**First column:**			**Second column:**		
Time (min)	% H_2O + 0.1% HCOOH	% ACN + 0.1% HCOOH	Time (min)	% H_2O + 0.1% HCOOH	% ACN + 0.1% HCOOH
0	60	40	0	50	50
2	60	40	2	50	50
35	0	100	35	0	100
40	0	100	40	0	100
41	60	40	41	50	50
45	60	40	45	50	50

(1b) rt (second column)= 20.6 min.

Supplementary Table 11. Prep. HPLC gradient for **2a**.

**Time (min)**	**% H_2O + 0.1% HCOOH**	**% ACN + 0.1% HCOOH**
0	60	40
2	60	40
37	0	100
40	0	100
41	60	40
45	60	40

Analytical data of the halogenated and hydroxylated products

(1*R*,2*S*,5*S*,10*S*,11*R*,12*E*,14*S*,15*S*,16*S*,17*S*,18*R*)-8-chloro-1,17-dihydroxy-10,11,18-trimethoxy-2,14,16-trimethyl-5-phenyl-4,19-dioxabicyclo[13.3.1]nonadec-12-en-3-one (**1a**)

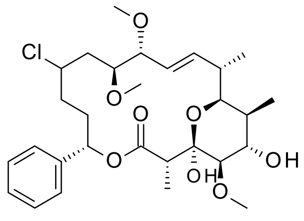

Isolated yield: 10% (11.0 mg)
^{1}H-NMR (600 MHz, CDCl$_3$) δ (ppm) = 7.36–7.34 (m, 4H), 7.30 (dq, *J*=8.5, 4.3 Hz, 1H), 6.21 (dd, *J*=16.3, 3.7 Hz, 1H), 5.92 (dd, *J*=11.8, 2.5 Hz, 1H), 5.49 (ddd, *J*=16.2, 9.5, 1.7 Hz, 1H), 4.57 (s, 1H), 4.12–4.06 (m, 1H), 4.00 (tt, *J*=11.7, 2.5 Hz, 1H), 3.88 (dt, *J*=10.7, 2.7 Hz, 1H), 3.77 (dd, *J*=10.5, 2.5 Hz, 1H), 3.68 (dd, *J*=9.5, 1.9 Hz, 1H), 3.48 (s, 3H), 3.38 (s, 3H), 3.32 (s, 3H), 3.19-3.15 (m, 2H), 3.12 (q, *J*=7.0 Hz, 1H), 2.53–2.49 (m, 1H), 2.21–2.15 (m, 1H), 2.06–2.02 (m, 1H), 2.01–1.96 (m, 1H), 1.92–1.89 (m, 1H), 1.88–1.85 (m, 1H), 1.77–1.73 (m, 1H), 1.69 (ddd, *J*=13.9, 11.5, 2.1 Hz, 1H), 1.56 (br s, 1H), 1.13 (d, *J*=7.1 Hz, 3H), 1.07 (d, *J*=7.6 Hz, 3H), 1.02 (d, *J*=6.7 Hz, 3H);
^{13}C-NMR (151 MHz, CDCl$_3$) δ = 170.97, 141.00, 140.12, 128.75 (2C), 128.30, 126.15 (2C), 122.97, 99.54, 84.03, 79.98, 76.33, 73.15, 72.40, 69.22, 58.48, 57.47, 56.89, 56.51, 46.49, 39.67, 35.85, 35.42, 34.33, 32.90, 12.58, 11.71, 10.43 ppm;
HPLC-MS: rt = 2.07 min, m/z = 578/580 [M+H+Na]$^{2+}$, 553/555 [M–H]$^{-}$ chloro isotopic pattern

(1*R*,2*S*,5*S*,10*S*,11*R*,12*E*,14*S*,15*S*,16*S*,17*S*,18*R*)-1,8,17-trihydroxy-10,11,18-trimethoxy-2,14,16-trimethyl-5-phenyl-4,19-dioxabicyclo[13.3.1]nonadec-12-en-3-one (**1c**)

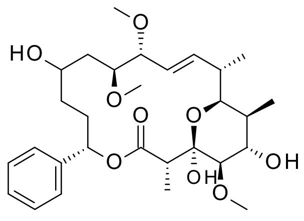

Isolated yield: 3% (3.7 mg)
^{1}H-NMR (600 MHz, CDCl$_3$) δ = 7.36–7.33 (m, 4H), 7.30 (dq, *J*=8.7, 4.2 Hz, 1H), 6.16 (dd, *J*=16.3, 3.7 Hz, 1H), 5.87 (dd, *J*=10.2, 4.7 Hz, 1H), 5.49 (ddd, *J*=16.2, 9.3, 1.8 Hz, 1H), 4.48 (s, 1H), 4.05 (br d, *J*=8.7 Hz, 1H), 3.86 (br s, 1H), 3.81 (br dd, *J*=10.5, 2.5 Hz, 1H), 3.76–3.73 (m, 1 H), 3.72 (dd, *J*=9.3, 1.8 Hz, 1H), 3.47 (s, 3H), 3.42 (br d, *J*=9.3 Hz, 1H), 3.38 (s, 3H), 3.30 (s, 3H), 3.18 (d, *J*=1.6 Hz, 1H), 3.13 (q, *J*=7.0 Hz, 1H), 2.51 (m, 1H), 2.17 (s, 1H), 2.05–1.96 (m, 2H), 1.97–1.91 (m, 1H), 1.69 (ddd, *J*=14.0, 10.4, 1H), 1.55–1.48 (m, 5H), 1.11 (d, *J*=7.1 Hz, 3H), 1.06 (d, *J*=7.5 Hz, 3H), 1.03 (d, *J*=6.7 Hz, 3H) ppm
^{13}C-NMR (151 MHz, CDCl$_3$) δ = 170.93, 140.83, 140.18, 128.75 (2C), 128.37, 126.41 (2C), 122.99, 99.60, 84.83, 80.05, 76.30, 74.32, 72.39, 69.11, 65.78, 58.36, 57.49, 56.44, 46.38, 38.78, 35.80, 35.46, 33.53, 31.78, 31.08, 12.69, 11.71, 10.51 ppm
MS: m/z = 559 [M+Na]$^{+}$

(2*S*,5*R*,10*S*,11*R*,12*E*,14*S*,15*S*,16*S*,17*S*,18*R*)-6-chloro-1,17-dihydroxy-10,11,18-trimethoxy-2,14,16-trimethyl-5-phenyl-4,19-dioxabicyclo[13.3.1]nonadec-12-en-3-one (**1b**)

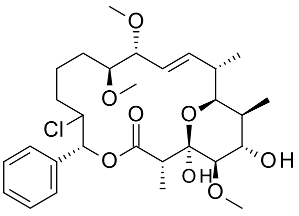

Isolated yield: 4% (4.1 mg)

^{1}H-NMR (600 MHz, $CDCl_3$) δ = 7.41–7.35 (m, 4H), 7.35–7.32 (m, 1H), 6.22 (dd, *J*=16.2, 3.9 Hz, 1H), 5.88 (d, *J*=9.1Hz, 1H), 5.48 (ddd, *J*=16.2, 9.5, 1.8 Hz, 1H), 4.39 (s, 1H), 4.38–4.35 (m, 1H), 4.12–4.06 (m, 1H), 3.79 (dd, *J*=10.5, 2.7 Hz, 1H), 3.63 (dd, *J*=9.5, 2.1 Hz, 1H), 3.47–3.43 (m, 4H), 3.37 (s, 3H), 3.30 (s, 3H), 3.16–3.14 (m, 1H), 3.14–3.09 (m, 2H), 2.56–2.46 (m, 1H), 2.17 (s, 1H) 2.03 (qd, *J*=9.9, 4.5 Hz, 1H) 1.90 (q, *J*=7.5 Hz, 1H) 1.87–1.80 (m, 1H), 1.66–1.61 (m, 2H), 1.45–1.36 (m, 1H), 1.35–1.25 (m, 2H), 1.07 (d, *J*=6.3 Hz, 3H), 1.06 (d, *J*=6.0 Hz, 3H), 1.02 (d, *J*=6.8 Hz, 3H) ppm;

^{13}C-NMR (151 MHz, $CDCl_3$) δ = 170.01, 140.07, 137.28, 128.99, 128.40 (2C), 128.05 (2C), 122.64, 99.66, 84.90, 83.23, 76.26, 75.65, 72.68, 69.27, 62.15, 58.25, 57.52, 56.39, 46.37, 35.82, 35.30, 33.83, 29.53, 18.98, 12.61, 11.55, 10.39 ppm.

HPLC-MS: rt = 1.80 min, m/z = 564/566 $[M+H+Na]^{2+}$, 539/541 $[M–H]^-$ chloro isotopic pattern

(1*R*,2*S*,5*S*,10*S*,11*R*,12*E*,14*S*,15*S*,16*S*,17*S*,18*R*)-8-chloro-1,11,17-trihydroxy-10,18-dimethoxy-2,14,16-trimethyl-5-phenyl-4,19-dioxabicyclo[13.3.1]nonadec-12-en-3-one (**2a**)

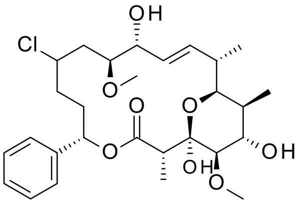

Isolated yield: 10% (16.1 mg)

^{1}H-NMR (600 MHz, $CDCl_3$) δ = 7.38–7.33 (m, 4H), 7.33–7.28 (m, 1H), 6.16 (dd, *J*=16.1, 3.8 Hz, 1H), 5.89 (dd, *J*=11.7, 2.7 Hz, 1H), 5.50 (ddd, *J*=16.1, 9.4, 1.8 Hz, 1H), 4.58 (s, 1H), 4.19 (dd, *J*=9.3, 2.1 Hz, 1H), 4.08 (br s, 1H), 4.00 (tt, *J*=11.5, 2.7 Hz, 1H), 3.81 (dt, *J*=11.0, 2.9 Hz, 1H), 3.76 (dd, *J*=10.6, 2.6 Hz, 1H), 3.46 (s, 3H), 3.38 (s, 3H), 3.30 (br d, *J*=7.3 Hz, 1H), 3.17 (d, *J*=1.8 Hz, 1H), 3.12 (q, *J*=7.2 Hz, 1H), 2.51–2.43 (m, 1H), 2.39–2.29 (m, 1H), 2.17 (ddt, *J*=15.0, 11.6, 3.5, 3.5 Hz, 1H), 2.08–2.01 (m, 2H), 1.93–1.88 (m, 1H), 1.84 (ddt, *J*=14.8, 11.8, 3.4, 3.4 Hz, 1H), 1.75 (ddt, *J*=14.9, 11.2, 4.0, 4.0 Hz, 1H), 1.69–1.64 (m, 2H), 1.11 (d, *J*=7.0 Hz, 3H), 1.06 (d, *J*=7.5 Hz, 3H), 1.00 (d, *J*=6.8 Hz, 3H) ppm

^{13}C-NMR (151 MHz, $CDCl_3$) δ = 170.83, 140.85, 137.99, 128.77 (2C), 128.34, 126.17 (2C), 125.19, 99.54, 81.27, 76.29, 74.54, 73.31, 72.42, 69.18, 58.09, 57.47, 56.58, 46.39, 39.15, 35.86, 35.35, 34.29, 32.84, 12.60, 11.71, 10.47 ppm

MS: m/z = 563/565 $[M+Na]^+$ chloro isotopic pattern

Supplementary Table 12. Assignment Table ^{1}H-NMR.

Chemical shifts (in ppm) for multiplets were taken at the center of the multiplet for clarity.
The assignment of the peaks was done based on analysis of 2D spectra: COSY, HSQC, HMBC, ROESY.

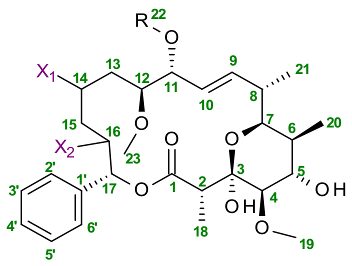

Carbon number	Soraphen A	(1a) R=Me; X_1=Cl, X_2=H	(1b) R=Me; X_1=H, X_2=Cl	(1c) R=Me; X_1=OH, X_2=H	Soraphen C R=H; X_1=H, X_2=H	(2a) R=H; X_1=Cl, X_2=H
**2**	3.14	3.15	3.12	3.16	3.15	3.12
**4**	3.18	3.19	3.15	3.20	3.18	3.17
**5**	4.02	4.12	4.09	4.07	4.02	4.00
**6**	1.94	1.95	1.90	1.95	1.93	1.90
**7**	3.83	3.79	3.79	3.84	3.82	3.76
**8**	2.49	2.54	2.51	2.54	2.45	2.47
**9**	6.17	6.23	6.22	6.19	6.15	6.16
**10**	5.48	5.51	5.48	5.51	5.49	5.50
**11**	3.68	3.71	3.63	3.75	4.17	4.19
**12**	3.41	3.91	3.45	3.77	3.36	3.81
**13**	1.24<br>1.69	1.72<br>2.01	1.34<br>1.64	1.52<br>1.71	1.17<br>1.77	1.66<br>2.05
**14**	1.16<br>1.46	4.02	1.43<br>1.64	3.86	1.17<br>1.51	4.00
**15**	1.34<br>1.46	1.77<br>1.89	1.86<br>2.03	1.58<br>1.58	1.35<br>1.48	1.75<br>1.85
**16**	1.67<br>2.10	2.07<br>2.20	4.37	2.02<br>2.05	1.67<br>2.10	2.05<br>2.18
**17**	5.84	5.95	5.88	5.90	5.82	5.89
**18**	1.11	1.15	1.07	1.14	1.09	1.11
**19**	3.38	3.41	3.37	3.41	3.38	3.38
**20**	1.05	1.10	1.06	1.19	1.05	1.06
**21**	1.03	1.05	1.02	1.05	1.00	1.00
**22**	3.29	3.35	3.30	3.33	/	/
**23**	3.44	3.50	3.45	3.49	3.43	3.46
**2’**	7.32	7.38	7.38	7.37	7.35	7.35
**3’**	7.32	7.38	7.38	7.38	7.35	7.35
**4’**	7.32	7.32	7.34	7.33	7.27	7.30
**5’**	7.32	7.38	7.38	7.38	7.35	7.35
**6’**	7.32	7.38	7.38	7.37	7.35	7.35

NMR spectra

Supplementary Figure 16. NMR of compound **1**.

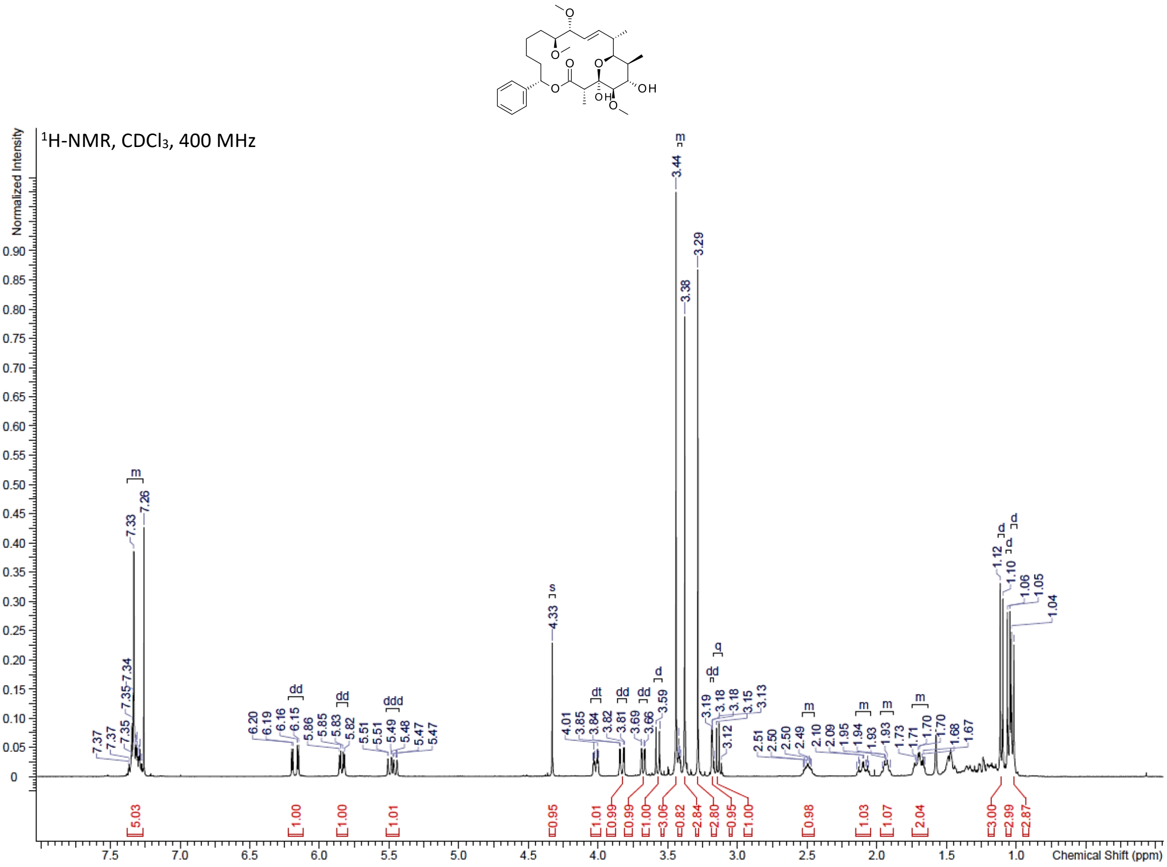

Supplementary Figure 17. NMR of compound **S1**.

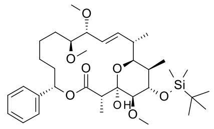

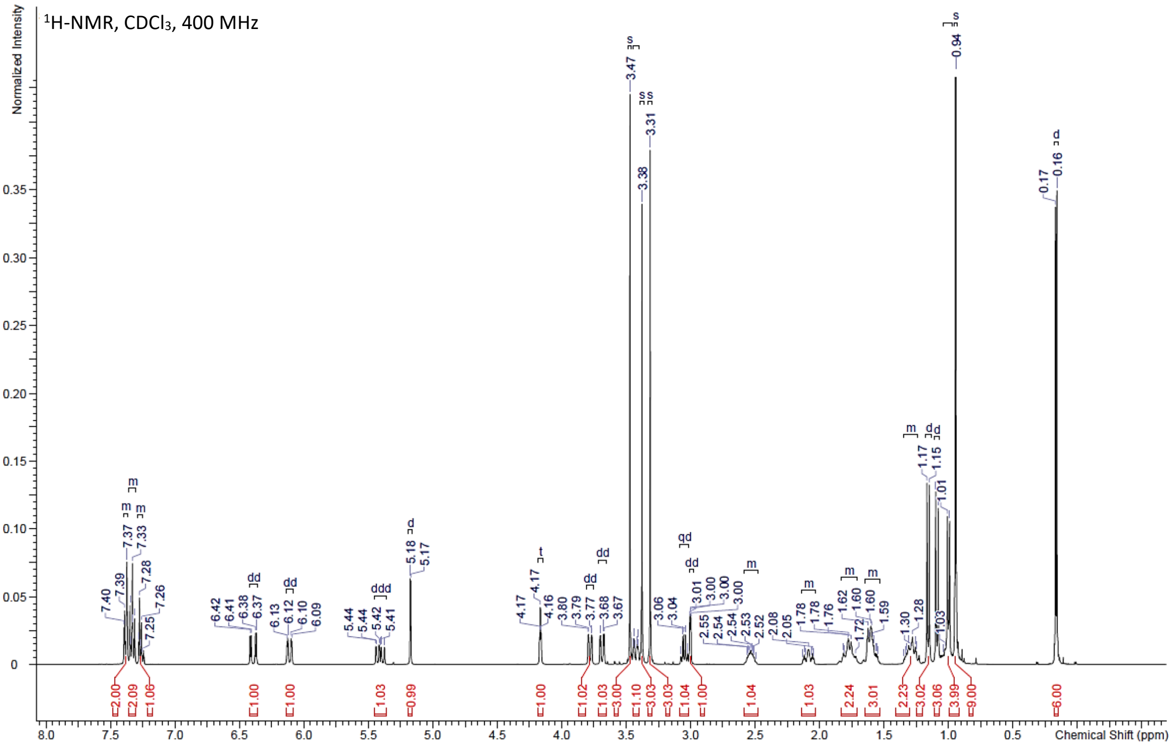

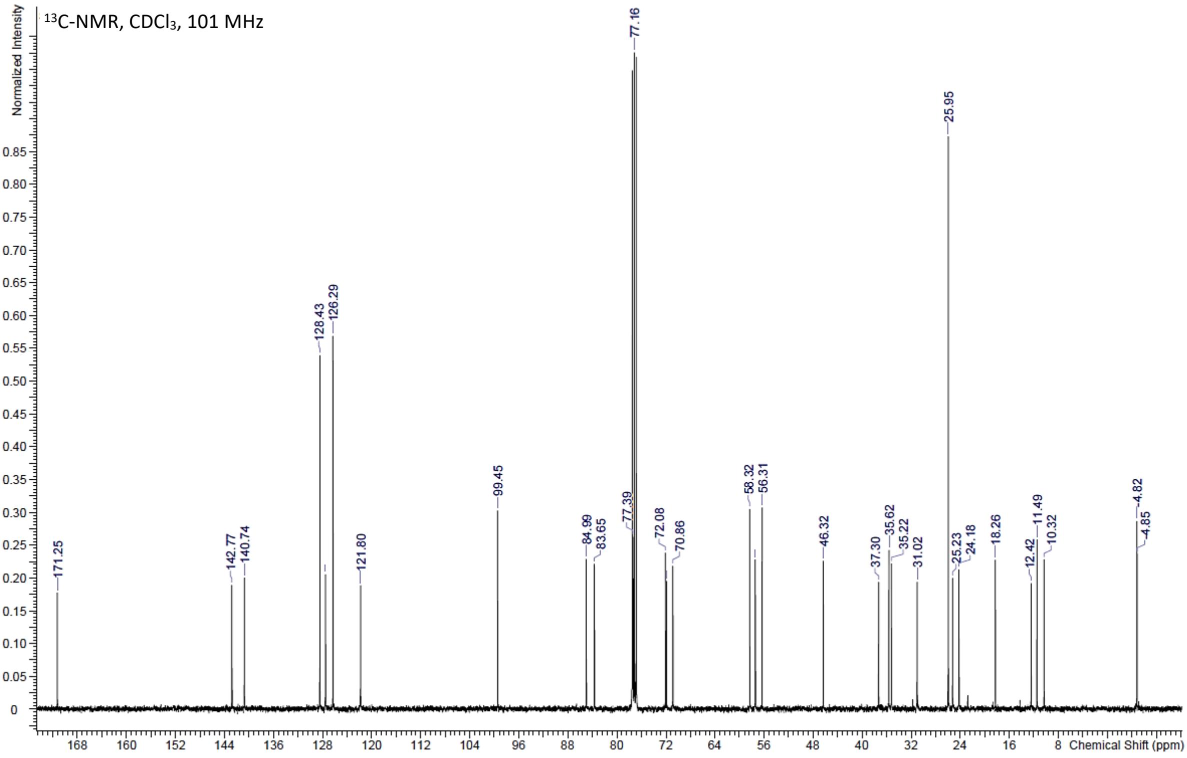

Supplementary Figure 18. NMR of compound **S2**.

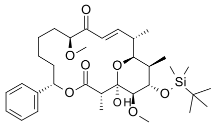

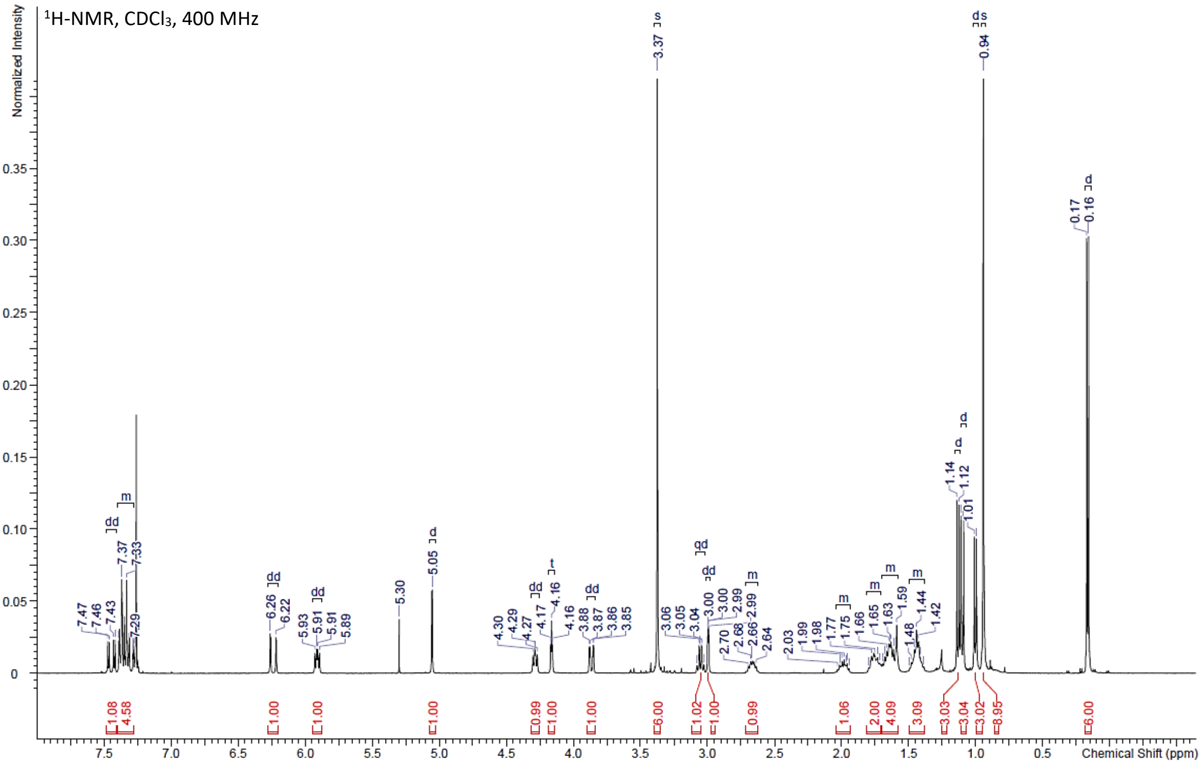

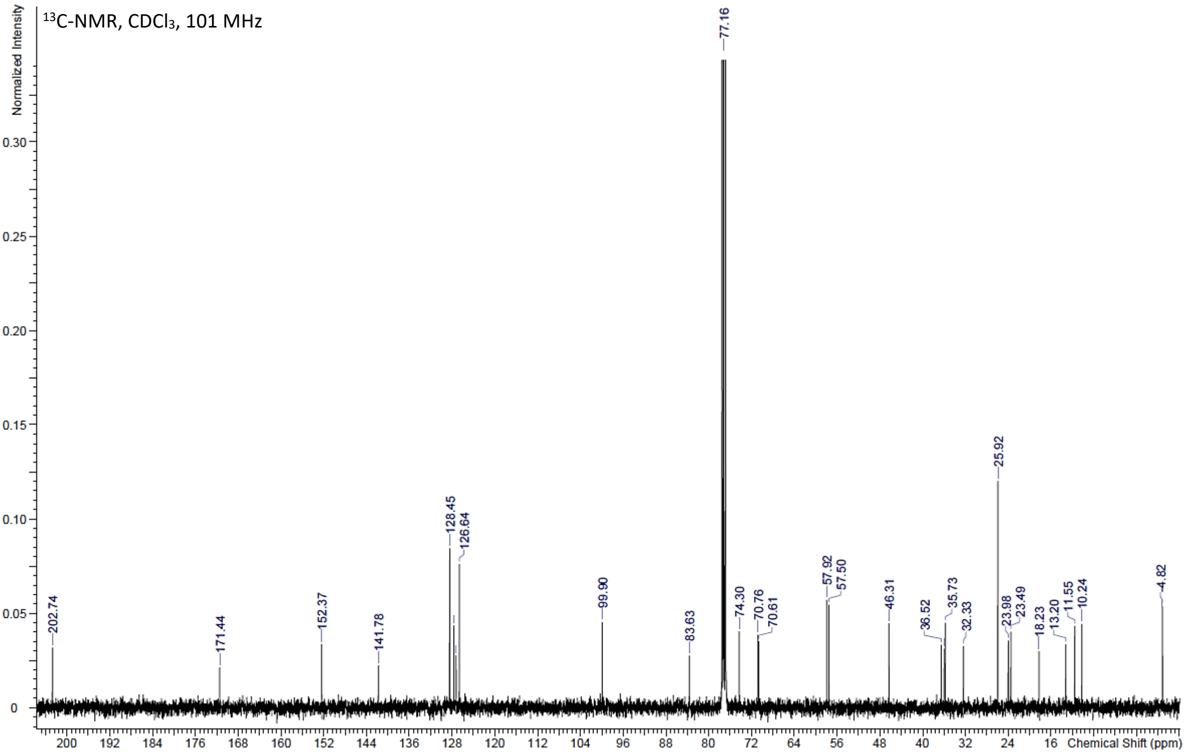

Supplementary Figure 19. NMR of compound **3**.

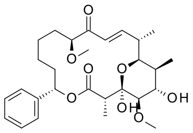

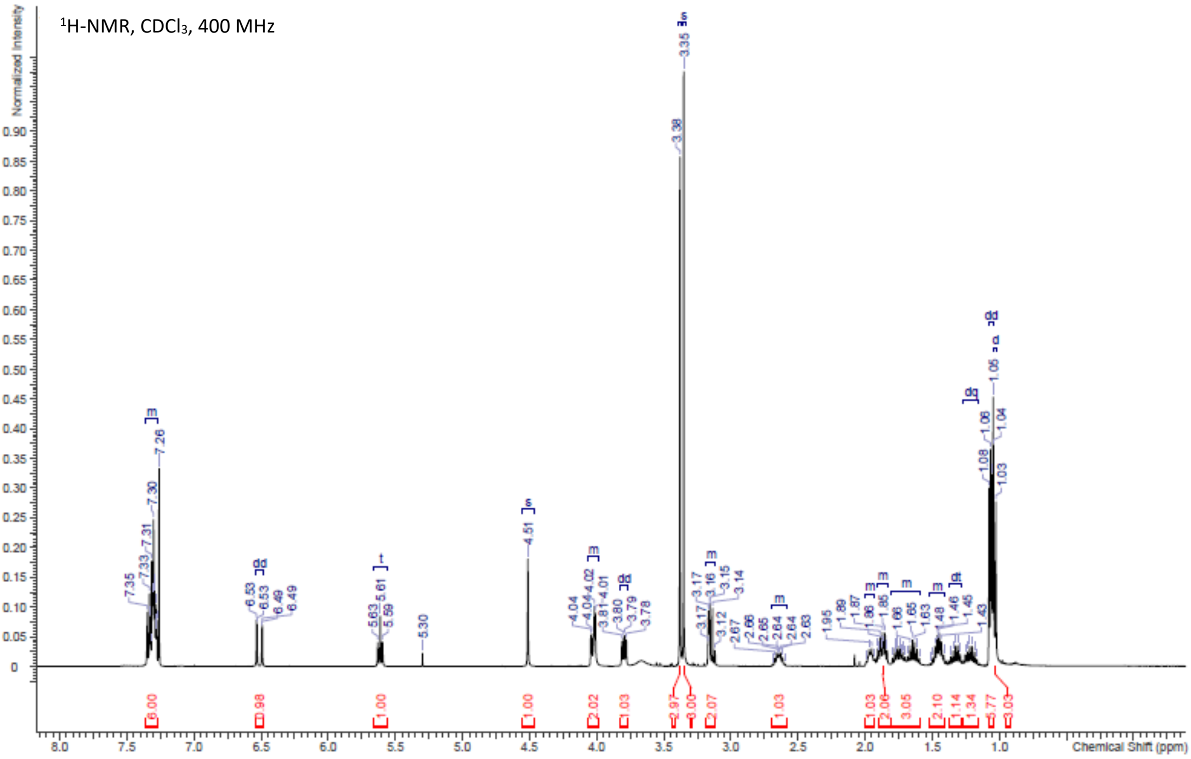

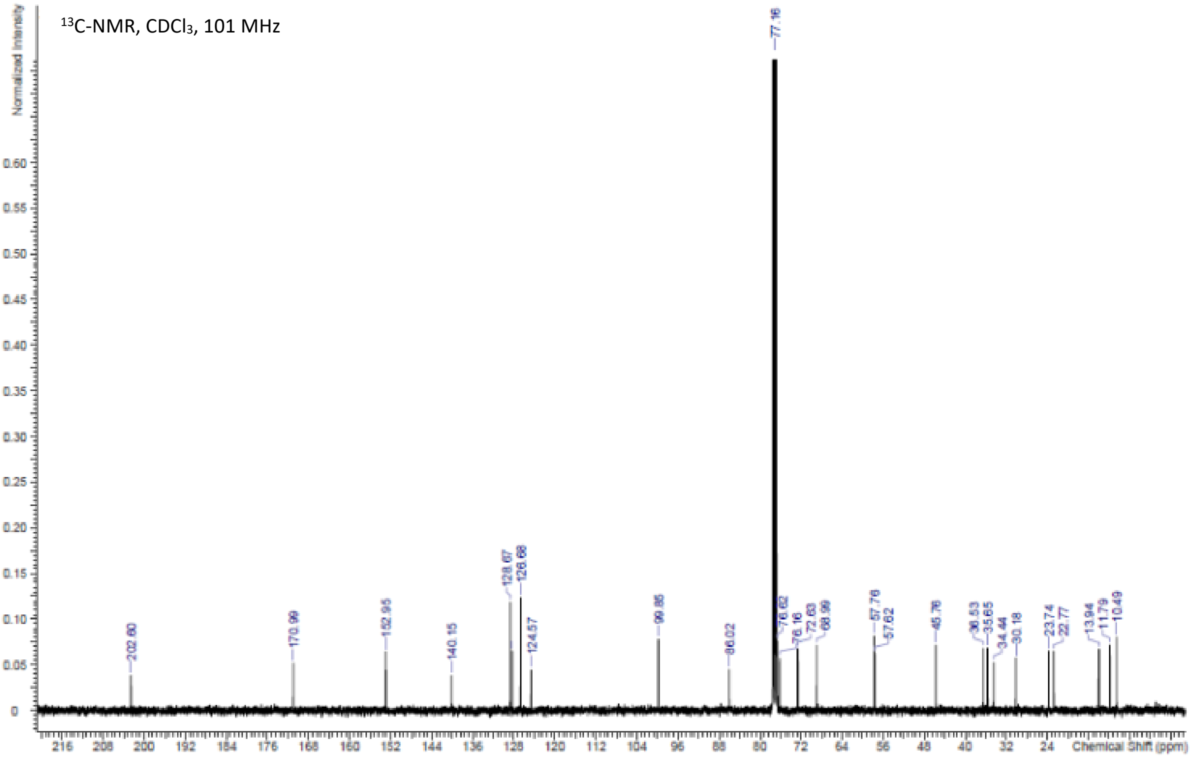

Supplementary Figure 20. NMR of compound **S3**.

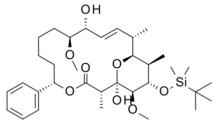

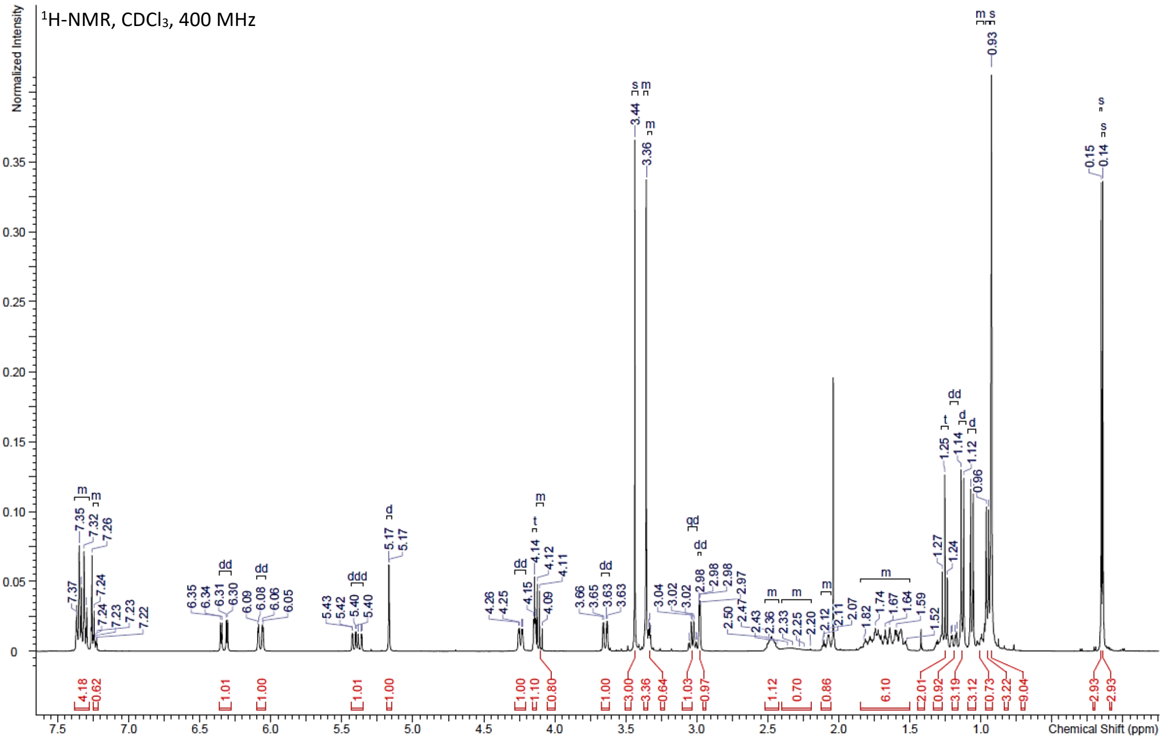

Supplementary Figure 21. NMR of compound **S4**.

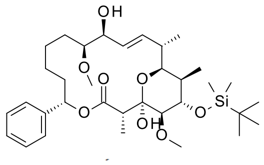

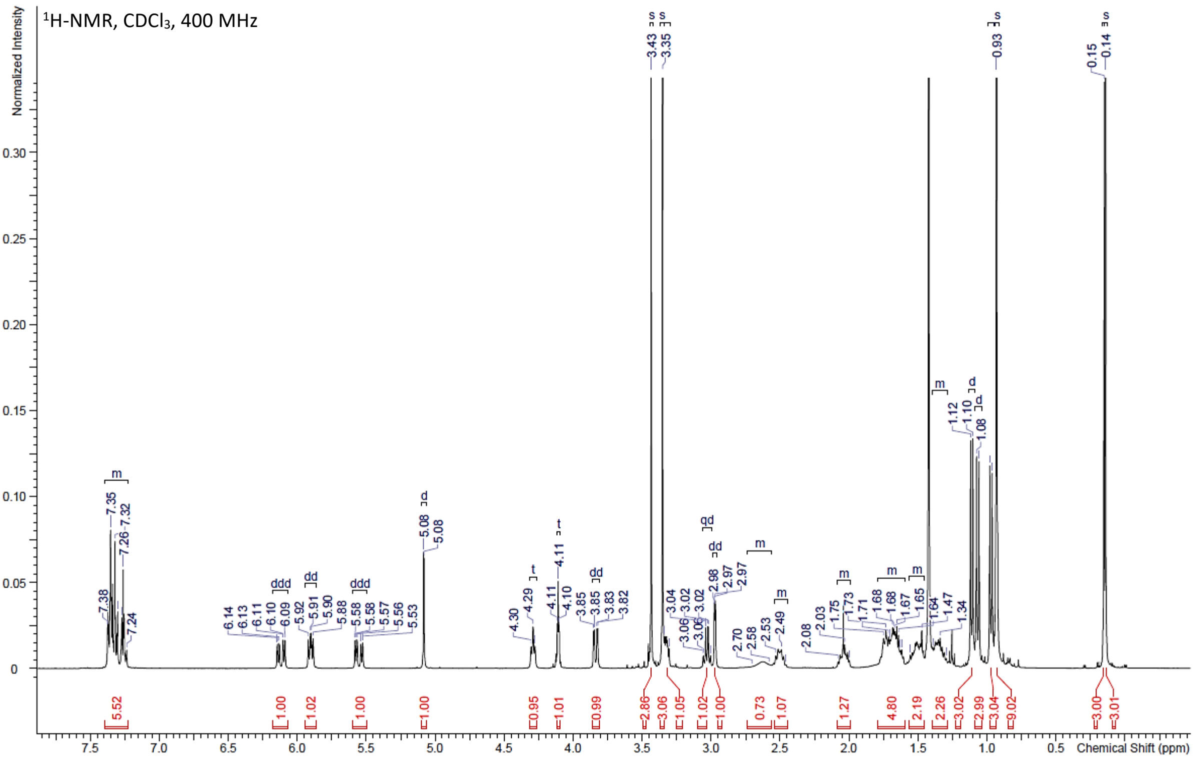

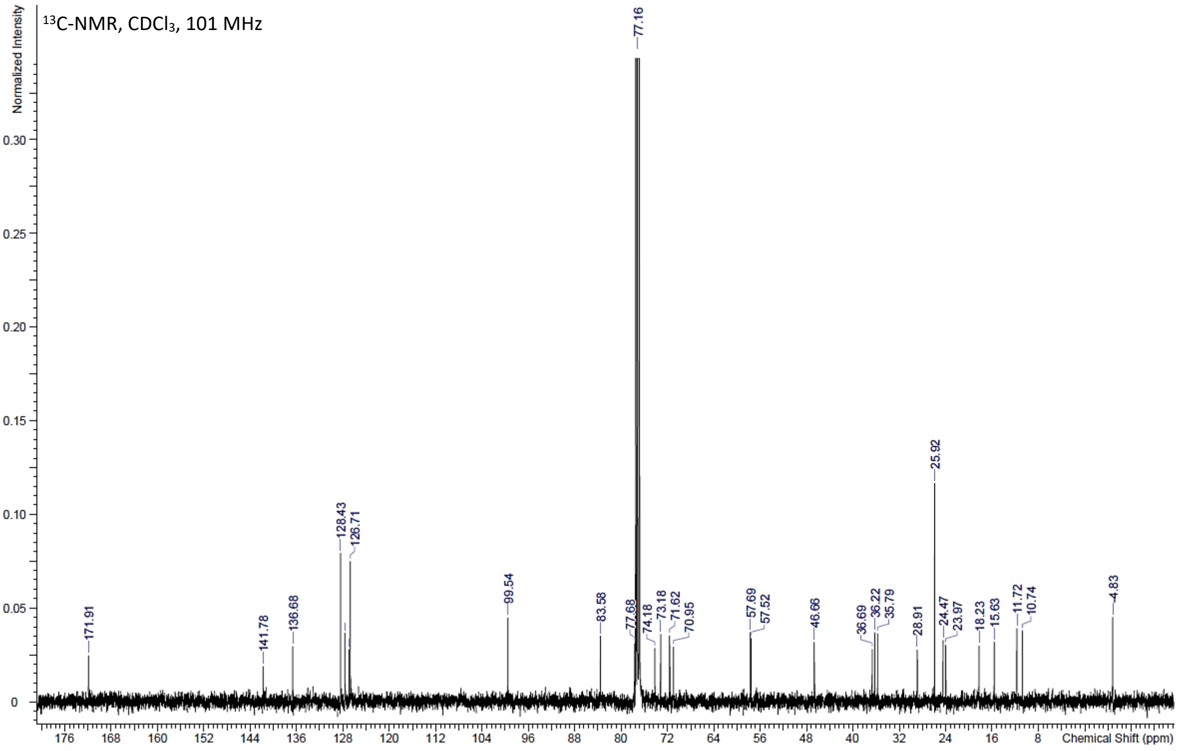

Supplementary Figure 22. NMR of compound **S5**.

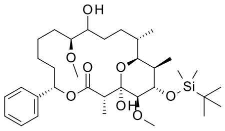

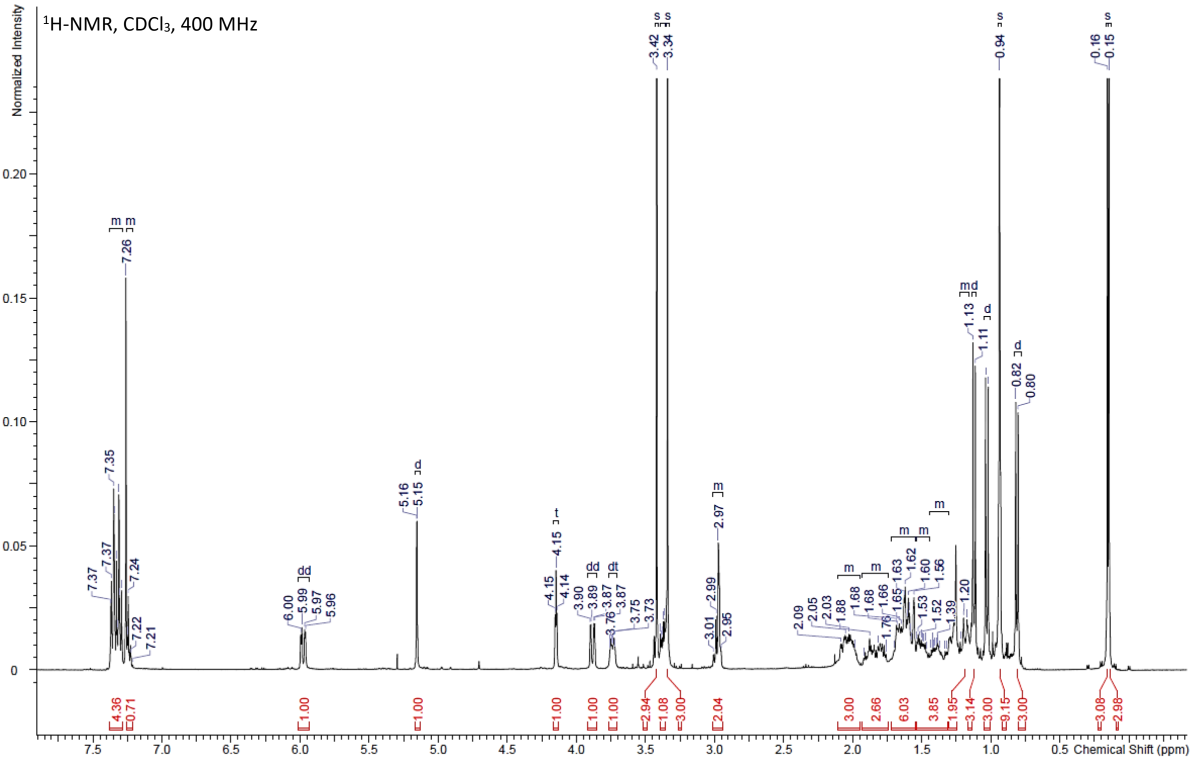

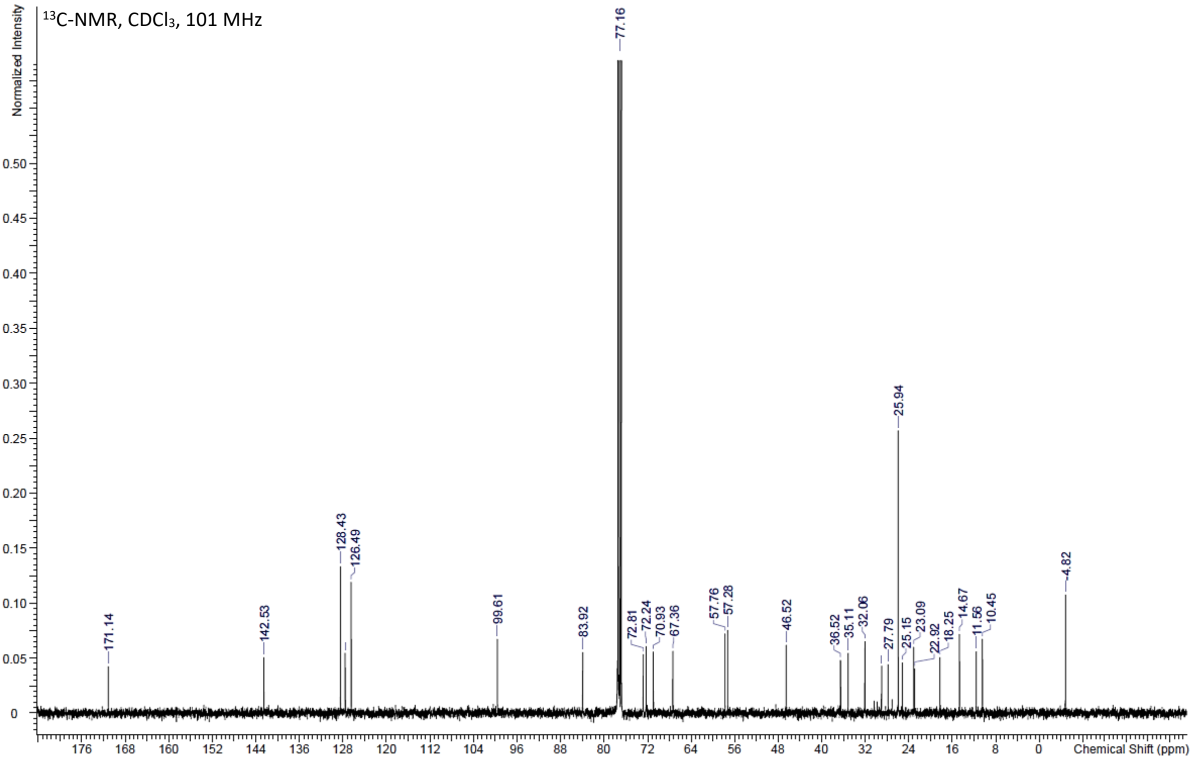

Supplementary Figure 23. NMR of compound soraphen C, **2**.

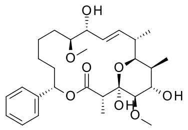

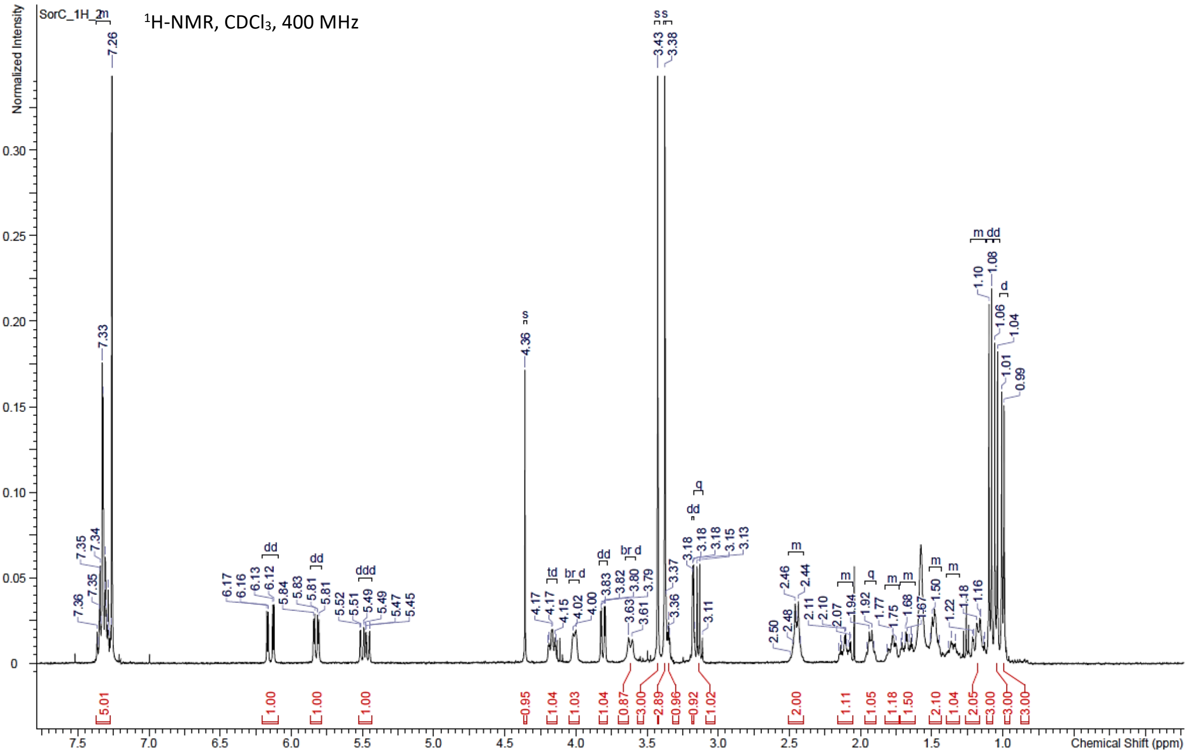

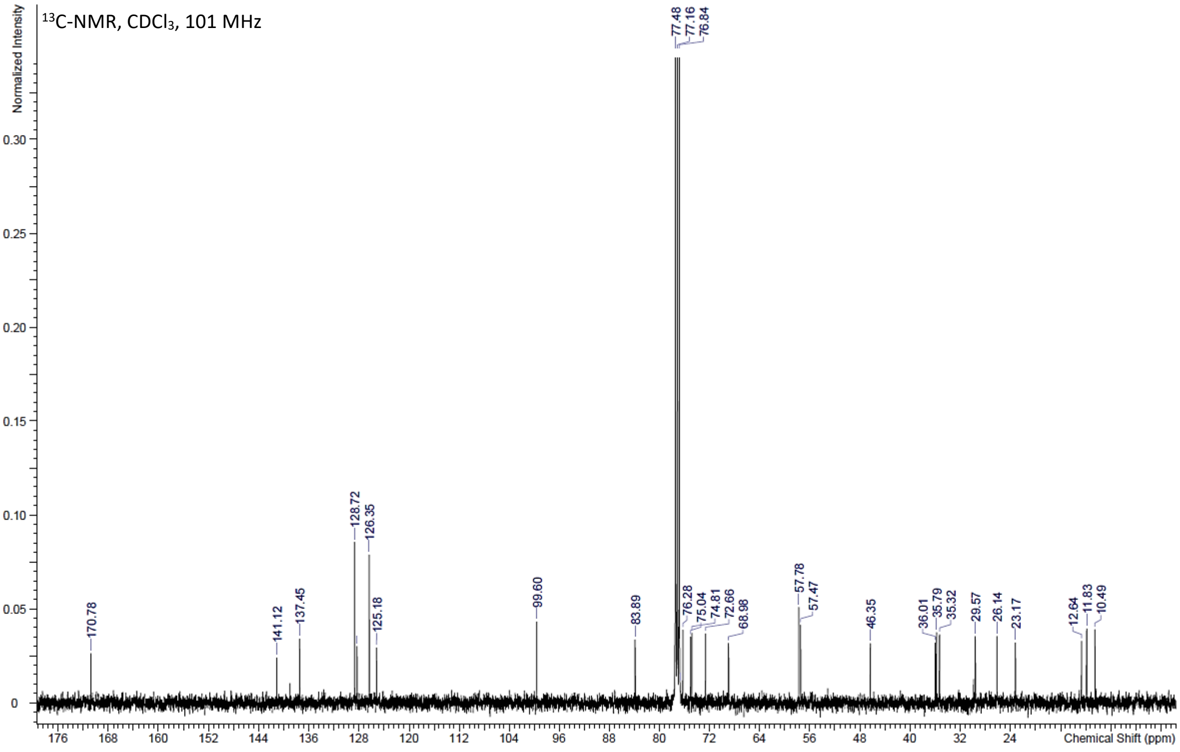

COSY, $CDCl_3$

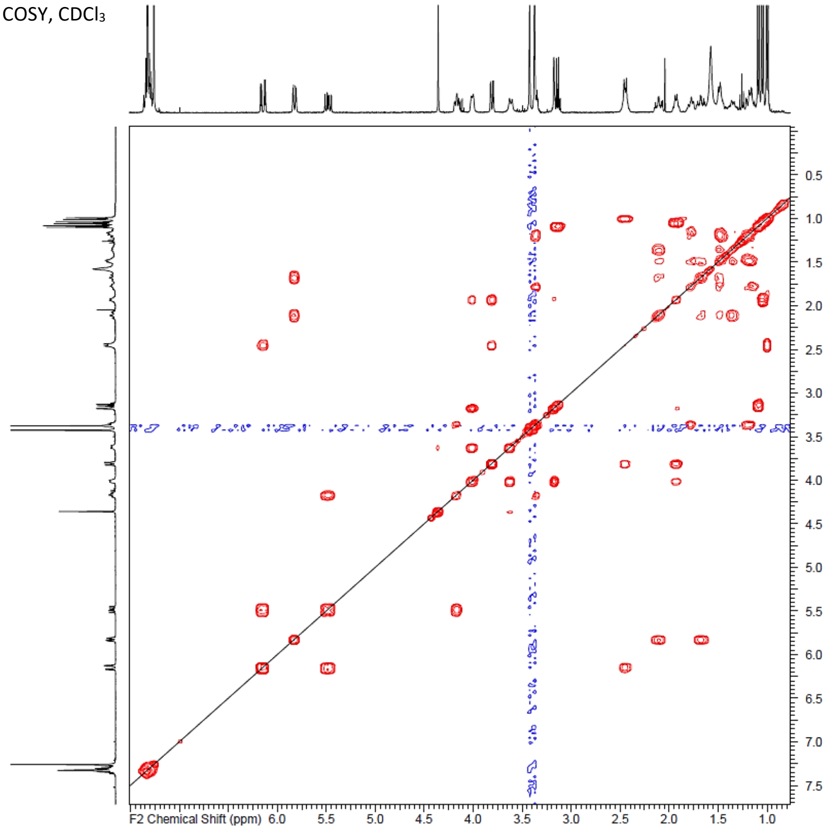

HSQC, $CDCl_3$

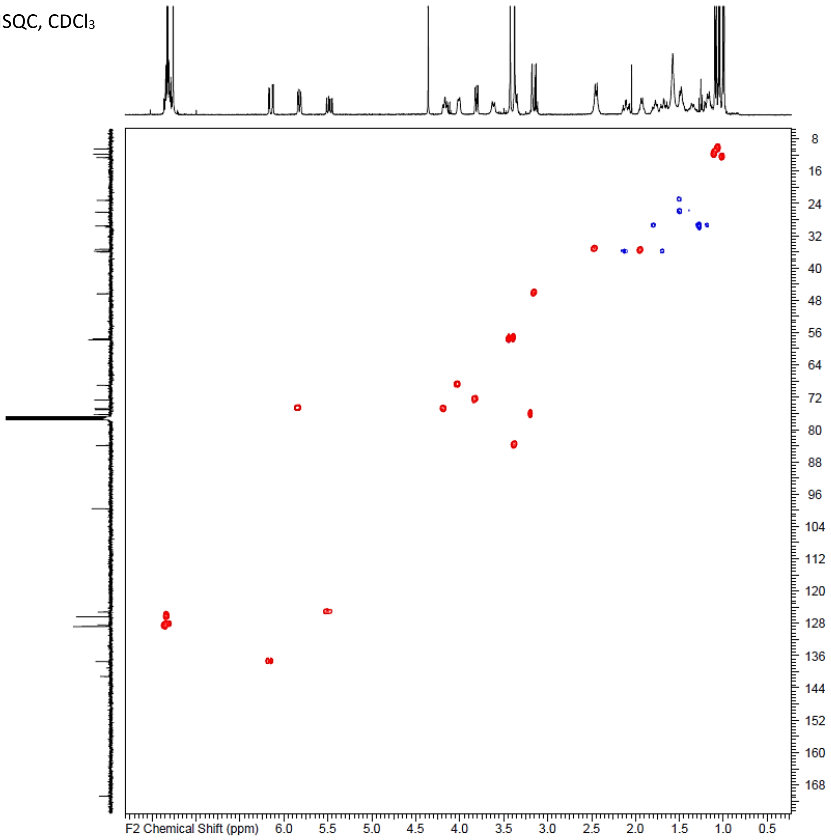

HMBC, $CDCl_3$

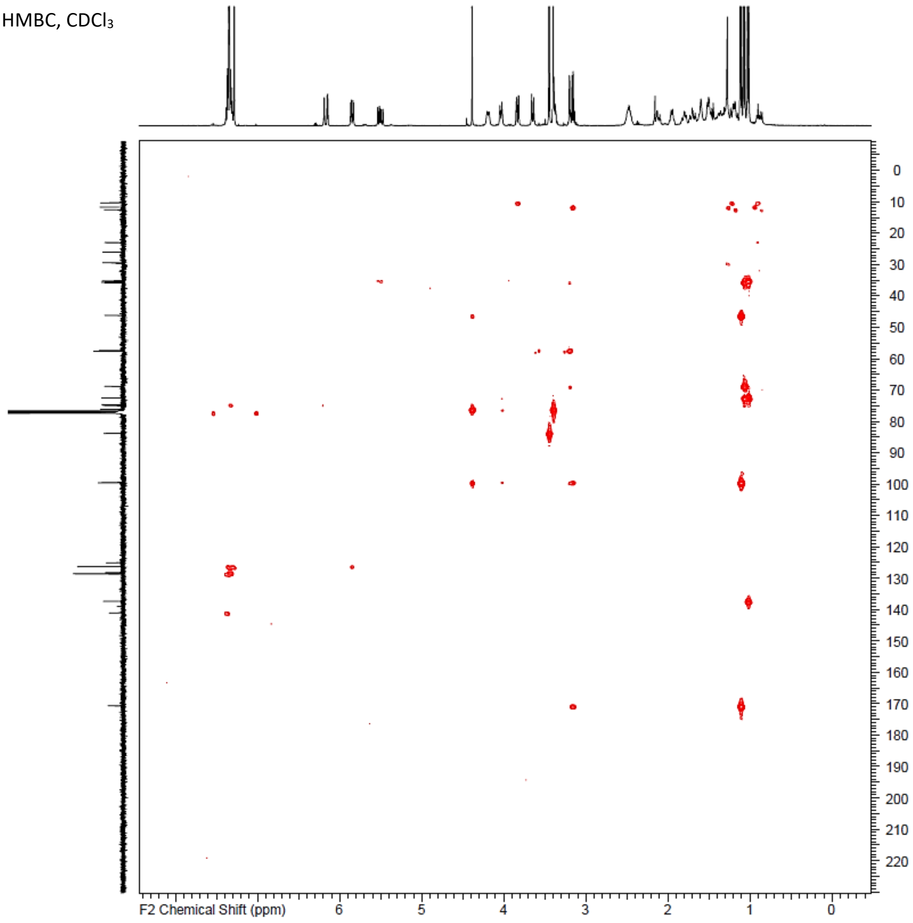

Supplementary Figure 24. NMR of compound *epi*-soraphen C, **S7**.

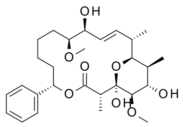

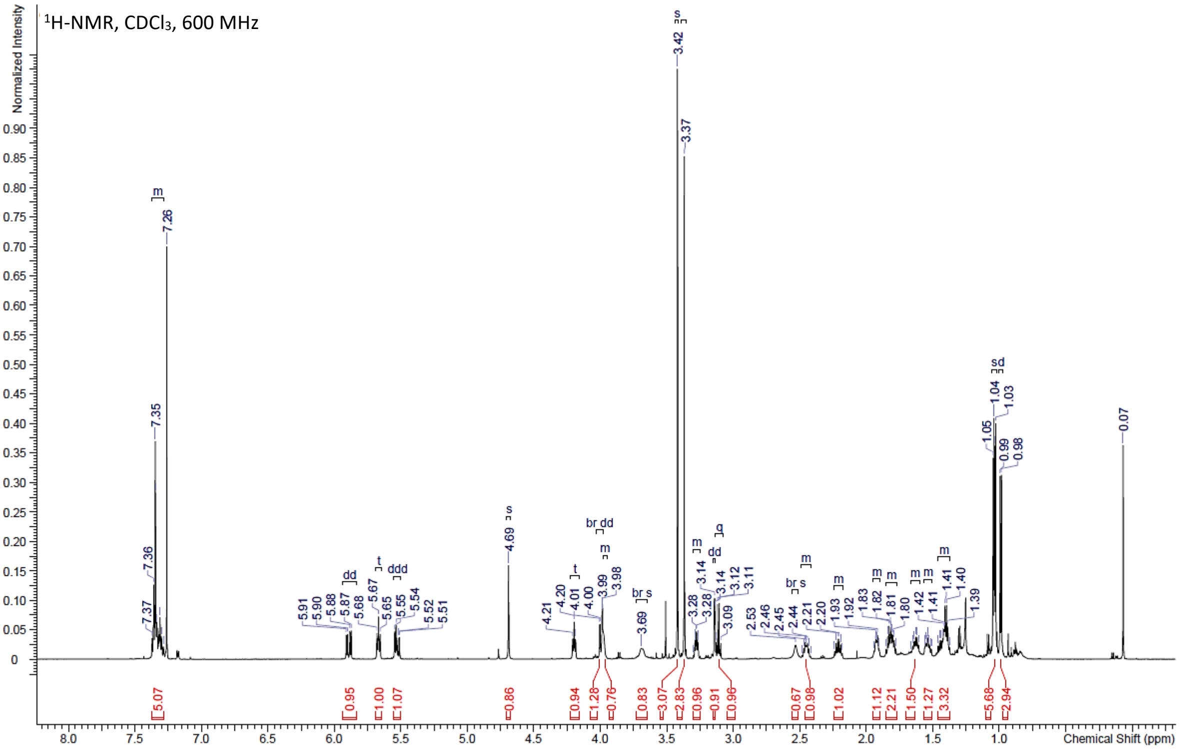

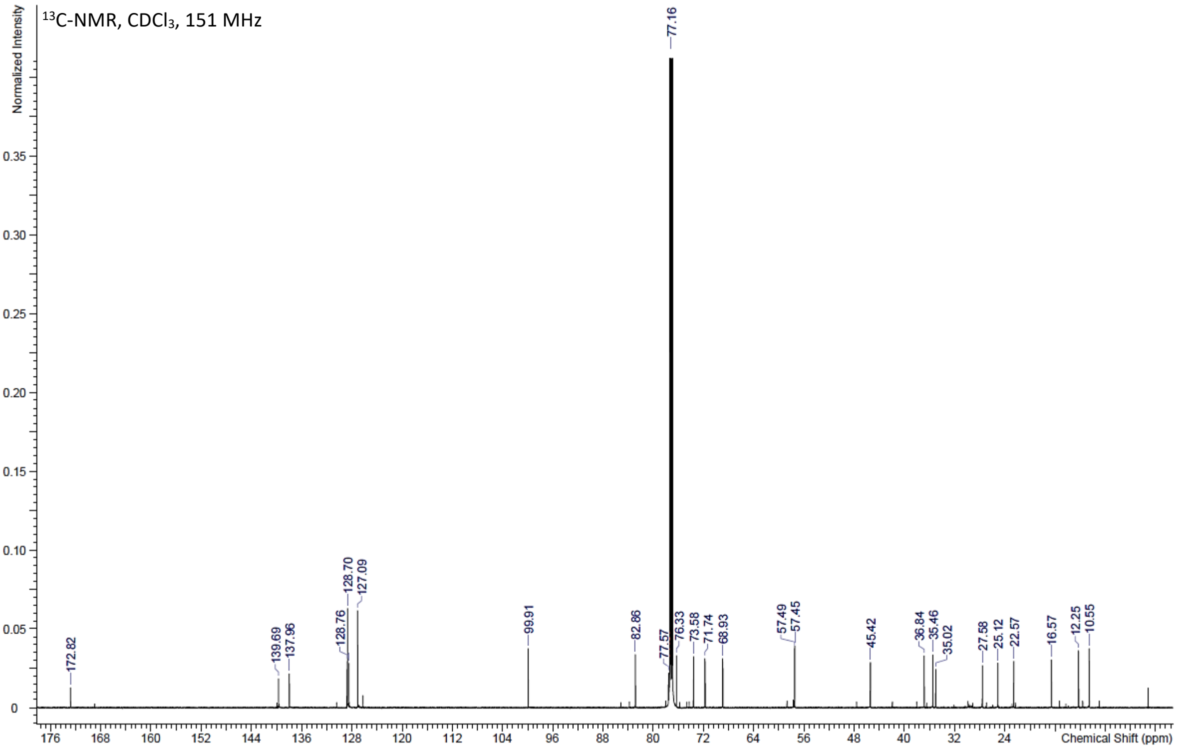

Supplementary Figure 25. NMR of compound **S6**.

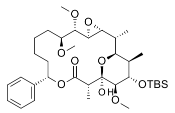

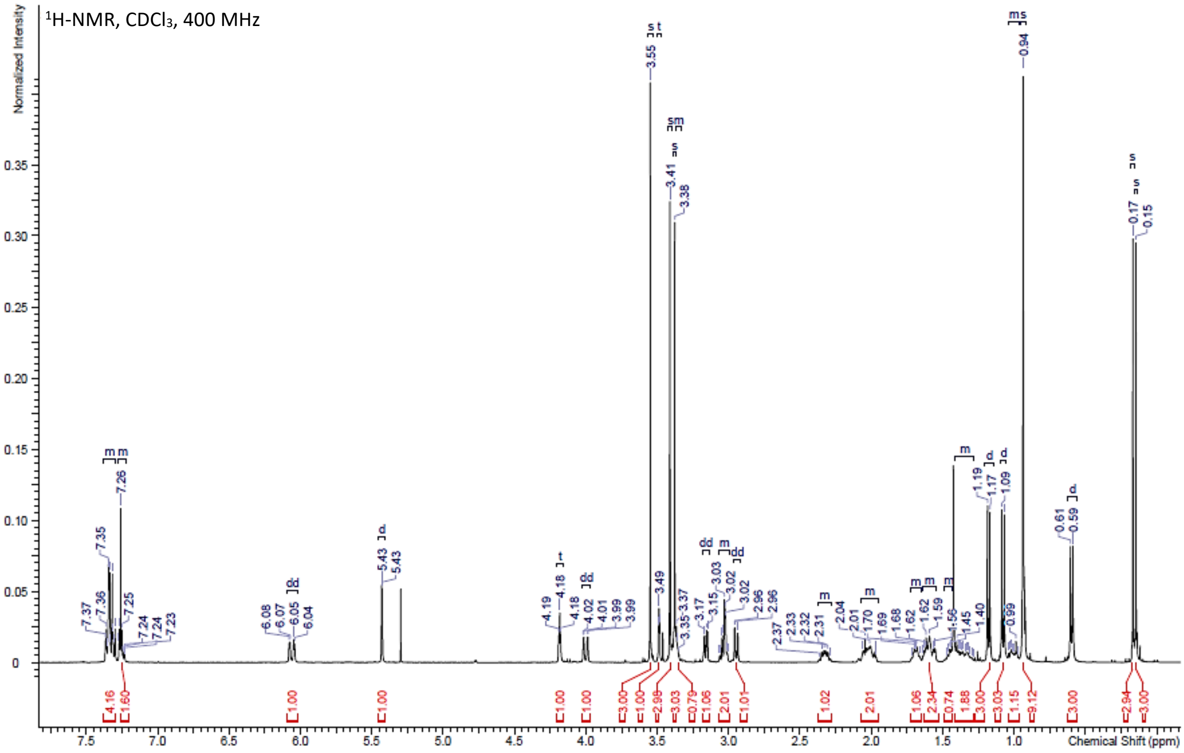

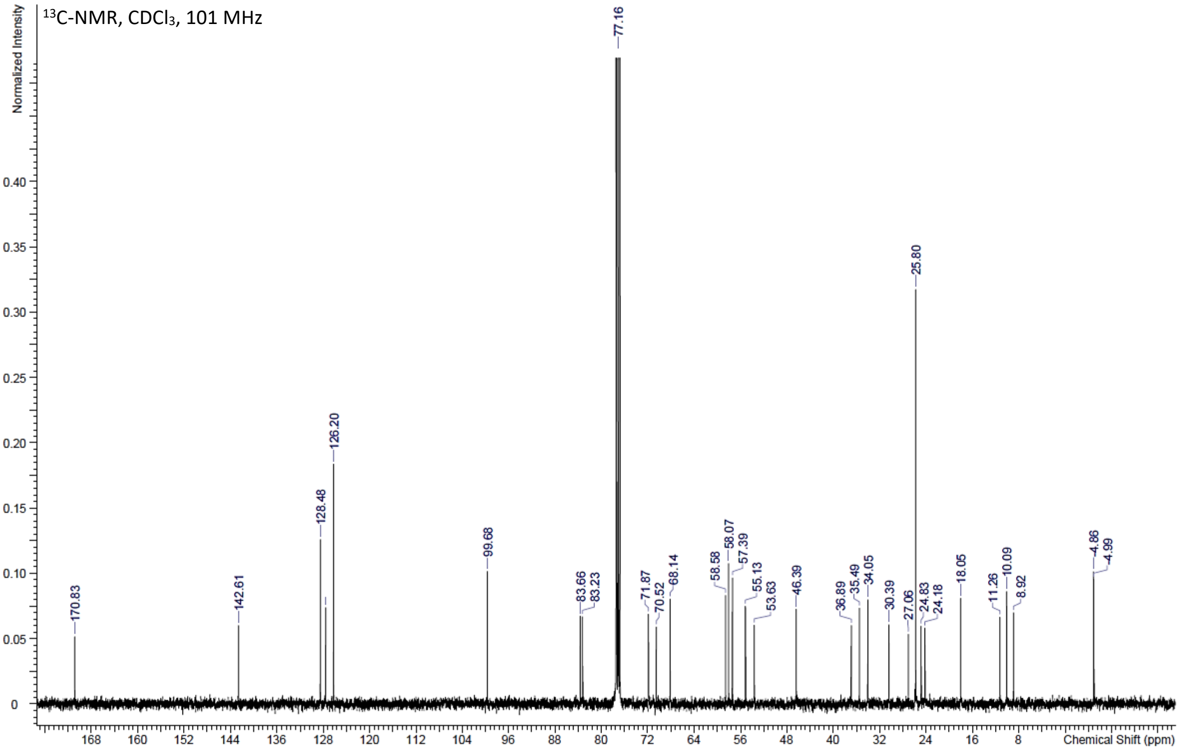

Supplementary Figure 26. NMR of compound **4**.

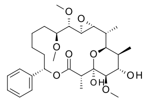

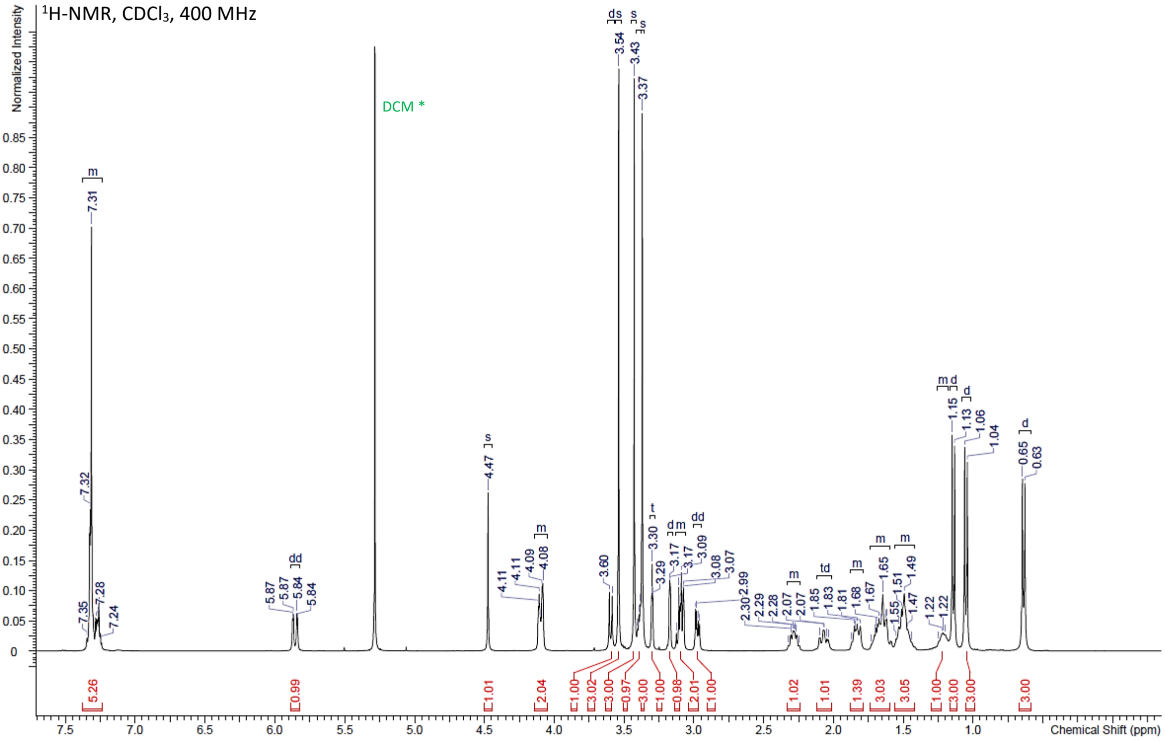

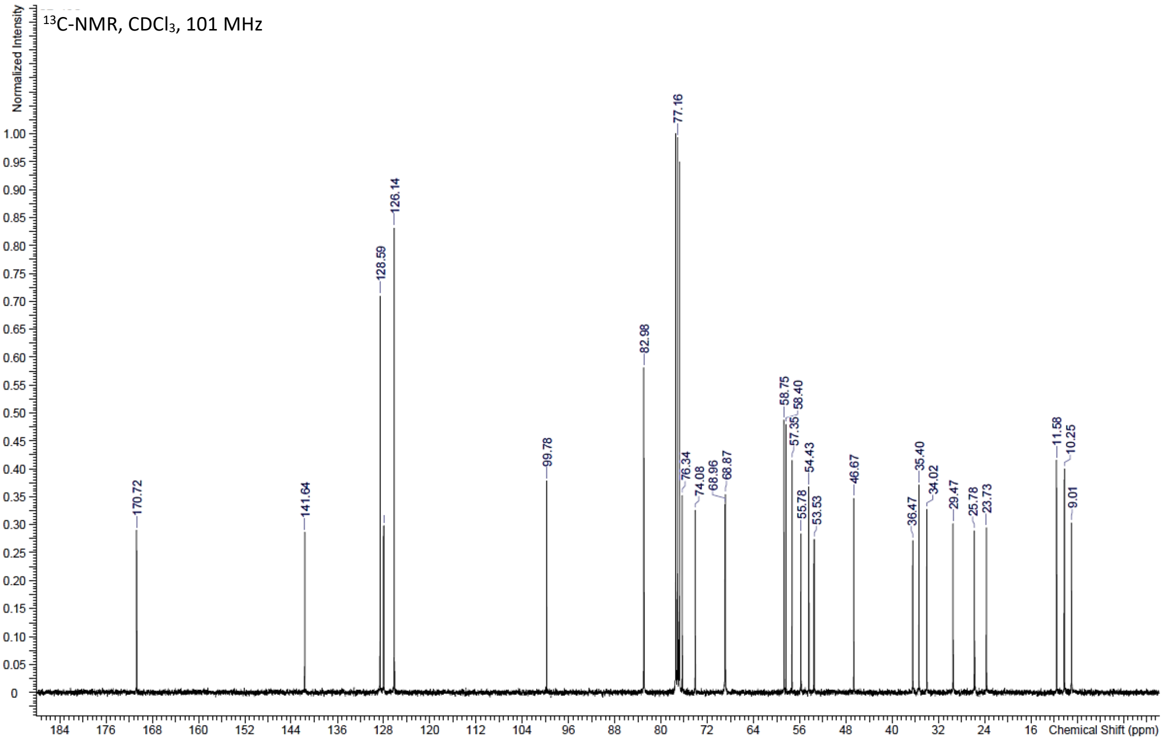

Supplementary Figure 27. NMR of compound **1a**.

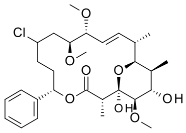

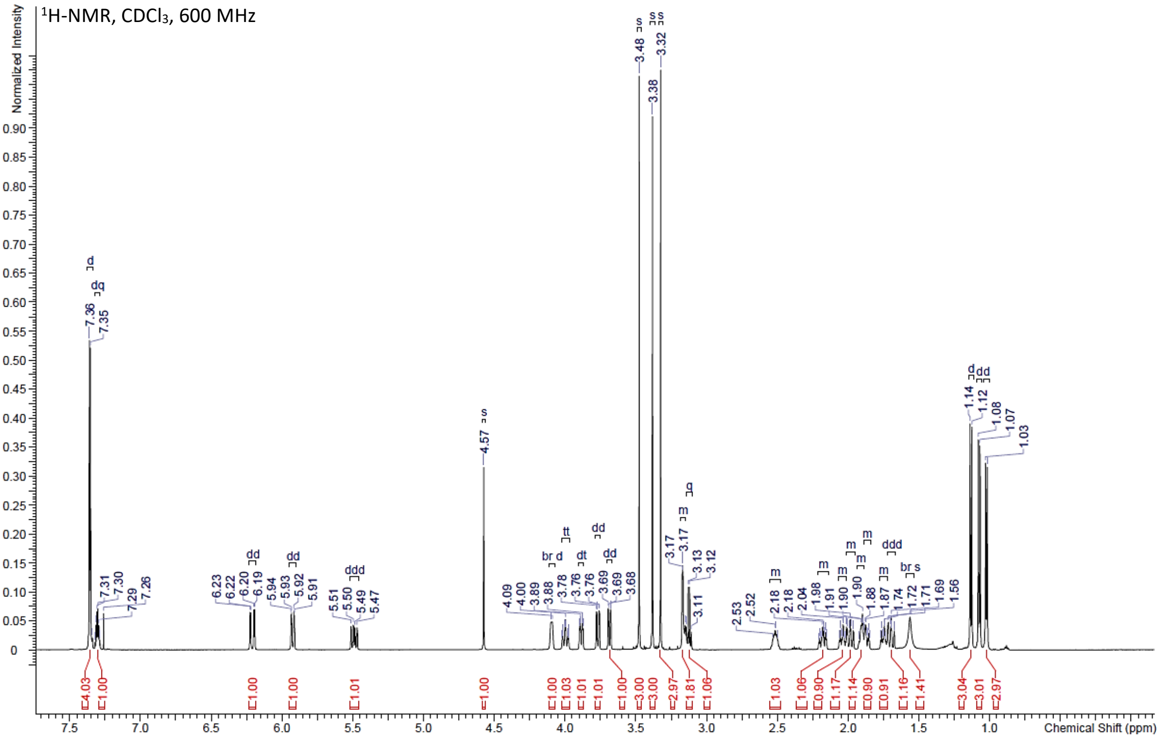

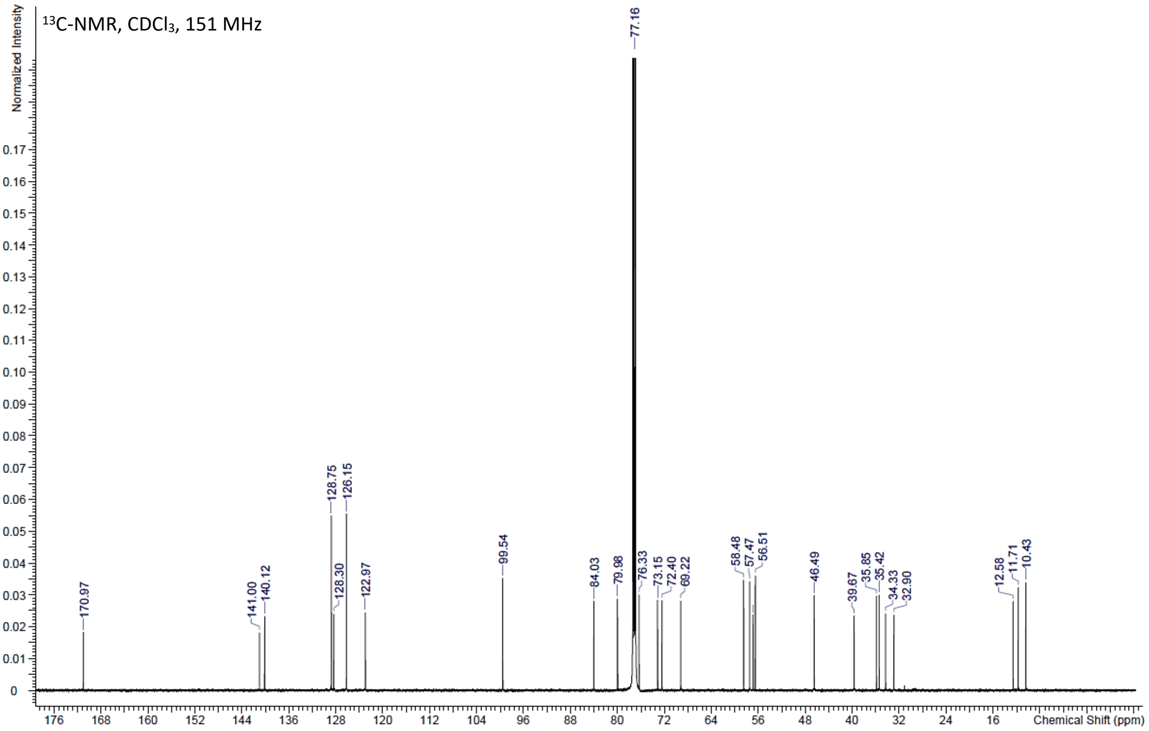

COSY, $CDCl_3$

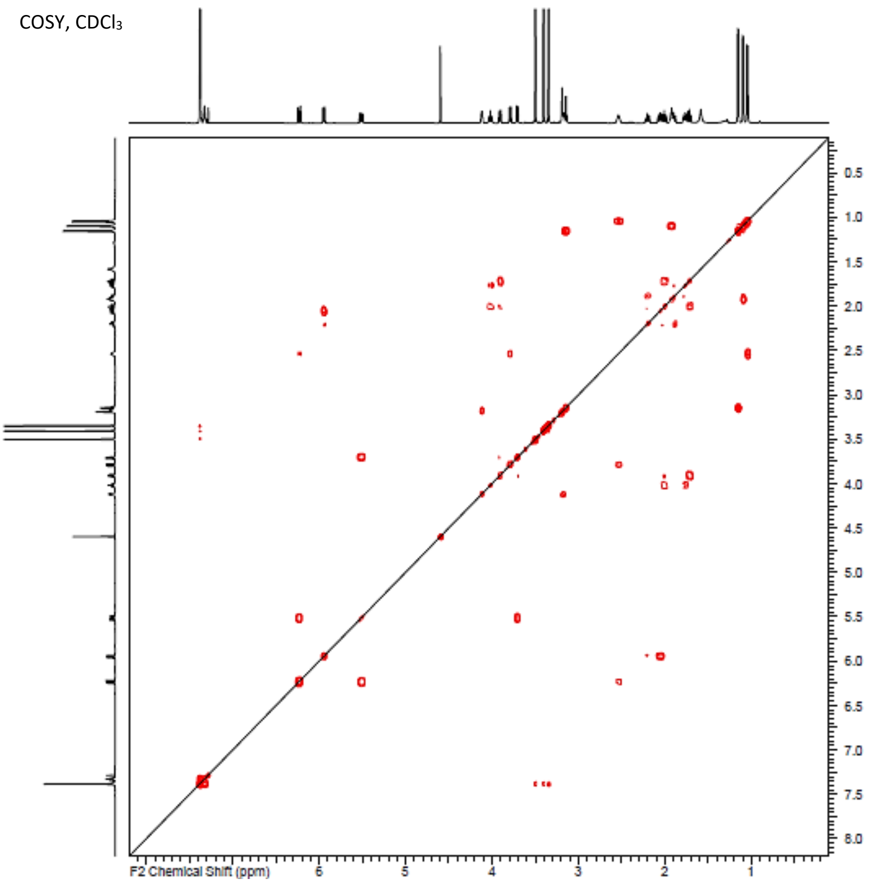

HSQC-DEPT, $CDCl_3$

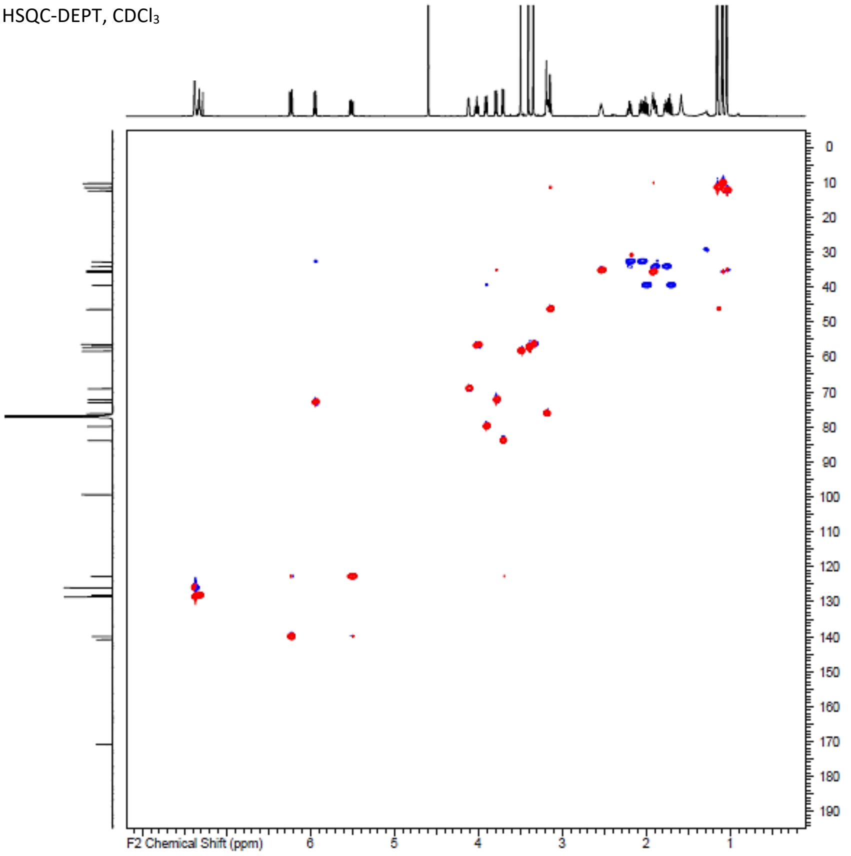

HMBC, CDCl₃

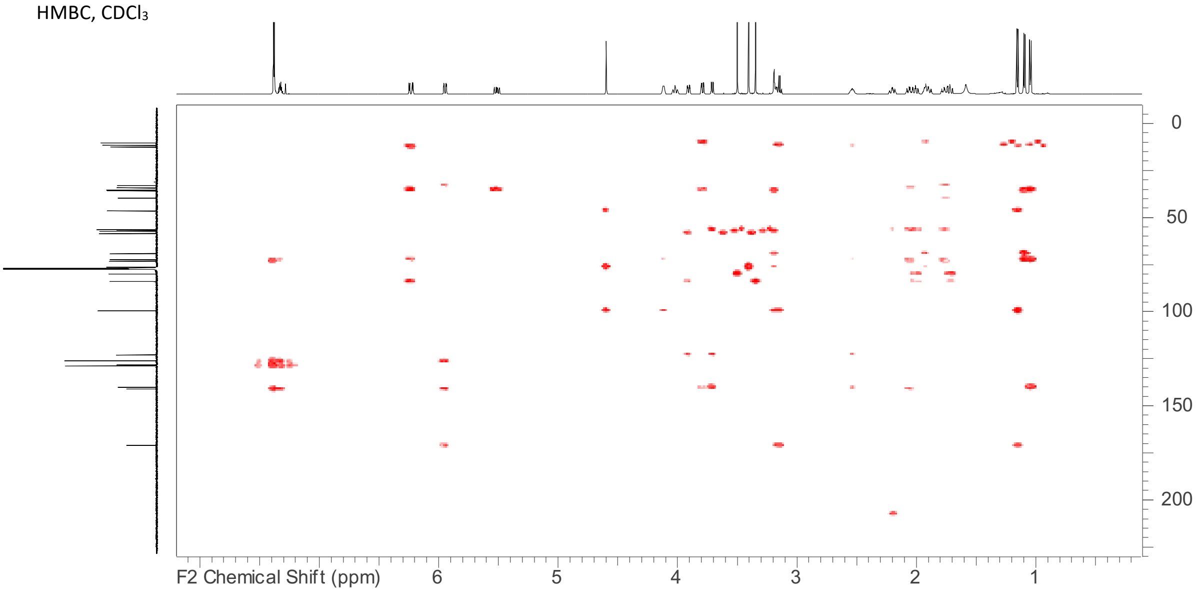

ROESY

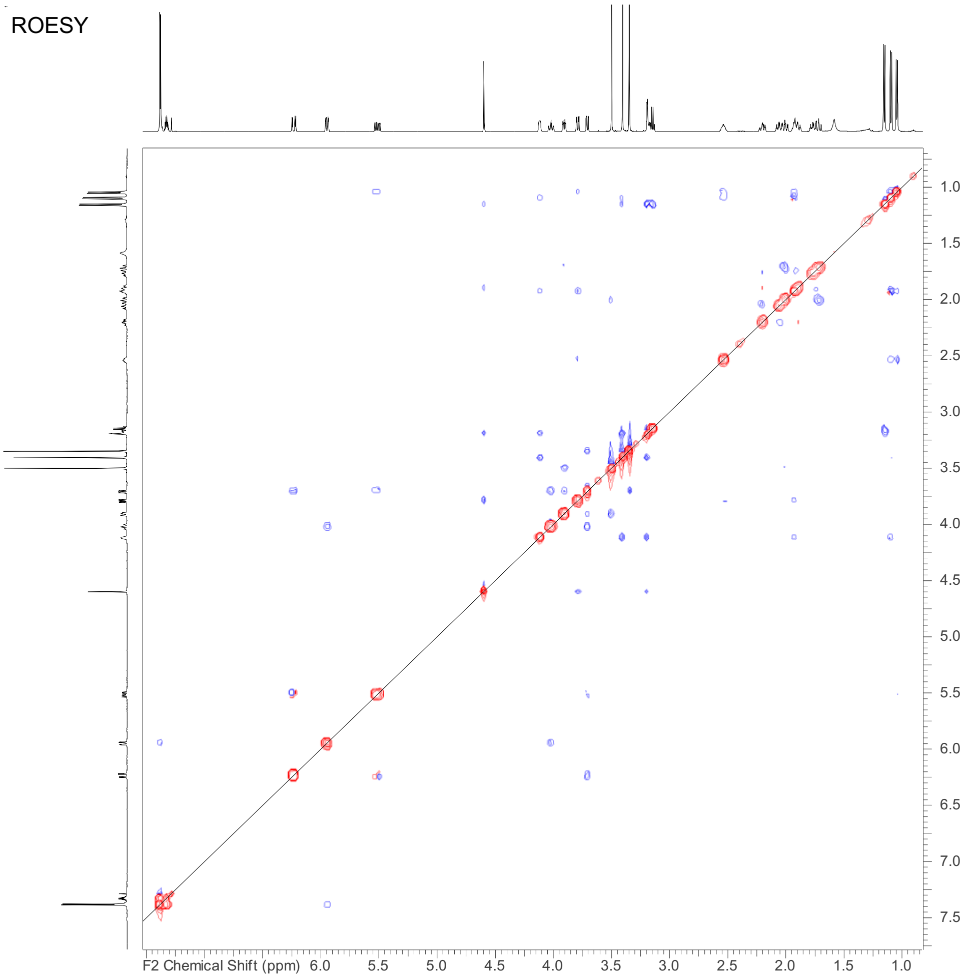

Supplementary Figure 28. NMR of compound **1c**.

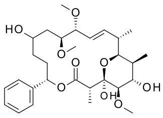

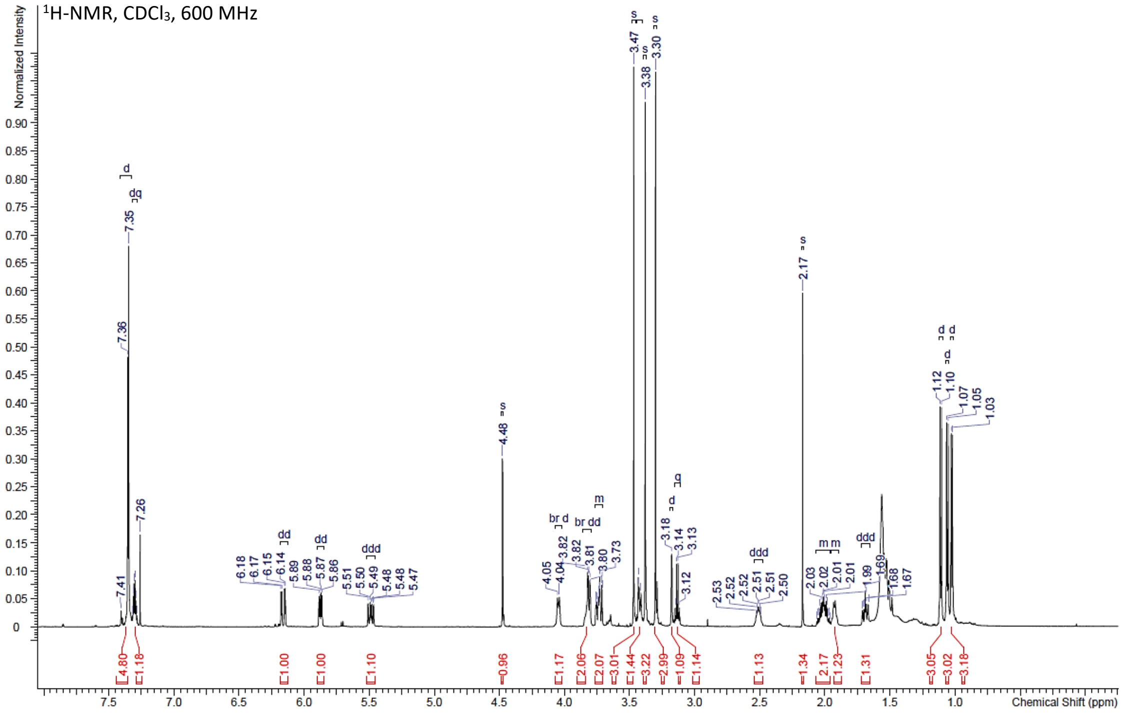

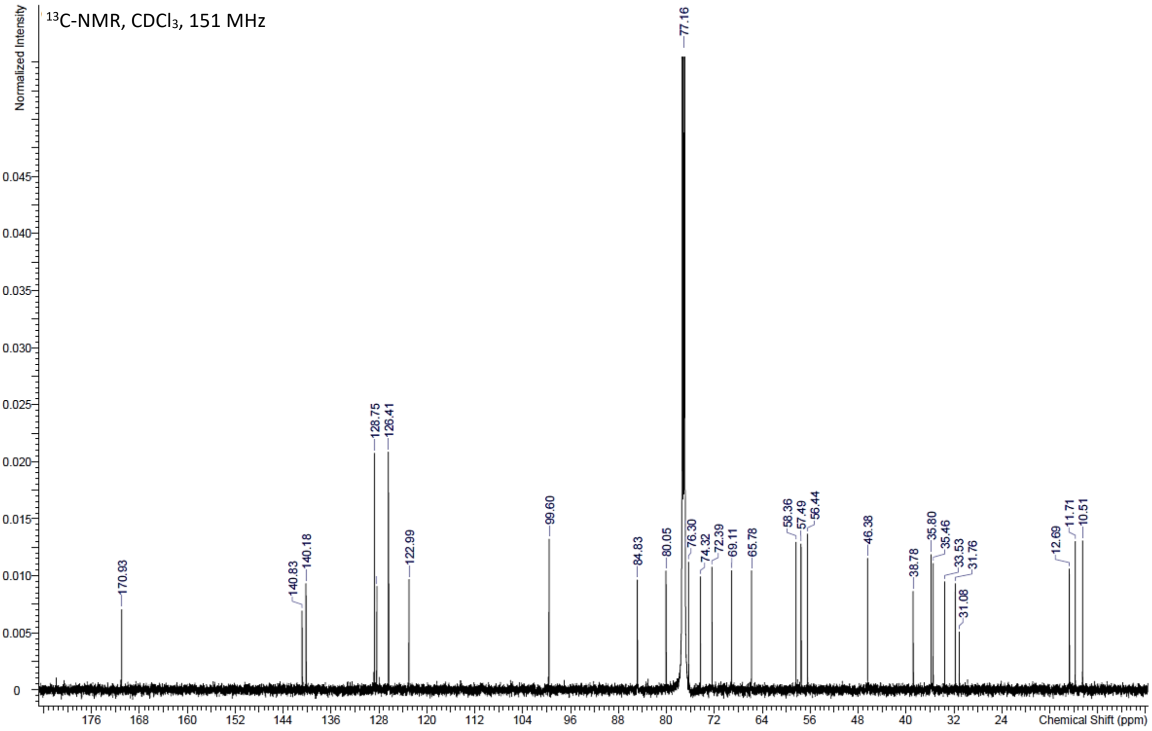

COSY, $CDCl_3$

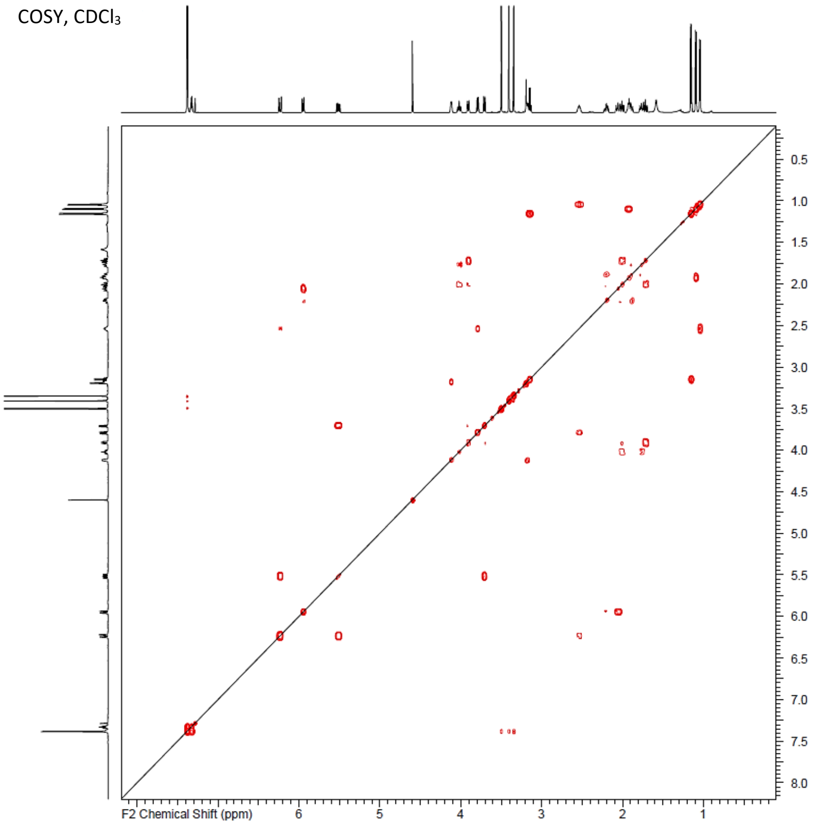

HSQC, $CDCl_3$

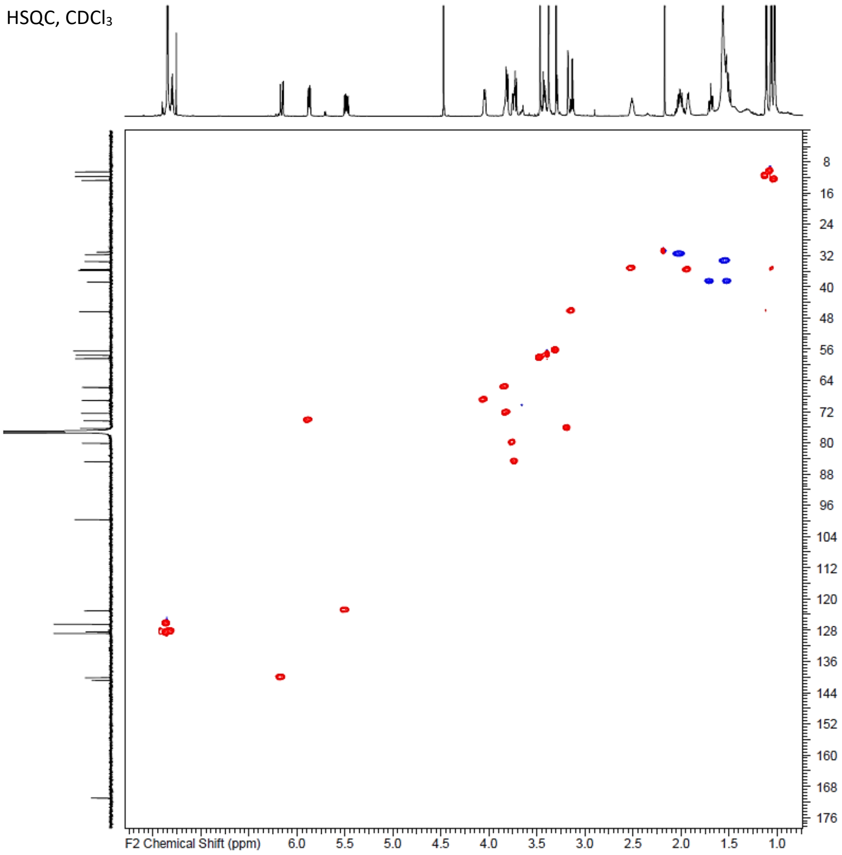

HMBC

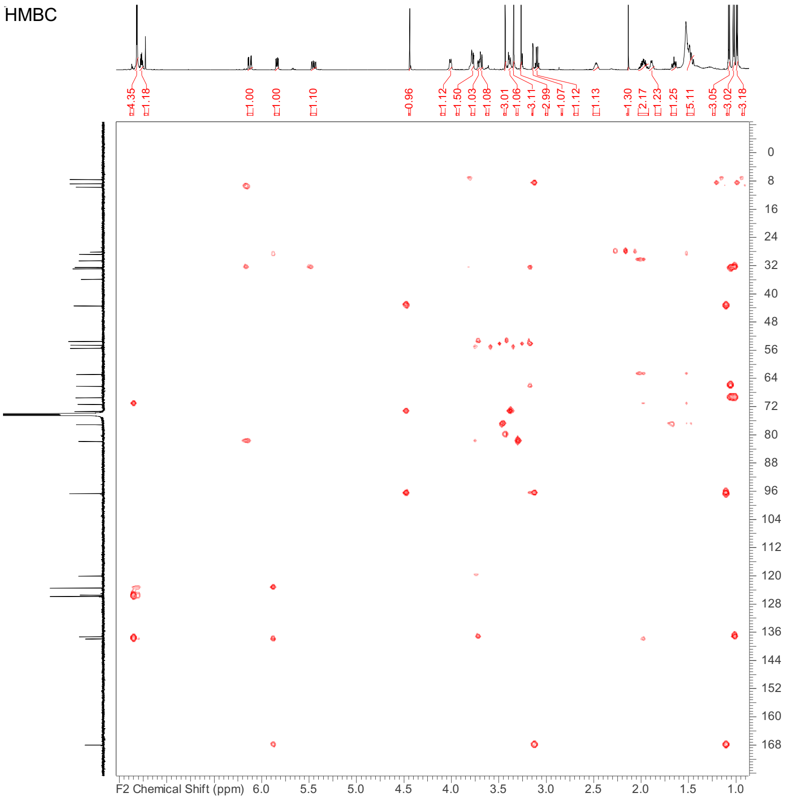

ROESY

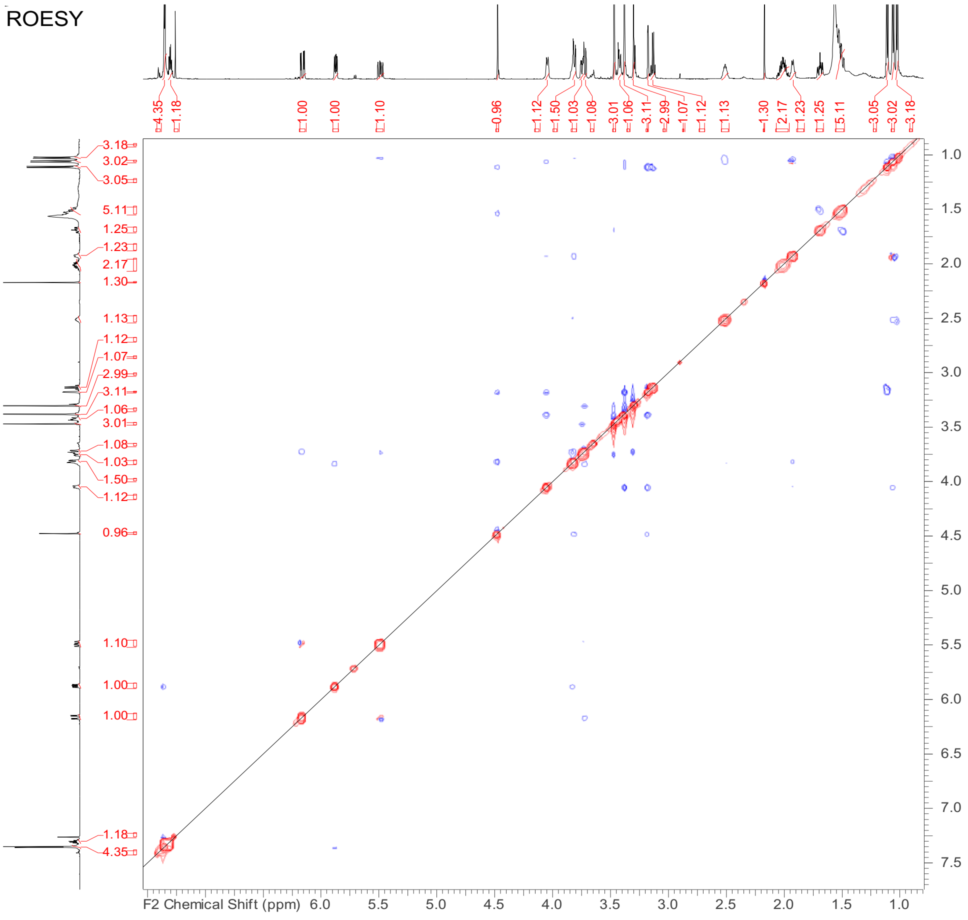

Supplementary Figure 29. NMR of compound **1b**.

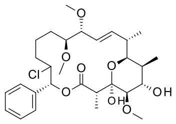

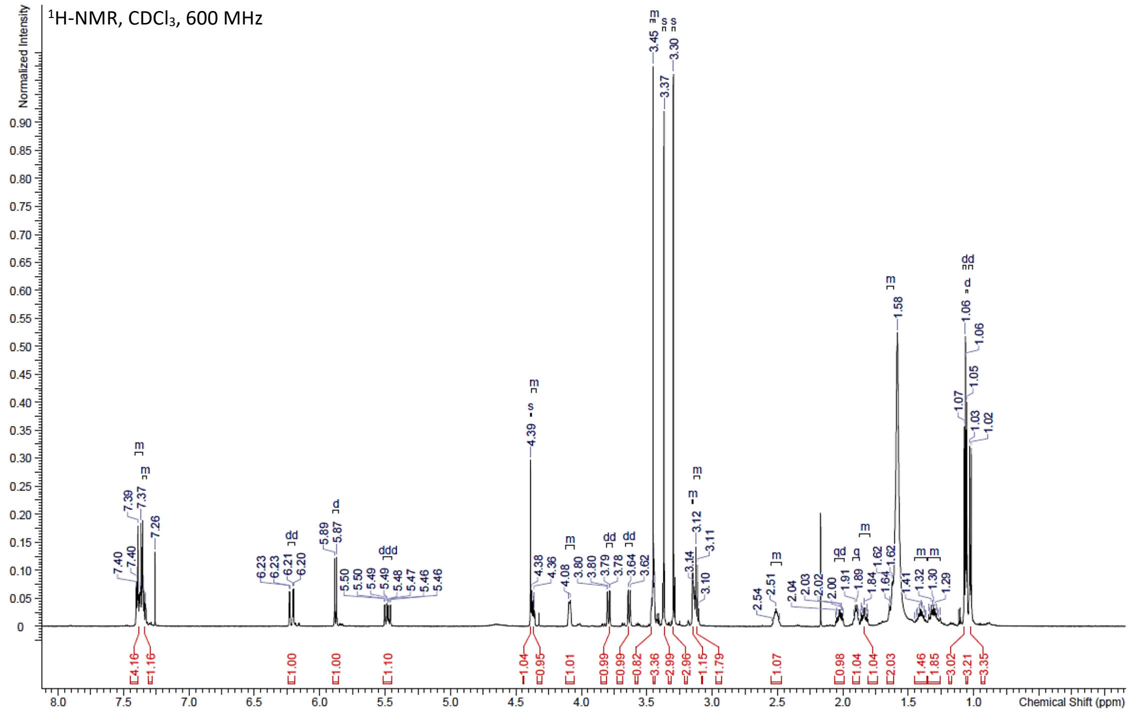

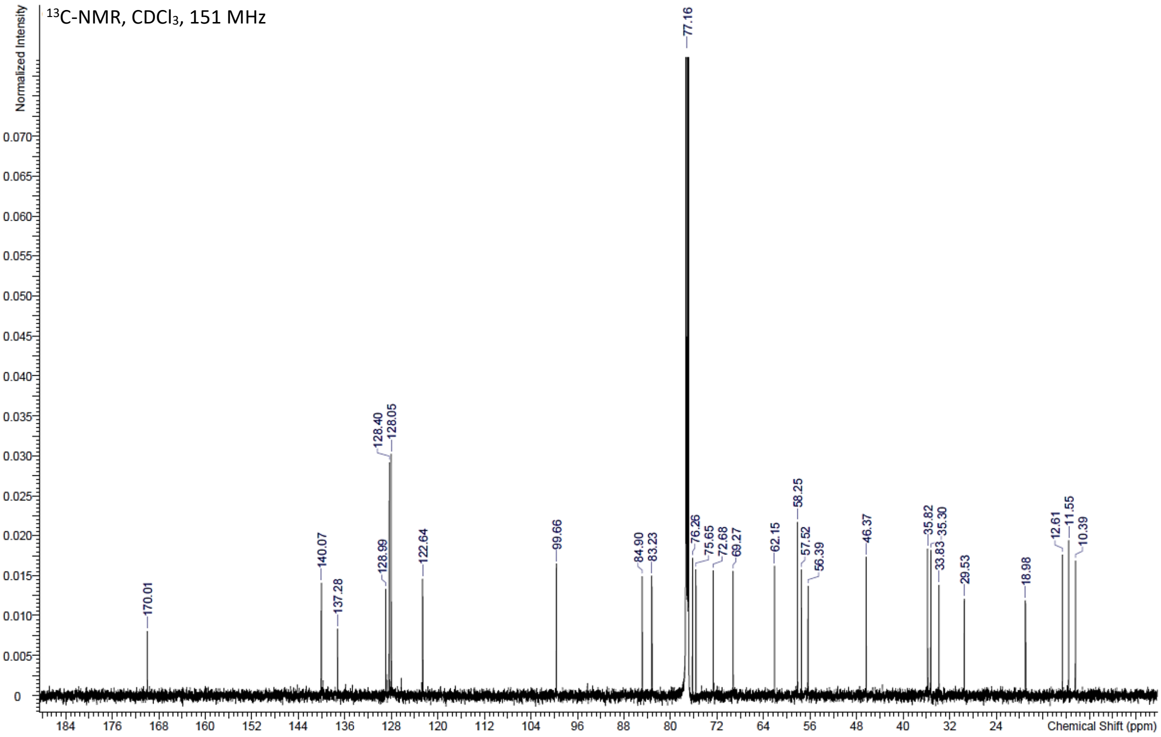

COSY, CDCl₃

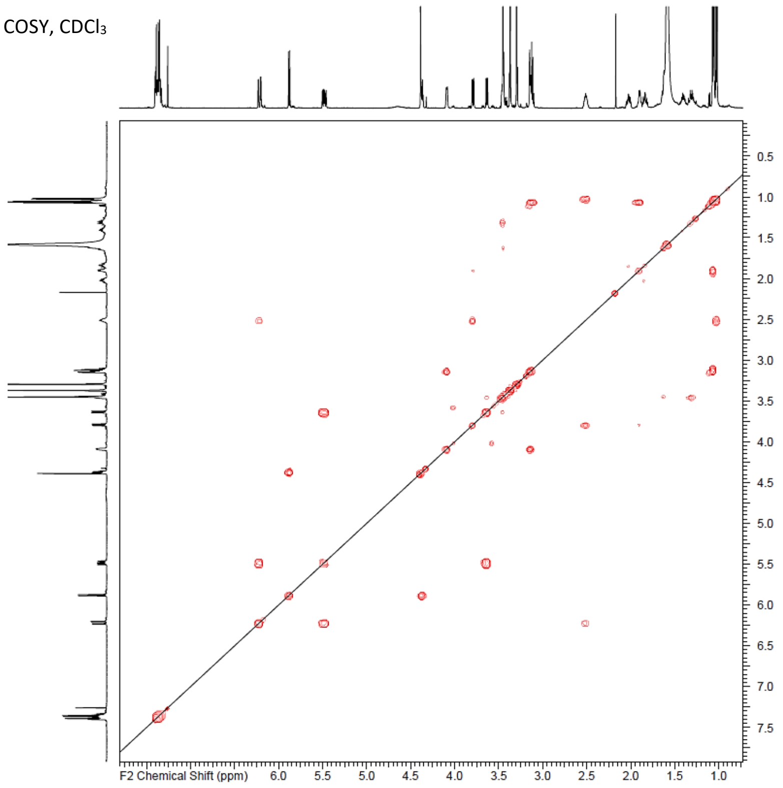

HSQC, CDCl₃

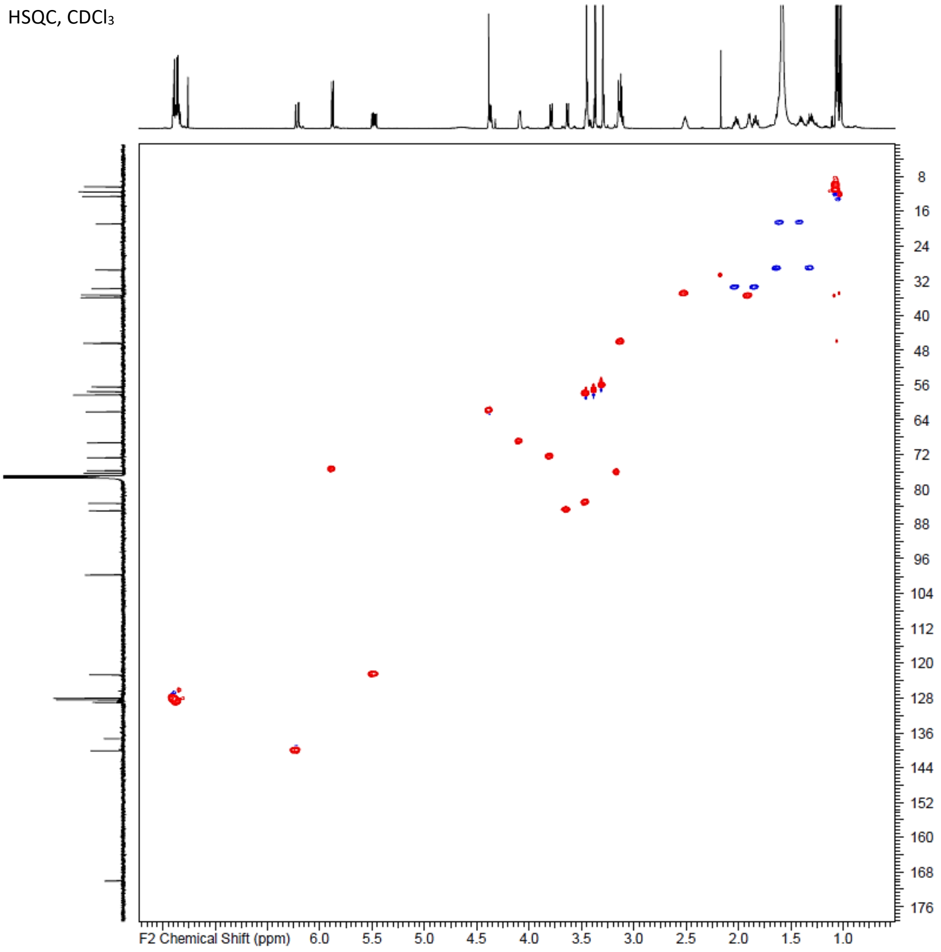

HMBC

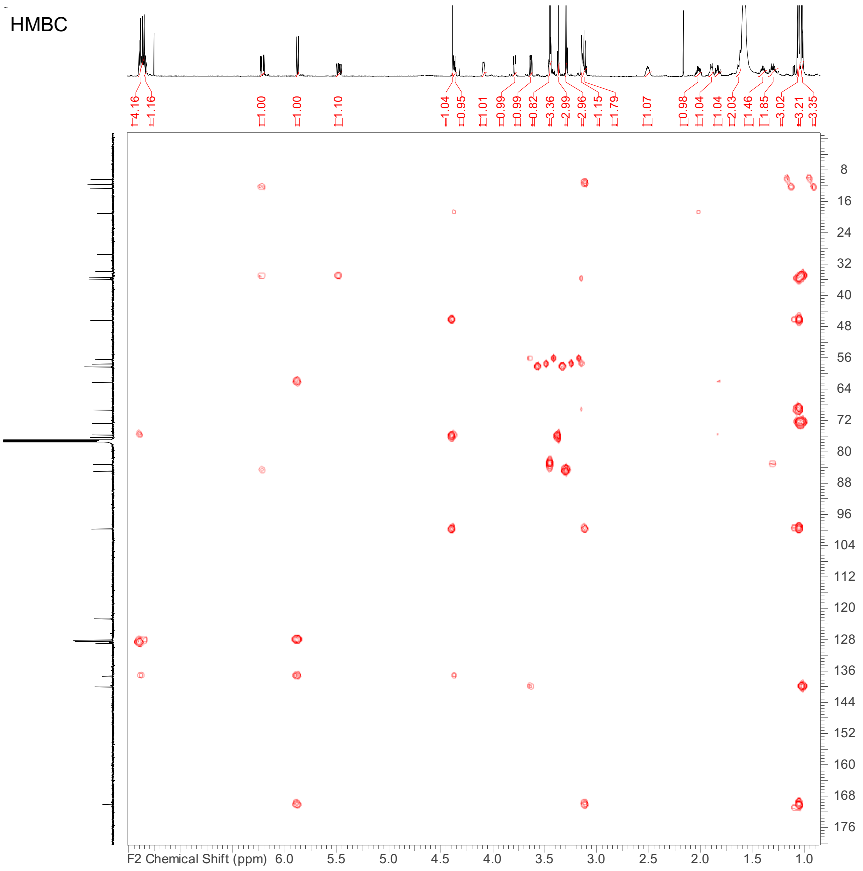

ROESY

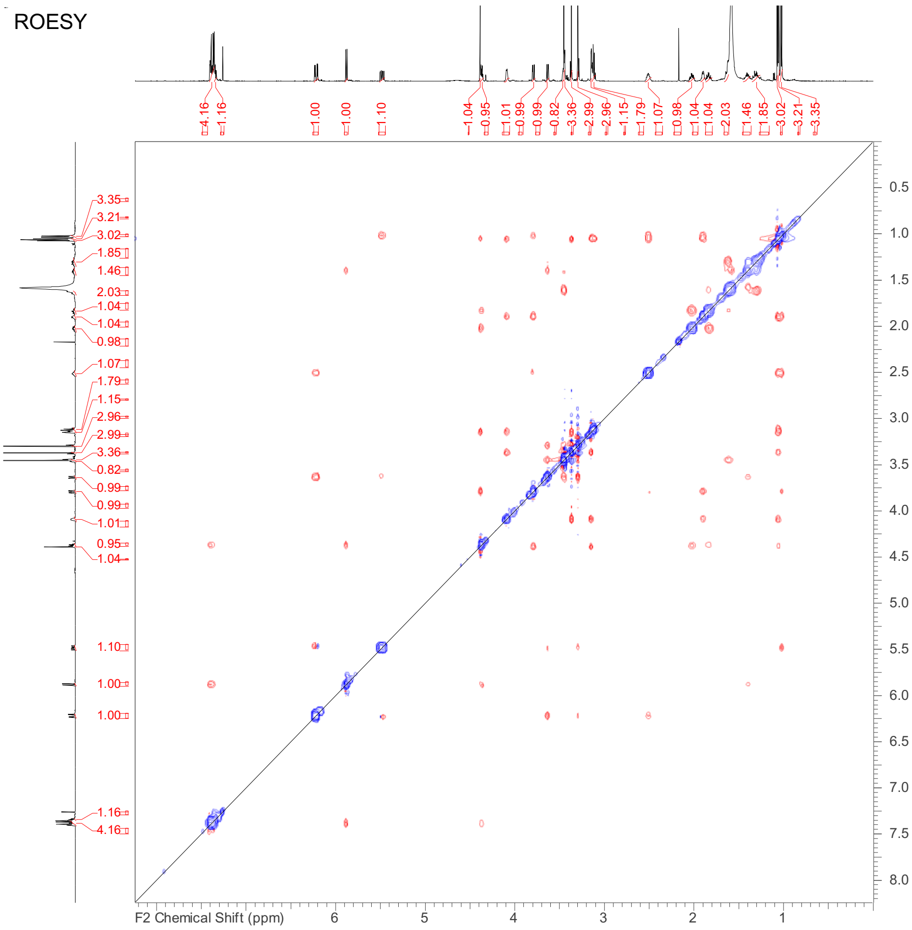

Supplementary Figure 30. NMR of compound **2a**.

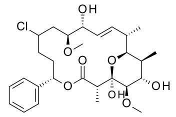

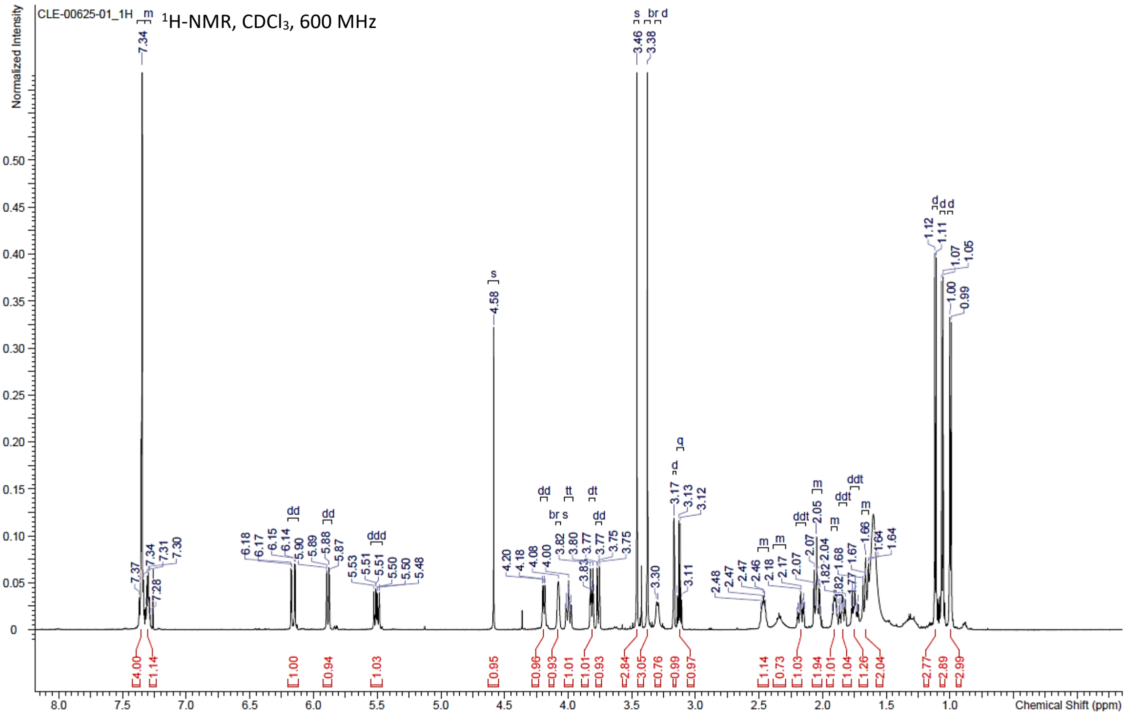

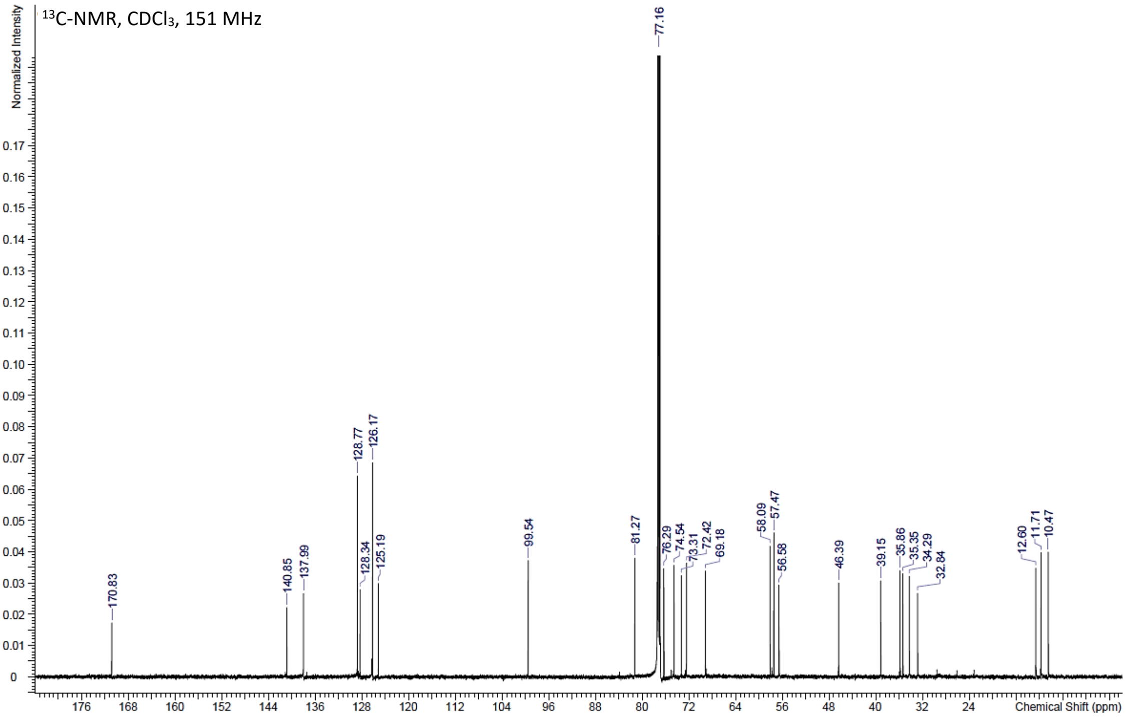

COSY, $CDCl_3$

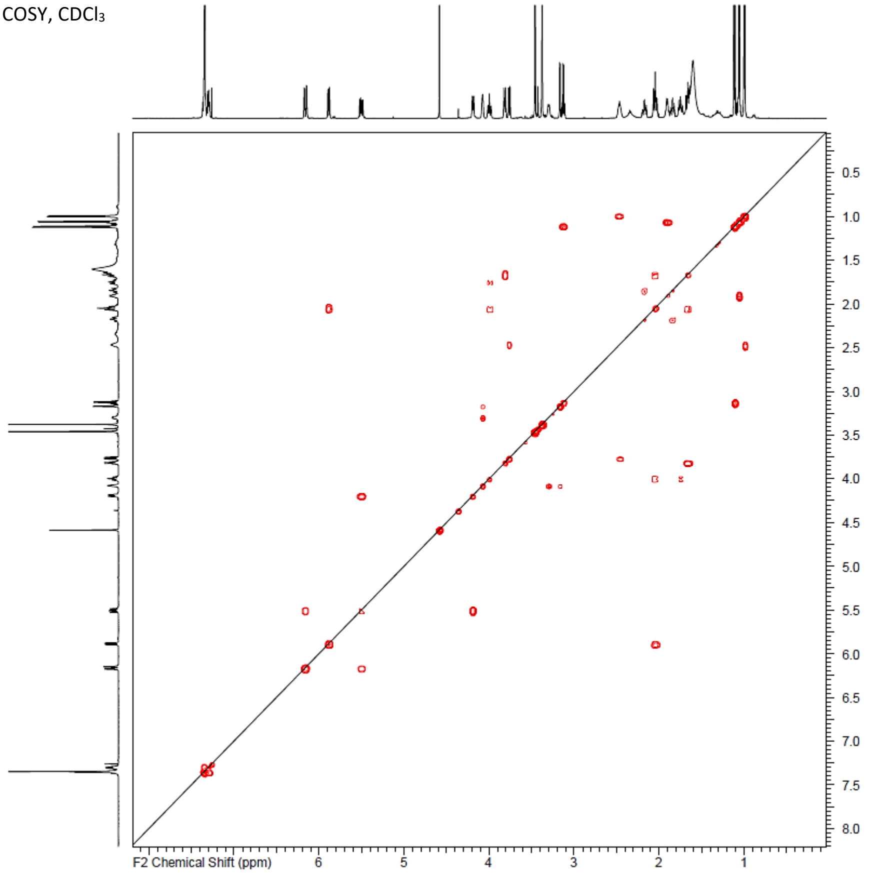

HSQC, $CDCl_3$

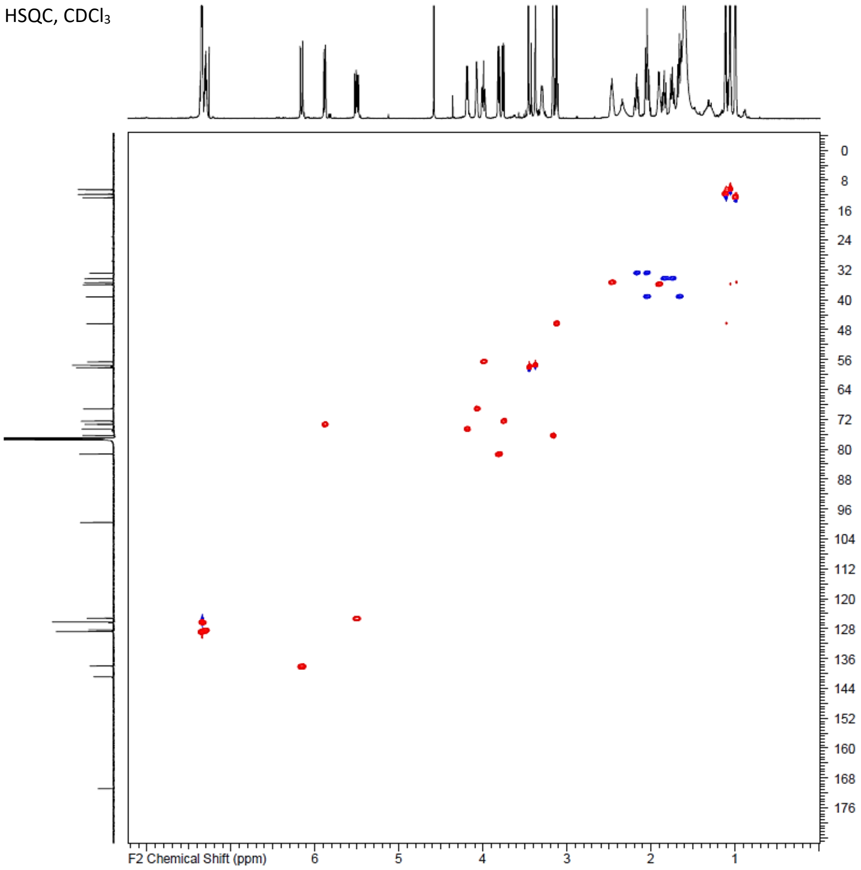

HMBC

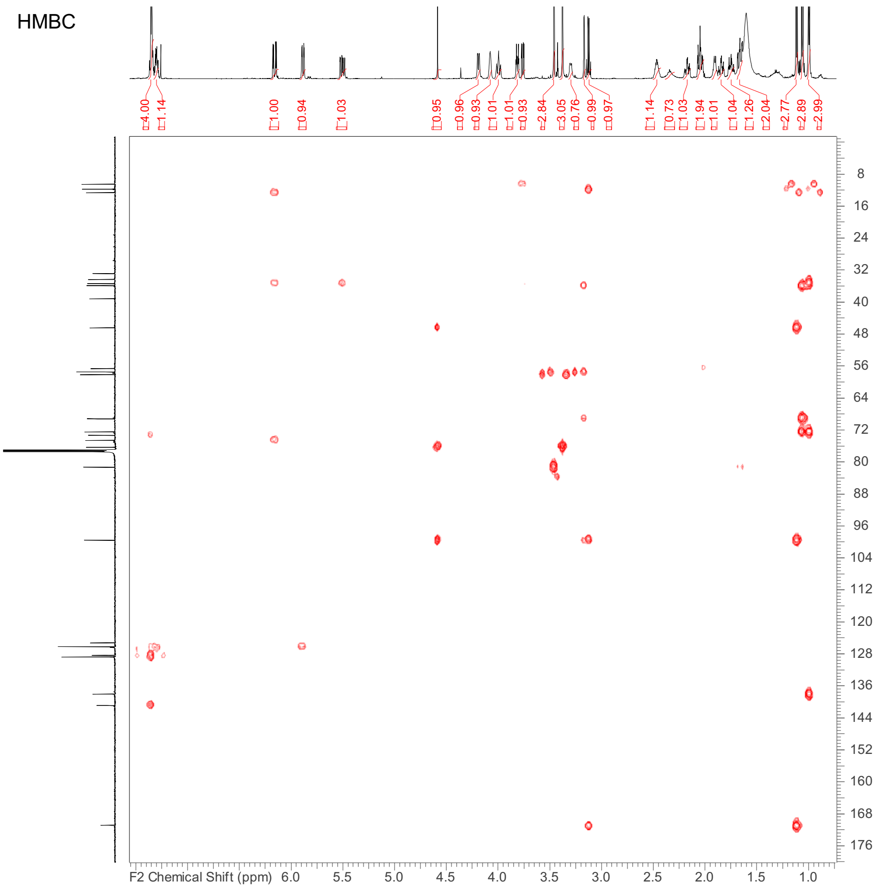

ROESY

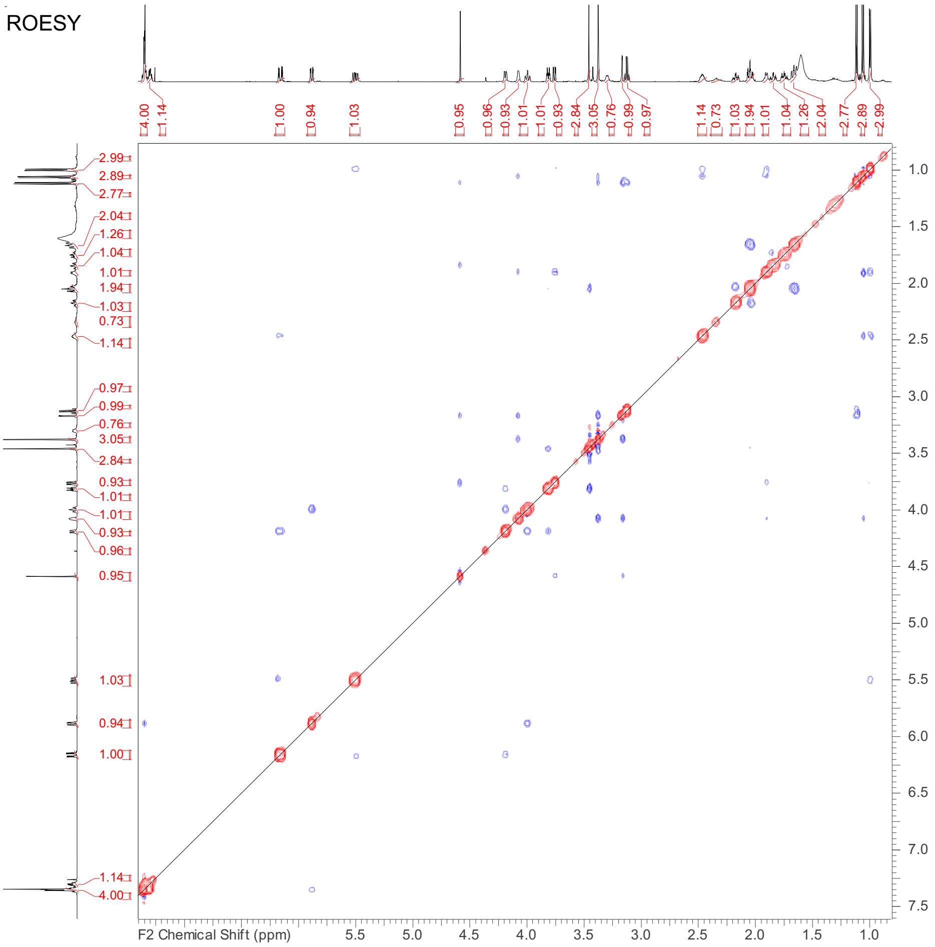

Methods for biological testing and BP80 determination

The assays were performed at Syngenta's high-throughput screening facilities, using standardized assays and the necessary standards.

Leaf disk assays:

Erysiphe graminis f.sp. tritici (Wheat powdery mildew): preventive application
Barley leaf segments were placed on agar in multiwell plates (24-well format) and sprayed with test solutions. After drying, the leaf disks were inoculated with spores of the fungus. After appropriate incubation the activity of a compound was assessed 7 dpi (days post inoculation) as preventive fungicidal activity.

Puccinia recondita (Brown rust): curative application
Wheat leaf segments are placed on agar in multiwell plates (24-well format). The leaf disks are then inoculated with a spore suspension of the fungus. One day after inoculation the test solution is applied. After appropriate incubation the activity of a compound is assessed 8 dpi (days post inoculation) as curative fungicidal activity.

Liquid culture assays:

Botrytis cinerea (Gray mould):
Conidia of the fungus from cryogenic storage were directly mixed into nutrient broth (Vogel's minimal media). A DMSO solution of the test compounds was placed into a microtiter plate (96-well format) and the nutrient broth containing the fungal spores was added to it. The test plates were incubated at 24 °C and the inhibition of growth was determined photometrically after 72 hours at 620 nm.

Mycosphaerella arachidis (Brown leaf spot of peanut):
Conidia of the fungus from cryogenic storage were directly mixed into nutrient broth (PDB potato dextrose broth). A DMSO solution of the test compounds was placed into a microtiter plate (96-well format) and the nutrient broth containing the fungal spores was added to it. The test plates were incubated at 24 °C and the inhibition of growth was determined photometrically after approximately 5-6 days at 620 nm.

Septoria tritici (leaf blotch):
Conidia of the fungus from cryogenic storage were directly mixed into nutrient broth (PDB potato dextrose broth). A DMSO solution of the test compounds was placed into a microtiter plate (96-well format) and the nutrient broth containing the fungal spores was added to it. The test plates were incubated at 24 °C and the inhibition of growth was determined photometrically after 72 hours at 620 nm.

Monographella nivalis (snow mould, foot rot of cereals):
Conidia of the fungus from cryogenic storage were directly mixed into nutrient broth (PDB potato dextrose broth). A DMSO solution of the test compounds was placed into a microtiter plate (96-well format) and the nutrient broth containing the fungal spores was added to it. The test plates were incubated at 24 °C and the inhibition of growth was determined photometrically after 72 hours at 620 nm.

BP80 determination:
A dilution series was performed for each fungus/compound combination and the efficiency of the compounds evaluated. BP80 represents the breakpoint below which less than 80% efficiency is observed.

The experiments on living organisms were performed as single or triplicate experiments, against positive and negative standards (both commercial and company internal). The triplicate experiments were technical triplicates, with three samples tested in parallel during the same test session. The pest control percentage is assessed visually by a trained personal and is assigned to be 100%, 90%, 70%, 50%, 20% or 0%. The BP80 was determined as an average over all the results.

Supplementary Table 13. BP80 determination for compound **1**.

Compound 1								
**Pathogen tested**	**Rate of application in ppm**	**Rate of application in uM**	**% Pest control**				**pBP80**	**BP80 (uM)**
			**Test 1**	**Test 2**				
				replicate 1	replicate 2	replicate 3		
Erysiphe graminis	22.2	42.6	100	100	100	100	5.8	0.16
	7.39	14.2	100	100	100	100		
	2.46	4.7	100	100	100	100		
	0.819	1.6	100	100	100	100		
Puccinia recondita	22.2	42.6	100	100	100	100	5.8	0.16
	7.39	14.2	100	100	100	100		
	2.46	4.7	100	100	100	100		
	0.819	1.6	100	100	100	100		
Botrytis cinerea	6.67	12.8	100	100	100	100	6.8	0.016
	2.22	4.3	100	100	100	100		
	0.739	1.4	100	100	100	100		
	0.246	0.5	100	100	100	100		
Mycosphaerella arachidis	6.67	12.8	100	100	100	100	6.3	0.050
	2.22	4.3	100	100	100	100		
	0.739	1.4	100	100	100	100		
Septoria tritici	6.67	12.8	100	100	100	100	6.3	0.050
	2.22	4.3	100	100	100	100		
	0.739	1.4	100	100	100	100		
	0.246	0.5	100	70	70	70		
Monographella nivalis	6.67	12.8	100	100	100	100	6.8	0.016
	2.22	4.3	100	100	100	100		
	0.739	1.4	100	100	100	100		
	0.246	0.5	100	100	100	100		

Supplementary Table 14. BP80 determination for compound **1a**.

Compound 1a								
**Pathogen tested**	**Rate of application in ppm**	**Rate of application in uM**	**% Pest control**				**pBP80**	**BP80 (uM)**
			**Test 1**	**Test 2**				
				replicate 1	replicate 2	replicate 3		
Erysiphe graminis	22.2	42.6	100	90	100	100	4.4	3.98
	7.39	14.2	50	20	50	70		
	2.46	4.7	20	0	0	20		
	0.819	1.6	0	0	0	0		
Puccinia recondita	22.2	42.6	90	20	70	100	4.4	0.40
	7.39	14.2	20	20	0	0		
	2.46	4.7	20	0	0	0		
	0.819	1.6	0	0	0	0		
Botrytis cinerea	6.67	12.8	100	90	100	100	4.9	1.26
	2.22	4.3	50	70	70	70		
	0.739	1.4	20	20	20	20		
	0.246	0.5	20	0	0	0		
Mycosphaerella arachidis	6.67	12.8	90	100	70	90	4.9	1.26
	2.22	4.3	0	20	20	20		
	0.739	1.4	0	0	0	0		
Septoria tritici	6.67	12.8	20	20	70	50	0	1000
	2.22	4.3	0	0	0	0		
	0.739	1.4	0	0	0	0		
	0.246	0.5	0	0	0	0		
Monographella nivalis	6.67	12.8	100	100	90	90	4.9	1.26
	2.22	4.3	50	50	50	50		
	0.739	1.4	20	0	0	0		
	0.246	0.5	20	0	0	0		

Supplementary Table 15. BP80 determination for compound **1b**.

Compound 1b								
**Pathogen tested**	**Rate of application in ppm**	**Rate of application in uM**	**% Pest control**				**pBP80**	**BP80 (uM)**
			**Test 1**	**Test 2**				
				replicate 1	replicate 2	replicate 3		
Erysiphe graminis	22.2	42.6	100	100	100	100	5.8	0.16
	7.39	14.2	100	100	100	100		
	2.46	4.7	100	90	100	90		
	0.819	1.6	100	70	50	70		
Puccinia recondita	22.2	42.6	100	100	100	100	4.9	1.26
	7.39	14.2	50	100	90	100		
	2.46	4.7	0	50	90	20		
	0.819	1.6	0	0	0	20		
Botrytis cinerea	6.67	12.8	100	100	100	100	5.4	0.40
	2.22	4.3	90	90	90	100		
	0.739	1.4	50	90	70	70		
	0.246	0.5	0	50	0	0		
Mycosphaerella arachidis	6.67	12.8	100	100	100	100	4.9	1.26
	2.22	4.3	50	90	50	20		
	0.739	1.4	0	0	0	20		
Septoria tritici	6.67	12.8	100	100	100	100	4.9	1.26
	2.22	4.3	70	20	50	90		
	0.739	1.4	50	0	0	20		
	0.246	0.5	0	0	0	0		
Monographella nivalis	6.67	12.8	100	100	100	100	5.4	0.40
	2.22	4.3	100	100	90	100		
	0.739	1.4	70	90	50	20		
	0.246	0.5	20	0	0	20		

Supplementary Table 16. BP80 determination for compound **1c**.

Compound 1c								
**Pathogen tested**	**Rate of application in ppm**	**Rate of application in uM**	**% Pest control**				**pBP80**	**BP80 (uM)**
			**Test 1**	**Test 2**				
				replicate 1	replicate 2	replicate 3		
Erysiphe graminis	22.2	42.6	90	70	70	70	4.4	3.98
	7.39	14.2	0	0	50	20		
	2.46	4.7	0	0	0	20		
	0.819	1.6	0	0	0	0		
Puccinia recondita	22.2	42.6	0	0	0	0	0.05	1000
	7.39	14.2	0	0	0	0		
	2.46	4.7	0	0	0	0		
	0.819	1.6	0	0	0	0		
Botrytis cinerea	6.67	12.8	0	0	0	0	0	1000
	2.22	4.3	0	0	0	0		
	0.739	1.4	0	0	0	0		
	0.246	0.5	0	0	0	0		
Mycosphaerella arachidis	6.67	12.8	0	0	0	0	0	1000
	2.22	4.3	0	0	0	0		
	0.739	1.4	0	0	0	0		
Septoria tritici	6.67	12.8	20	0	0	0	0	1000
	2.22	4.3	0	0	0	0		
	0.739	1.4	0	0	0	0		
	0.246	0.5	0	0	0	0		
Monographella nivalis	6.67	12.8	20	0	0	0	0	1000
	2.22	4.3	20	0	0	0		
	0.739	1.4	0	0	0	0		
	0.246	0.5	0	0	0	0		

Supplementary Table 17. BP80 determination for compound **2**.

Compound 2								
**Pathogen tested**	**Rate of application in ppm**	**Rate of application in uM**	**% Pest control**				**pBP80**	**BP80 (uM)**
			**Test 1**	**Test 2**				
				replicate 1	replicate 2	replicate 3		
Erysiphe graminis	22.2	42.6	100	100	100	100	5.8	0.16
	7.39	14.2	100	100	100	100		
	2.46	4.7	100	100	100	100		
	0.819	1.6	90	100	100	100		
Puccinia recondita	22.2	42.6	100	100	100	100	5.8	0.16
	7.39	14.2	100	100	100	100		
	2.46	4.7	100	100	100	100		
	0.819	1.6	100	90	100	90		
Botrytis cinerea	6.67	12.8	100	100	100	100	5.8	0.16
	2.22	4.3	100	100	100	100		
	0.739	1.4	50	90	90	90		
	0.246	0.5	20	20	20	20		
Mycosphaerella arachidis	6.67	12.8	100	100	100	100	5.4	0.4.
	2.22	4.3	90	90	90	90		
	0.739	1.4	50	0	20	50		
Septoria tritici	6.67	12.8	90	100	100	100	5.4	0.40
	2.22	4.3	70	70	70	70		
	0.739	1.4	20	0	0	0		
	0.246	0.5	0	0	0	0		
Monographella nivalis	6.67	12.8	100	100	100	100	5.4	0.40
	2.22	4.3	100	90	90	90		
	0.739	1.4	70	50	50	50		
	0.246	0.5	50	0	20	0		

Supplementary Table 18. BP80 determination for compound **2a**.

Compound 2a								
**Pathogen tested**	**Rate of application in ppm**	**Rate of application in uM**	**% Pest control**				**pBP80**	**BP80 (uM)**
			**Test 1**	**Test 2**				
				replicate 1	replicate 2	replicate 3		
Erysiphe graminis	22.2	42.6	100	100	100	100	5.8	1.26
	7.39	14.2	100	50	100	50		
	2.46	4.7	0	0	70	0		
	0.819	1.6	0	0	0	0		
Puccinia recondita	22.2	42.6	100	100	100	100	4.9	1.26
	7.39	14.2	50	90	90	100		
	2.46	4.7	0	0	90	0		
	0.819	1.6	0	0	50	0		
Botrytis cinerea	6.67	12.8	50	90	90	90	4.9	1.26
	2.22	4.3	20	50	50	50		
	0.739	1.4	0	0	0	0		
	0.246	0.5	0	0	0	0		
Mycosphaerella arachidis	6.67	12.8	50	20	20	20	0.05	1000
	2.22	4.3	0	0	0	0		
	0.739	1.4	0	0	0	0		
Septoria tritici	6.67	12.8	20	0	0	20	0	1000
	2.22	4.3	0	0	0	0		
	0.739	1.4	0	0	0	0		
	0.246	0.5	0	0	0	0		
Monographella nivalis	6.67	12.8	50	50	70	70	0.05	1000
	2.22	4.3	20	20	20	20		
	0.739	1.4	0	0	0	0		
	0.246	0.5	0	0	0	0		

Combinatorial library WelO5*

Codon list incorporated in the WelO5* site-saturation library. The most abundant codons of *E. coli* were chosen in library design. By replacing the wild-type codons on positions V81, A88, and I161 with the codons in the table, the DNA sequences of the constructed variants can be obtained.

Supplementary Table 19. Codon list incorporated in the WelO5* site-saturation library.

Amino acid	Codon	Amino acid	Codon
A	GCA	M	ATG
C	TGT	N	AAT
D	GAT	P	CCG
E	GAA	Q	CAG
F	TTT	R	CGT
G	GGT	S	AGC
H	CAT	T	ACC
I	ATC	V	GTT
K	AAA	W	TGG
L	CTG	Y	TAT

DNA sequence of the ordered gene fragments:

>Twist_gene_fragment_of_the_WelO5*_site_saturation_library
CCCGTCACCTTTGGCTTATCAGTGAGATATACATATGTCGAACAACACCATCTCGACCAAACCAGCCTTGCATTT
TCTCGACATCAACGCCACCGAAGTCAAGAAATATCCCACTGCAATTCAGGACATCATTATCAATCGCTCATTCGA
TGGCATGATTATTCGGGGAGTCTTTCCTCGCGATACGATGGAGCAGGTTGCTCGTTGCCTGGAAGAAGGGAATGA
TGGCGGCATGAAATCCATCCTGAACAAGAATGAAGAGTTTGGTACGAAA**GTT**GCCCAGATTTATGGCCAT**GCG**AT
TGTTGGCCAATCTCCGGATCTCAAAGACTATTTTGCTAGTTCTGCCATTTTCCGTCAGGCGTGTCGTACCATGTT
TCAGGGTAGCCCGGACTTTGAGGAACAAGTGGAGAGCATTTTCCACTCGTTATCCGGACTGCCCGTAGAGATTCC
GACGGGTCCTGAAGGGCAAACTTACACCCCGGCAACCATTCGTCTGCTGTTAGAAGGCCGCGAA**ATT**GCCGTACA
TGTGGGCAACGACTTTCTTCTGATGCCGGCTGCAAACCATCTGAAAACGTTGCTGGATCTGTCTGATCAACTGTC
GTACTTTATCCCGTTAACAGTGCCGGAAGCAGGTGGTGAATTGGTGGTGTACAACCTGGAATGGAATCCGCAGGA
AGTGGACAAATCAGCGGATCTTCACAAGTACATCGATGAGGTCGAAAGCAAATTCAAAAGCAATCAGAGTCAGAG
TGTTGCGTATGCGCCTGGTCCAGGTGATATGCTCCTGTTCAATGGCGGTCGCTATTATCACCGCGTCAGCGAAGT
AATCGGGAATTCCCCACGTCGCACAATTGGCGGATTTCTGGCGTTCTCAAAAGAGCGCAACAAAATCTACTATTG
GAGCTAACTCGAGCACCAGTGACATCTGGACGCTAAGACCG

green = flanking sequence (Twist)
yellow = flanking sequence including restriction sites
bold = mutation site
underlined = WelO5* gene sequence (ORF)

>WelO5* amino acid sequence
MSNNTISTKPALHFLDINATEVKKYPTAIQDIIINRSFDGMIIRGVFPRDTMEQVARCLEEGNDGGMKSILNKNE
EFGTK**V**AQIYGH**A**IVGQSPDLKDYFASSAIFRQACRTMFQGSPDFEEQVESIFHSLSGLPVEIPTGPEGQTYTPA
TIRLLLEGRE**I**AVHVGNDFLLMPAANHLKTLLDLSDQLSYFIPLTVPEAGGELVVYNLEWNPQEVDKSADLHKYI
DEVESKFKSNQSQSVAYAPGPGDMLLFNGGRYYHRVSEVIGNSPRRTIGGFLAFSKERNKIYYWS-
bold = mutation site

IV. Supplementary References

1. Agarwal, V. *et al.* Biosynthesis of polybrominated aromatic organic compounds by marine bacteria. *Nat. Chem. Biol.* **10**, 640–647 (2014).

2. Zehner, S. *et al.* A regioselective tryptophan 5-halogenase is involved in pyrroindomycin biosynthesis in *Streptomyces rugosporus* LL-42D005. *Chem. Biol.* **12**, 445–452 (2005).

3. Heemstra, J. R. & Walsh, C. T. Tandem action of the O_2- and $FADH_2$-dependent halogenases KtzQ and KtzR produce 6,7-dichlorotryptophan for kutzneride assembly. *J. Am. Chem. Soc.* **130**, 14024–14025 (2008).

4. Menon, B. R. K. *et al.* Structure and biocatalytic scope of thermophilic flavin-dependent halogenase and flavin reductase enzymes. *Org. Biomol. Chem.* **14**, 9354–9361 (2016).

5. Zeng, J. & Zhan, J. Characterization of a tryptophan 6-halogenase from *Streptomyces toxytricini*. *Biotechnol. Lett.* **33**, 1607–1613 (2011).

6. Kirner, S. *et al.* Functions encoded by pyrrolnitrin biosynthetic genes from *Pseudomonas fluorescens*. *J. Bacteriol.* **180**, 1939–1943 (1998).

7. Yeh, E., Garneau, S. & Walsh, C. T. Robust *in vitro* activity of RebF and RebH, a two-component reductase/halogenase, generating 7-chlorotryptophan during rebeccamycin biosynthesis. *Proc. Natl. Acad. Sci. U. S. A.* **102**, 3960–3965 (2005).

8. Seibold, C. *et al.* A flavin-dependent tryptophan 6-halogenase and its use in modification of pyrrolnitrin biosynthesis. *Biocatal. Biotransformation* **24**, 401–408 (2006).

9. Wang, S. *et al.* Functional characterization of the biosynthesis of Radicicol, an Hsp90 inhibitor resorcylic acid lactone from Chaetomium chiversii. *Chem. Biol.* **15**, 1328–1338 (2008).

10. Fraley, A. E. *et al.* Function and structure of MalA/MalA', iterative halogenases for late-stage C-H functionalization of indole alkaloids. *J. Am. Chem. Soc.* **139**, 12060–12068 (2017).

11. Zeng, J. & Zhan, J. A novel fungal flavin-dependent halogenase for natural product biosynthesis. *ChemBioChem* **11**, 2119–2123 (2010).

12. Neumann, C. S., Walsh, C. T. & Kay, R. R. A flavin-dependent halogenase catalyzes the chlorination step in the biosynthesis of *Dictyostelium* differentiation-inducing factor 1. *Proc. Natl. Acad. Sci. U. S. A.* **107**, 5798–5803 (2010).

13. Shepherd, S. A. *et al.* Extending the biocatalytic scope of regiocomplementary flavin-dependent halogenase enzymes. *Chem. Sci.* **6**, 3454–3460 (2015).

14. Payne, J. T., Poor, C. B. & Lewis, J. C. Directed evolution of RebH for site-selective halogenation of large biologically active molecules. *Angew. Chem. Int. Ed.* **54**, 4226–4230 (2015).

15. Andorfer, M. C., Park, H. J., Vergara-Coll, J. & Lewis, J. C. Directed evolution of RebH for catalyst-controlled halogenation of indole C-H bonds. *Chem. Sci.* **7**, 3720–3729 (2016).

16. Poor, C. B., Andorfer, M. C. & Lewis, J. C. Improving the stability and catalyst lifetime of the halogenase RebH by directed evolution. *ChemBioChem* **15**, 1286–1289 (2014).

17. Hillwig, M. L. & Liu, X. A new family of iron-dependent halogenases acts on freestanding substrates. *Nat. Chem. Biol.* **10**, 921–923 (2014).

18. Zhu, Q. & Liu, X. Characterization of non-heme iron aliphatic halogenase WelO5* from Hapalosiphon welwitschii IC-52-3: identification of a minimal protein sequence motif that confers enzymatic chlorination specificity in the biosynthesis of welwitindolelinones. *Beilstein J. Org. Chem.* **13**, 1168–1173 (2017).

19. Hillwig, M. L., Zhu, Q., Ittiamornkul, K. & Liu, X. Discovery of a promiscuous non-heme iron halogenase in ambiguine alkaloid biogenesis: implication for an evolvable enzyme family for late-stage halogenation of aliphatic carbons in small molecules. *Angew. Chem. Int. Ed.* **55**, 5780–5784 (2016).

20. Mitchell, A. J. *et al.* Structure-guided reprogramming of a hydroxylase to halogenate its small molecule substrate. *Biochemistry* **56**, 441–444 (2017).

21. Fox, R. J. *et al.* Improving catalytic function by ProSAR-driven enzyme evolution. *Nat. Biotechnol.* **25**, 338–344 (2007).

22. Feng, X., Sanchis, J., Reetz, M. T. & Rabitz, H. Enhancing the efficiency of directed evolution in focused enzyme libraries by the adaptive substituent reordering algorithm. *Chem. Eur. J.* **18**, 5646–5654 (2012).

23. Saito, Y. *et al.* Machine-learning-guided mutagenesis for directed evolution of fluorescent proteins. *ACS Synth. Biol.* **7**, 2014–2022 (2018).

24. Cadet, F. *et al.* A machine learning approach for reliable prediction of amino acid interactions and its application in the directed evolution of enantioselective enzymes. *Sci. Rep.* **8**, (2018).

25. Wu, Z., Jennifer Kan, S. B., Lewis, R. D., Wittmann, B. J. & Arnold, F. H. Erratum: Machine learning-assisted directed protein evolution with combinatorial libraries. *Proc. Natl. Acad. Sci. U. S. A.* **117**, 788–789 (2020).

26. Vornholt, T. *et al.* Systematic engineering of artificial metalloenzymes for new-to-nature reactions. *Sci. Adv.* **7**, eabe4208 (2021).

27. Waterhouse, A. *et al.* SWISS-MODEL: homology modelling of protein structures and complexes. *Nucleic Acids Res.* **46**, W296–W303 (2018).

28. Jumper, J. *et al.* Highly accurate protein structure prediction. *Nature* (2021). doi:10.1038/s41586-021-03819-2

29. Trott, O. & Olson, A. J. AutoDock Vina: Improving the speed and accuracy of docking with a new scoring function, efficient optimization, and multithreading. *J. Comput. Chem.* **31**, 455–461 (2010).

30. Stourac, J. *et al.* Caver Web 1.0: Identification of tunnels and channels in proteins and analysis of ligand transport. *Nucleic Acids Res.* **47**, W414–W422 (2019).

31. Zirkle, R., Ligon, J. M. & Molnár, I. Heterologous production of the antifugal polyketide antibiotic soraphen A of *Sorangium cellulosum* So ce26 in Streptomyces lividans. *Microbiology* **150**, 2761–2774 (2004).

32. Bedorf, N. *et al.* Mikrobiologisches Verfahren zur Herstellung agrarchemisch verwendbarer mikrobizider makrozyklischer Lactonderivate. EP 358606 A2 (1990).

33. Sutter, M., O'Sullivan, A., Hoefle, G., Boehlendorf, B. & Kiffe, M. Sopharen c Markolid-Derivate und ihre Verwendung als Mikrobizide. EP540469 (1993).
