# Supplementary material for: Algorithm-aided engineering of aliphatic halogenase WelO5* for the asymmetric late-stage functionalization of soraphens
Source: Nat Commun. 2022 Jan 18;13:371. doi: 10.1038/s41467-022-27999-1 (PMC8766452; doi:10.1038/s41467-022-27999-1)
Supplement: Supplementary file 3 — Reporting Summary [file 41467_2022_27999_MOESM3_ESM.pdf]

nature research

Corresponding author(s): Rebecca Buller

Last updated by author(s): Nov 24, 2021

Reporting Summary

Nature Research wishes to improve the reproducibility of the work that we publish. This form provides structure for consistency and transparency in reporting. For further information on Nature Research policies, see our Editorial Policies and the Editorial Policy Checklist.

Statistics

For all statistical analyses, confirm that the following items are present in the figure legend, table legend, main text, or Methods section.

n/a	Confirmed	
[ ]	[x]	The exact sample size (*n*) for each experimental group/condition, given as a discrete number and unit of measurement
[ ]	[x]	A statement on whether measurements were taken from distinct samples or whether the same sample was measured repeatedly
[x]	[ ]	The statistical test(s) used AND whether they are one- or two-sided<br>*Only common tests should be described solely by name; describe more complex techniques in the Methods section.*
[x]	[ ]	A description of all covariates tested
[x]	[ ]	A description of any assumptions or corrections, such as tests of normality and adjustment for multiple comparisons
[ ]	[x]	A full description of the statistical parameters including central tendency (e.g. means) or other basic estimates (e.g. regression coefficient) AND variation (e.g. standard deviation) or associated estimates of uncertainty (e.g. confidence intervals)
[x]	[ ]	For null hypothesis testing, the test statistic (e.g. *F*, *t*, *r*) with confidence intervals, effect sizes, degrees of freedom and *P* value noted<br>*Give P values as exact values whenever suitable.*
[x]	[ ]	For Bayesian analysis, information on the choice of priors and Markov chain Monte Carlo settings
[x]	[ ]	For hierarchical and complex designs, identification of the appropriate level for tests and full reporting of outcomes
[x]	[ ]	Estimates of effect sizes (e.g. Cohen's *d*, Pearson's *r*), indicating how they were calculated

Our web collection on statistics for biologists contains articles on many of the points above.

Software and code

Policy information about availability of computer code

Data collection	Agilent OpenLAB CDS (2.4, Build 2.204.0.661) was used for the aquisition of LC-MS data. NMRs were recorded either on a Bruker 400 MHz spectrometer or a Bruker 600 MHz spectrometer with accompanying software.
Data analysis	Agilent OpenLAB CDS was used for integration of chromatograms. Data was handled using Microsoft Excel (Version 2102, Build 13801.21004) and Python (3.7.4) Plotly (5.3.1). GraphPad Prism (8.4.0) was used for non-linear curve fitting. ACD/Spectrus was used for the analysis of NMR data. TIBCO Spotfire, standard operations, was used for the analysis of the biological samples. For illustration purposes ChemDraw 19.0, Adobe Illustrator 24.1.1, Microsoft Excel (Version 2102, Build 13801.21004), Microsoft Word (Version 2102, Build 13801.21004), Pymol 2.4.1 and Plotly (5.3.1) Python (3.7.4) were used. SWISS-MODEL (https://swissmodel.expasy.org/) and AlphaFold (doi:10.1038/s41586-021-03819-2) were used for homology modelling. Chimera 1.13.1 and AutoDockVina (J. Comput. Chem. 31, 455–461 (2010)) were used for docking. Machine learning scripts for the prediction of enzyme function, please access https://github.com/ccbiozhaw/MLevo.

For manuscripts utilizing custom algorithms or software that are central to the research but not yet described in published literature, software must be made available to editors and reviewers. We strongly encourage code deposition in a community repository (e.g. GitHub). See the Nature Research guidelines for submitting code & software for further information.

Data

Policy information about availability of data

All manuscripts must include a data availability statement. This statement should provide the following information, where applicable:

- Accession codes, unique identifiers, or web links for publicly available datasets
- A list of figures that have associated raw data
- A description of any restrictions on data availability

Data availability: Source data are provided with this paper. WelO5 crystal structure used as template for SWISS-MODEL homology modelling can be accessed via

PDB ID: 5J4R. The authors declare that all the data supporting the findings of this work are available within the article and its Supplementary Information and the provided Source Data.

Field-specific reporting

Please select the one below that is the best fit for your research. If you are not sure, read the appropriate sections before making your selection.

- [x] Life sciences
- [ ] Behavioural & social sciences
- [ ] Ecological, evolutionary & environmental sciences

For a reference copy of the document with all sections, see nature.com/documents/nr-reporting-summary-flat.pdf

Life sciences study design

All studies must disclose on these points even when the disclosure is negative.

Sample size	No sample size calculation was performed to predetermine sample size. A sample size of > 3 was used for all of the in vitro experiments described. Three replicate measurements are sufficient to have confindence in average measured values and to determine a standard deviation or standard error that allows for statistical differentiation between experiments. BP80 values were derived through dilution series in two biological replicates (1st measurement series, N = 1, 2nd measurement series, N = 3 (technical replicates).
Data exclusions	No data were excluded from the analyses.
Replication	All reported attempts at replication were successful (at least 3 independent experiments).<br>Activity of improved enzyme variants was intrinsically verified through in vitro characterization (ttn; Michaelis-Menten kinetics) and preparative biocatalysis reactions yielding derivatized product.
Randomization	Randomization was not neccessary for the experimental part of the target-focussed study as it is not applicable to the in vitro experiments performed here. Nevertheless, appropriate control experiments were performed. For the prediction of improved enzyme function, we conducted ML experiments by splitting the data randomly into ten subsets which were then each used for validation once. Where possible, we also fixed the random seed to ensure reproducibility, such as for the splitting and training procedure.
Blinding	Blinding was not necessary since this study does not involve animal or human subjects or group allocations.

Reporting for specific materials, systems and methods

We require information from authors about some types of materials, experimental systems and methods used in many studies. Here, indicate whether each material, system or method listed is relevant to your study. If you are not sure if a list item applies to your research, read the appropriate section before selecting a response.

Materials & experimental systems

n/a	Involved in the study	
[x]	[ ]	Antibodies
[x]	[ ]	Eukaryotic cell lines
[x]	[ ]	Palaeontology and archaeology
[x]	[ ]	Animals and other organisms
[x]	[ ]	Human research participants
[x]	[ ]	Clinical data
[x]	[ ]	Dual use research of concern

Methods

n/a	Involved in the study	
[x]	[ ]	ChIP-seq
[x]	[ ]	Flow cytometry
[x]	[ ]	MRI-based neuroimaging
